# The effects of chicory inulin-type fructans supplementation on weight management outcomes: systematic review, meta-analysis, and meta-regression of randomized controlled trials

**DOI:** 10.1016/j.ajcnut.2024.09.019

**Published:** 2024-09-21

**Authors:** Raylene A Reimer, Stephan Theis, Yoghatama Cindya Zanzer

**Affiliations:** 1 Faculty of Kinesiology, University of Calgary, Calgary, Alberta, Canada; 2 Department of Biochemistry and Molecular Biology, Cumming School of Medicine, University of Calgary, Calgary, Alberta, Canada; 3 Alberta Children’s Hospital Research Institute, Heritage Medical Research Building, Calgary, Alberta, Canada; 4 BENEO Institute c/o BENEO GmbH, Obrigheim/Pfalz, Germany

**Keywords:** inulin, oligofructose, weight management, meta-analysis, meta-regression, systematic review

## Abstract

**Background:**

Excess body weight and adiposity can adversely affect metabolic health. Prebiotics such as inulin-type fructans (ITFs) from chicory root are known to modulate gut microbiota and may improve body weight regulation.

**Objectives:**

This study aimed to assess evidence for chicory ITF supplementation to support weight management.

**Methods:**

Eligible articles (initial search to 2021, updated to February 2023) were searched from EMBASE, MEDLINE (PubMed), and Cochrane Library. Data on primary (body weight) and secondary outcomes [body mass index (BMI), total fat mass, body fat percentage, and waist circumference] were extracted by 2 reviewers independently. Random-effects model using inverse-variance method was used. Subgroup analysis (health status and ITF type) and meta-regression (dose and duration) were evaluated.

**Results:**

A total of 32 eligible studies were included. Chicory ITF significantly reduced body weight [mean difference (MD): −0.97 kg; 95% CI: −1.34, −0.59); *n* = 1184] compared with placebo. ITF favored overall effects reduction in BMI (MD: −0.39 kg/m^2^; 95% CI: −0.57, −0.20; *n* = 985), fat mass (MD: −0.37 kg; 95% CI: −0.61, −0.13; *n* = 397), waist circumference (MD: −1.03 cm; 95% CI: −1.69, −0.37; *n* = 604), and for intervention duration of >8 wk, body fat percentage (MD: −0.78%; 95% CI: −1.17, −0.39; *n* = 488). Except for considerable heterogeneity in body weight (*I*^2^: 73%) and body fat percentage (*I*^2^: 75%), all other outcomes had negligible to moderate heterogeneity. Significant reduction in body weight, BMI, and waist circumference was evident irrespective of participants' health status. There was minimal evidence that dose, duration, or type of ITF influenced the magnitude of reductions in outcomes.

**Conclusions:**

Chicory ITF supplementation may benefit weight management by reducing body weight, BMI, fat mass, waist circumference, and, to a certain extent, body fat percentage.

This systematic review with meta-analysis was registered at PROSPERO as CRD42020184908.

## Introduction

The increasing prevalence of overweight and obesity worldwide represents a major public health challenge for societies and health care systems. The World Obesity Atlas 2023 Report estimates that 38% of the world’s population had overweight or obesity in 2020, a number that is expected to rise to 51% by 2035 [1]. The nearly United States $2 billion economic impact of overweight and obesity in 2020 is projected to double by 2035 and encompass not only health care costs but also lost productivity, leading to reduced global gross domestic product [1]. Obesity predisposes individuals to related health threats including type 2 diabetes, cardiovascular disease, and certain cancers [2].

Obesity is a complex multifactorial disease that is difficult to treat and has high recidivism [3]. In addition to the recent advances made in pharmacotherapies, surgical options, and behavioral interventions [4], there is growing interest in the gut microbiota and its potential role in obesity management. The composition of the gut microbiota is altered in obesity, and diet is known to be a critical factor in shaping the gut microbial community [5]. Of the dietary factors that modulate the gut microbiota, prebiotics are well known for their ability to serve as nutrients for beneficial microorganisms harbored by the host. Prebiotics are currently defined as “a substrate that is selectively utilized by host microorganisms conferring a health benefit” [6]. Inulin, fructo-oligosaccharides (FOSs), and galacto-oligosaccharides (GOSs) are considered accepted or proven prebiotics and exhibit the most robust evidence for health benefits [6], whereas candidate prebiotics require more evidence to meet the current prebiotic definition. The inulin-type fructans (ITFs), namely inulin and FOS or their mixture, can be derived from chicory root, agave, artichokes, or yacon.

Clinical trials with prebiotics have demonstrated improvements in several obesity-related outcomes. For example, significant reductions in body weight and body fat mass were seen when adults with overweight or obesity consumed 21 g of oligofructose per day for 12 wk compared with placebo [7]. Similarly, consumption of 12 or 18 g/d of GOSs by adults with overweight significantly reduced body weight and BMI (kg/m^2^) [8]. Improvements in weight-related outcomes following prebiotic consumption are plausibly linked to shifts in the gut microbiota. Enrichment in the abundance of *Bifidobacterium* spp is characteristic of prebiotic consumption but other bacteria linked to improved body weight regulation can also be altered including reduced abundance of *Desulfovibrio* and *Clostridium sensu stricto* species [6,9,10]. Prebiotics such as inulin and FOSs serve as a substrate for beneficial gut microbiota, which in turn produce short-chain fatty acids (SCFAs) such as acetate, propionate, and butyrate [9]. SCFA produced by beneficial gut microbiota could interact with receptors on enteroendocrine cells to promote indirect signaling to the brain via systemic circulation or through vagal pathways by stimulating the secretion of gut hormones such as glucagon-like peptide (GLP)-1 and peptide tyrosine–tyrosine (PYY), which regulate appetite and energy metabolism [10].

Collectively, the evidence for prebiotics to influence obesity-related outcomes has been assessed in several systematic reviews and meta-analyses. These include those showing significant beneficial effects of prebiotics (not distinguishing between accepted and candidate prebiotics) for reducing both body weight and fat mass [11] and those showing no significant effect of prebiotic products [12]. Other systematic reviews and meta-analyses have evaluated the effect of isolated soluble fiber supplements, many of which were studies with inulin, FOSs, or GOSs, and reported reductions in body weight, body fat, and BMI in participants with overweight or obesity [13] and greater reductions in body weight, BMI, and waist circumference in participants with overweight or obesity in studies of ≥12-wk duration [14]. Importantly, systematic reviews conducted to date that are related to obesity-related outcomes have not strictly focused on studies investigating the effects of accepted/proven prebiotics, and specifically chicory ITFs. Compared with fructans derived from other plant sources (e.g., agave, artichoke, and yacon), ITF derived from chicory roots have a unique branching and degree of polymerization. Depending on the distribution of chain lengths and mode of production, ITF derived from chicory can be classified into inulin and oligofructose and, to a certain extent, a mixture of both. Although oligofructose consists of polyfructose chains linked together by β (2–1)-linkages with monomeric units ranging between 3 and 9, inulin is a mixture of fructan chains linked together by β(2–1)-linkages with a degree of polymerization (DP) between 2 and 60 or even more [15]. In contrast, fructans from other sources such as agave have distinct complex mixtures containing different types of linkages [e.g., β(2–1) and β(2–6)] and units [e.g., outer (graminans fructans) and inner glucose (neoseries fructans)] [16]. The proportion of this complex agavin structure also changes throughout the developmental ages of the agave plants [17]. On the contrary, short-chain FOSs are produced enzymatically from sucrose and consist of a terminal glucose molecule linked to fructose molecules by a β1–2 bond with a low DP ranging between 3 and 5, whereas oligofructose is produced from chicory inulin by partial enzymatic hydrolysis with a DP ranging between 2 and 9. We hypothesized that the structural differences of various fructans coming from different sources may distinctively affect the gut microbiota profile and confer a distinct health benefit to the host.

Therefore, our objective was to systematically review and quantitatively evaluate the effect of ITF supplementation specifically from chicory root on components of weight management in humans irrespective of health status, in comparison with placebo/control. We hypothesized that chicory ITF supplementation would reduce body weight, BMI, total body fat, body fat percentage, and waist circumference and that study duration, intervention dose, and type of ITF would influence the outcomes.

## Methods

This review was designed, conducted, and reported according to the Preferred Reporting Items for Systematic Reviews and Meta-Analyses (PRISMA) and followed the Cochrane Handbook for Systematic Reviews of Interventions [18]. The protocol was registered at PROSPERO (identified as CRD42020184908).

### Data sources and search strategy

A systematic search was performed on EMBASE, MEDLINE, and Cochrane Library from January to August 2021, with an additional update in February 2023. Databases were searched to identify studies comparing the effects of chicory ITF on the primary outcome of body weight and the secondary outcomes of BMI, total body fat, body fat percentage, and waist circumference. The literature search was conducted without any language restriction. The full details of the search terms and strings used for the literature search are outlined in Supplemental Table 1.

### Study selection

Potential eligible studies were included based on criteria based on the PICOS framework (Table 1). Studies were included if they met the following: population (adults), intervention (chicory ITF), comparators (placebo/control), outcomes (reporting 1 or more parameter of weight management), and study design (randomized controlled trials). Studies were excluded if they were conducted in animals or in children and adolescents, evaluated nonchicory root sources of ITF or synbiotics, did not have an appropriate control, did not report a parameter of weight management, or were nonrandomized controlled trials.

**TABLE 1:** Eligibility criteria following the PICOS approach.

PICOS	Inclusion(s)	Exclusion(s)
Participants	Adult population; limited to trials/clinical studies performed in humans	Trials/clinical studies evaluating chicory ITF in children and adolescents and animal studies were excluded
Intervention	Chicory ITF irrespective of their degree of polymerization chain length (e.g., oligofructose, inulin, mixture of oligofructose and inulin)	• Trials/clinical studies evaluating fructans obtained from sources other than chicory root (e.g., agave, artichokes, and yacon) • Trials/clinical studies using chicory ITF mixed with probiotics (synbiotics), unless there was an arm of ITF alone • Trials/clinical studies using modified inulin with other active compounds (e.g., inulin propionate esters)
Comparators	Placebo/control (e.g., maltodextrin, sucrose, dextrose, cellulose, starch, and control meal without chicory ITF)	• Trials/clinical studies evaluating chicory ITF without a placebo or comparable control • Trials/clinical studies comparing chicory ITF as prebiotics with probiotics without an appropriate placebo/control
Outcomes	Trials/clinical studies reporting 1 or more of the 5 aspects of weight management (body weight, BMI, total body fat, body fat percentage, and waist circumference)	Trials/clinical studies not reporting 1 or more weight management aspect (body weight, BMI, total body fat, body fat percentage, and waist circumference)
Study design	Only randomized controlled trials (both parallel and crossover design)	Nonrandomized controlled trials

Abbreviations: BMI, body mass index; ITF, inulin-type fructan; PICOS, participants, intervention, comparators, outcomes, and study design.

### Data extraction

Literature screening was conducted by 2 reviewers (YCZ, ST) independently. Any disagreement with a particular study was resolved with a consensus. In the case of unresolved disagreement, a consensus was then reached with the third reviewer (RAR). Search results from databases were imported into COVIDENCE literature review management software to facilitate the screening process for reviewers. Data from included studies were extracted in a tabular Excel format including information on authors, year of publication, country, study design, study population, intervention, control, dose, duration, and summary statistics of weight management outcome parameters both at baseline and end-of-intervention. Data presented as a figure were extracted using WebPlotDigitizer as recommended by the Cochrane Guideline [18]. Change from baseline and its respective SD from both ITF supplementation and control was used to synthesize the overall effect size [mean difference (MD)]. Studies that reported either median with IQ or mean with 95% CIs as point estimate were then used to construct respective mean and SD. Likewise, SD of change from baseline, when not reported explicitly in the study, was reconstructed using *P* value and *t* value statistics. Where there was a lack of information on statistics (*P* value, *t* value) in the article, the SD of change was then reconstructed using a correlation coefficient derived from another similar study. Equations used for data transformation are outlined in Supplemental Table 2.

### Risk of bias assessment

Risk of bias (RoB) of all included studies was assessed by 2 reviewers (RAR and YCZ) independently using the revised Cochrane RoB tool (version 2) [19]. Any discrepancies were discussed and resolved with a third independent reviewer. Based on 5 domains (bias arising from randomization process, deviations from intended interventions, missing outcome data, measurement of the outcome, and selection of the reported result), studies were judged to have a low, some concerns, or high RoB. Studies were rated at an overall low RoB if all domains (excluding bias owing to missing data) were rated at low RoB.

### Reporting/publication bias

Visual inspection of funnel plots and Egger test were used to assess reporting bias. In the case of suspected publication bias (asymmetry of the funnel plot or significance level *P* < 0.05 of the Egger test), the Duval and Tweedie trim-and-fill method was performed [20].

### Data synthesis and statistical analysis

Expecting populations with diverse characteristics (e.g., health status, ethnic background, and other factors), we applied an inverse-variance method with random-effects model for pooling effect sizes in the meta-analysis. To account for within-study and between-study variances, we applied the Sidik–Jonkman estimator with Hartung–Knapp adjustment. Adding the latter adjustment into the model also provides better coverage for studies with small sample sizes. The change of MD from the baseline value and 95% CI of outcome parameters were then pooled. The statistical significance level was set to a 2-sided *P* value of <0.05. Statistical analysis was performed using *'meta'* and *'metafor'* packages within R-environment ver. 3.2.4. Prespecified subgroup analyses based on health status [e.g., apparently healthy (overweight, obese, and prediabetic) and diseased], inulin type (e.g., oligofructose, oligofructose-enriched inulin, and inulin], dose (e.g., dose of >10 and ≤10 g/d), and duration (e.g., duration of >8 and ≤8 wk) were evaluated. The rationale for these subgroup analyses includes a potential greater response in those with a more perturbed metabolic state, structural differences between oligofructose and long-chain inulin possibly affecting metabolic outcomes, a dose-dependent response, and the potential for a threshold of duration to observe benefits. We defined health status of participants as those with either no diagnosed condition or those with a chronic disease diagnosis, which included studies of participants with nonalcoholic fatty liver disease, nonalcoholic steatohepatitis, polycystic ovarian syndrome, ischemic heart disease, and type 2 diabetes [21]. Studies that enrolled participants with overweight or obesity but no other diagnosed condition were analyzed together with studies that enrolled healthy participants without overweight or obesity (i.e., BMI: 18.50–24.99). Furthermore, we conducted meta-regression to evaluate the moderating impacts of the covariates dose and duration on weight management outcome parameters. In conducting meta-regression, we used mixed-effects model [22] accounting for fixed effect (the β coefficient) and random-effects terms (ζ_*k*_), where *ϵ*_*k*_ is sampling error, ζ_*k*_ is between-study heterogeneity, and subscript *k* indicates that a value varies from study to study using the following regression model:

$${\hat{\theta }}_{k}=\theta +\beta x_{k}+\epsilon_{k}+\zeta_{k}$$

## Results

### Characteristics of included studies

**FIGURE 1:**
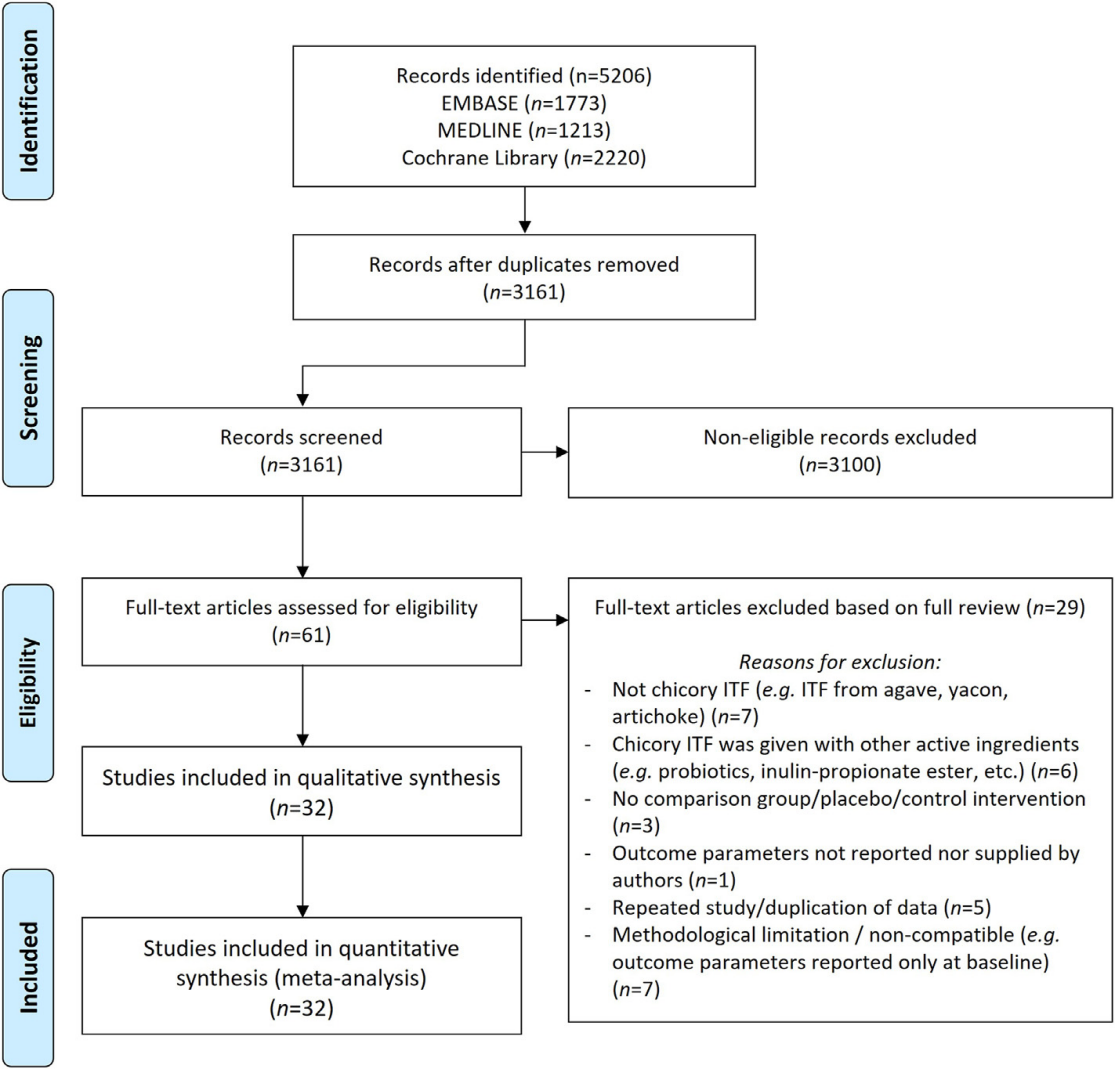
PRISMA flow diagram. ITF, inulin-type fructan.

The PRISMA flow diagram of trial selection is depicted in Figure 1. A total of 3161 articles were identified and 61 articles retrieved for full-text review. In total, 32 studies fulfilled the inclusion criteria and were eligible for meta-analysis (Table 2) [7,[23], [24], [25], [26], [27], [28], [29], [30], [31], [32], [33], [34], [35], [36], [37], [38], [39], [40], [41], [42], [43], [44], [45], [46], [47], [48], [49], [50], [51], [52], [53]], whereas 29 studies were excluded. Excluded studies and the reasons for exclusion are outlined in Supplemental Table 3. Studies were conducted globally and represented 4 regions in the world, namely in Asia (China, India, Iran, and Kuwait); Australia and Pacific (Australia and New Zealand); Europe (Belgium, France, Italy, the Netherlands, Norway, Spain, Turkey, and the United Kingdom); and the Americas (Canada and Mexico). The majority of trials (84%; *n* = 27 studies) were performed using a parallel design, whereas only a few (16%; *n* = 5 studies) used a crossover design. Of the included studies, 14 trials were conducted in participants with overweight or obesity [[7], [27], [29], [30], [36], [38], [39], [44], [45], [47], [48], [49], [50], [53]], 5 trials were in participants with type 2 diabetes (T2D) [[26], [34], [40], [41], [46]], 2 trials were in participants with prediabetes [[42], [43]], whereas another 10 trials enrolled healthy individuals [[23], [24], [52]], or participants with nonalcoholic fatty liver disease [[28], [35], [37]], nonalcoholic steatohepatitis [32], ischemic heart disease [51], polycystic ovary syndrome [25], or peritoneal dialysis [31]. The majority of the studies (*n* = 24) require subjects to maintain their regular lifestyle (e.g., under *ad libitum* diet and normal physical activity) during the ITF supplementation, whereas only a few studies (*n* = 8) involved subjects following an energy-restricted diet alongside the ITF supplementation. The daily dose and intervention duration of chicory ITF supplementation varied among included trials with an overall median dose of 10 g/d (range: 3–30 g/d) and median intervention duration of 12 wk (range: 4–36 wk).

**TABLE 2:** Characteristics of the included studies.

Study	Country	Trial design	Health status	Sample size, *n* (% F)	Intervention	Control	Dose (g/d)	Duration (wk)	Outcomes included in the meta-analysis
Alptekin et al. [23], 2022	Turkey	Parallel	Healthy	16 (100)	Inulin	Maltodextrin	6	5	BW, BMI, BFP, WC
Williams et al. [24], 2022	Australia	Parallel	Healthy	40 (78)	OF-enriched inulin	Maltodextrin	12	6	BW, BMI, BFP, WC
Ziaei et al. [25], 2022	Iran	Parallel	PCOS	75 (100)	Inulin (a); OF-enriched inulin (b)	Maltodextrin	10	12	BW, BMI
Birkeland et al. [26], 2021	Norway	Crossover	T2D	29 (41)	OF-enriched inulin	Maltodextrin	16	6	BW
Vaghef-Mehrabany et al. [27], 2021	Iran	Parallel	Obesity with depression	45 (100)	Inulin	Maltodextrin	10	8	BW, BMI, TF, BFP, WC
Chong et al. [28], 2020	New Zealand	Parallel	NAFLD	40 (48)	Inulin	Maltodextrin	8	12	BW, BMI
Hassan et al. [29], 2020	Italy	Parallel	Obesity	30 (80)	Inulin	Pasta placebo	2%	12	BW, BMI
Hiel et al. [30], 2020	Belgium	Parallel	Obesity	106 (61)	Inulin	Maltodextrin	16	12	BW, BMI, TF, WC
Li et al. [31], 2020	China	Crossover	Peritoneal dialysis	15 (40)	OF-enriched inulin	Maltodextrin	10	12	BMI
Bomhof et al. [32], 2019	Canada	Parallel	NASH	14 (43)	Oligofructose	Maltodextrin	16 (8 g for first 12 wk)	36	BW, BMI, TF, BFP, WC
Salmean [33], 2017	Kuwait	Parallel	Healthy	36 (100)	Inulin	Flavored water	16	1	BW, BMI
Ghavami et al. [34], 2018	Iran	Parallel	T2D	46 (57)	Inulin	Starch	10	6	BW, BMI, WC
Javadi et al. [35], 2018	Iran	Parallel	NAFLD	38 (24)	Inulin	Maltodextrin	10	12	BW, BMI, TF, WC
Pol et al. [36], 2018	Netherlands	Parallel	Overweight/obesity	55 (65)	Oligofructose	Placebo snackbar	16	12	BW, TF, BFP, WC
Behrouz et al. [37], 2017	Iran	Parallel	NAFLD	59 (31)	Oligofructose	Maltodextrin	16	12	BW, BMI, BFP
Castro-Sánchez et al. [38], 2017	Mexico	Crossover	Obesity	16 (NR)	Inulin	Dextrose	9	8	BW, BFP, WC
Reimer et al. [39], 2017	Canada	Parallel	Overweight/obesity	53 (54)	OF-enriched inulin	Placebo snackbar	16 (8 g for first 2 wk)	12	BW, BMI, TF, BFP, WC
Roshanravan et al. [40], 2017	Iran	Parallel	T2D	30 (57)	Inulin	Starch	10	6.5	BW, BMI, WC
Dehghan et al. [41], 2016	Iran	Parallel	T2D	49 (100)	OF-enriched inulin	Maltodextrin	10	8	BW, BMI, WC
Guess et al. [42], 2016	United Kingdom	Crossover	Prediabetes	34 (41)	OF-enriched inulin	Cellulose	30 (10 g for first 2 wk)	6	BW
Guess et al. [43], 2015	United Kingdom	Parallel	Prediabetes	38 (44)	OF-enriched inulin	Cellulose	30 (10 g for first 2 wk)	18	BW
Tripkovic et al. [44], 2015	United Kingdom	Crossover	Overweight	10 (0)	Inulin	Placebo bread roll	15	4	BW, BMI, BFP, WC
Daud et al. [45], 2014	United Kingdom	Parallel	Overweight/obesity	22 (73)	Oligofructose	Cellulose	10	6	BW, BMI
Dehghan et al. [46], 2014	Iran	Parallel	T2D	49 (100)	Inulin	Maltodextrin	10	8	BW, BMI
Sheth and Gupta [47], 2014	India	Parallel	Obesity	60 (NR)	Oligofructose	Dextrose	12	12	BW, BMI, BFP, WC
Dewulf et al. [48], 2013	Belgium	Parallel	Obesity	30 (100)	OF-enriched inulin	Maltodextrin	16	12	BMI, BFP
Tovar et al. [49], 2012	Mexico	Parallel	Obesity	59 (100)	Inulin	Control meal	10	12	BW
de Luis et al. [50], 2010	Spain	Parallel	Obesity	30 (87)	Inulin	Placebo biscuit	3	4	BW, BMI, TF
Gomez-Reyes et al. [51], 2010	Mexico	Parallel	IHD	47 (62)	OF-enriched inulin	Placebo bread roll	6.7 (3.4 g for first 2 wk)	12	BMI, BFP
Parnell and Reimer [7], 2009	Canada	Parallel	Overweight/obesity	39 (82)	Oligofructose	Maltodextrin	21	12	BW, TF
Forcheron and Beylot [52], 2007	France	Parallel	Healthy	17 (65)	OF-enriched inulin	Maltodextrin	10	24	BW, TF, BFP
Balcázar-Muñoz et al. [53], 2003	Mexico	Parallel	Obesity	12 (0)	Inulin	Placebo	7	4	BW, BMI

Abbreviations: BFP, body fat percentage; BMI, body mass index; BW, body weight; IHD, ischemic heart disease; NAFLD, nonalcoholic fatty liver disease; NASH, nonalcoholic steatohepatitis; NR, not reported; OF, oligofructose; PCOS, polycystic ovarian syndrome; TF, total fat; T2D, type 2 diabetes; WC, waist circumference.

### Primary outcome (body weight)

**FIGURE 2:**
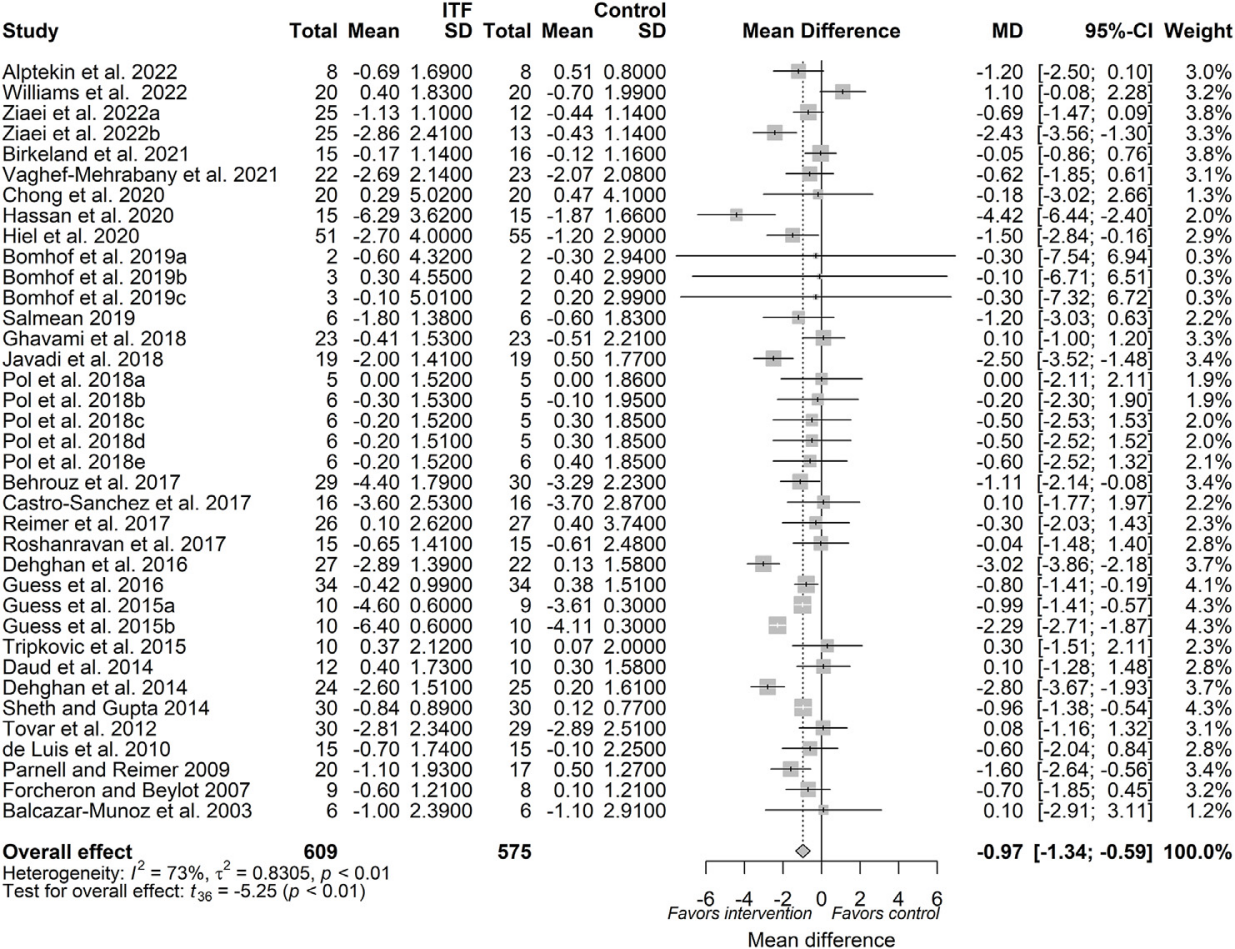
Forest plot of effects of inulin-type fructan (ITF) supplementation on body weight (kg). Values were calculated as baseline-corrected mean difference (MD) and its corresponding 95% CI using random-effects model/inverse-variance method. To account for within-study and between-study variances, the Sidik–Jonkman estimator and Hartung–Knapp adjustment were applied into the model. Interstudy heterogeneity was quantified as *I*^2^. Significance level was set at *P* < 0.05.

The pooled analysis from 29 trials (available as 37 reports, *n* = 1184 subjects) indicated that chicory ITF supplementation significantly reduced body weight (MD: −0.97 kg; 95% CI: −1.34, −0.59; *P* < 0.0001), compared with placebo (Figure 2). Given an existing moderate to substantial between-study heterogeneity (*I*^2^: 73.2%; 95% CI: 62.9, 80.6%; *P* < 0.0001), a prediction interval analysis was then performed. Following analysis, the between-study heterogeneity variance was estimated at ${\hat{\tau }}^{2}$ = 0.83 (95% CI: 0.34, 1.48) with a prediction interval of MD ranging from −2.86 to 0.93, indicating that negative intervention effects cannot be ruled out for future studies. A priori subgroup analysis based on ITF dose showed a significant reduction in body weight both at a dose of ≤10 g/d (MD: −1.06 kg; 95% CI: −1.6, −0.52; *P* < 0.01) and a dose of >10 g/d (MD: −0.84 kg; 95% CI: −1.37, −0.31; *P* < 0.01) (Supplemental Figure 1; Table 3). Meta-regression indicated that the magnitude of body weight reduction was independently influenced by the dose (β_dose_: −0.0097; *P* = 0.69) (Supplemental Figure 2). A significant effect was observed following a priori subgroup analysis based on duration with the effect size on duration of >8 wk (MD: −1.31 kg; 95% CI: −1.8, −0.82; *P* < 0.01) and duration of ≤8 wk (MD: −0.63 kg; 95% CI: −1.19, −0.06; *P* = 0.03) (Table 3; Supplemental Figure 3). A trend based on duration (β_duration_: 0.07; *P* = 0.06) was observed following continuous meta-regression (Supplemental Figure 4). Based on the type of ITF, oligofructose had negligible heterogeneity (*I*^2^: 0.00%) compared with inulin (*I*^2^: 76%) or oligofructose-enriched inulin (*I*^2^: 87%), showing that body weight reduction (MD: −0.84 kg; 95% CI: −1.17, −0.52; *P* < 0.01) following oligofructose supplementation was consistent across the study populations (Table 3; Supplemental Figure 5). A priori subgroup analysis showed that a significant reduction in body weight was independent of subject health status (Table 3; Supplemental Figure 6).

**TABLE 3:** Subgroup analysis of ITF supplementation on weight management.

Study group	Sample size (*n*)	Subgrouping meta-analysis				Heterogeneity, *I*^2^ (%)
		MD (95% CI)	*P* (effect in subgroup)	*P* (subgroup difference)	*P* (overall effect)	
Body weight (kg)						
Overall effect size	1184	−0.97 (−1.34, −0.59)			*<*0.0001	73
Subgrouped based on health status				0.18		
Apparently healthy	753	−0.78 (−1.21, −0.36)	*<*0.01			67
Disease	431	−1.34 (−2.10, −0.57)	*<*0.01			33
Subgrouped based on ITF type				0.57		
Inulin	689	−1.21 (−1.93, −0.49)	*<*0.01			76
Oligofructose	247	−0.84 (−1.17, −0.52)	*<*0.01			0
Oligofructose-enriched inulin	248	−0.77 (−1.57, 0.03)	0.06			87
Subgrouped based on dose				0.53		
Dose ≤ 10 g/d	680	−1.06 (−1.60, −0.52)	*<*0.01			70
Dose > 10 g/d	504	−0.84 (−1.37, −0.31)	*<*0.01			78
Subgrouped based on duration				0.06		
Duration ≤ 8 wk	534	−0.63 (−1.19, −0.06)	0.03			75
Duration > 8 wk	650	−1.31 (−1.80, −0.82)	*<*0.01			68
BMI (kg/m^2^)						
Overall effect size	985	−0.39 (−0.57, −0.20)			0.0002	30
Subgrouped based on health status				0.40		
Apparently healthy	508	−0.32 (−0.6, −0.04)	0.03			38
Disease	477	−0.47 (−0.74, −0.2)	*<*0.01			23
Subgrouped based on ITF type				0.21		
Inulin	630	−0.51 (−0.81, −0.20)	*<*0.01			53
Oligofructose	202	−0.30 (−0.40, −0.19)	*<*0.01			0
Oligofructose-enriched inulin	153	−0.21 (−0.45, 0.03)	0.07			0
Subgrouped based on dose				0.11		
Dose ≤ 10 g/d	626	−0.48 (−0.75, −0.22)	*<*0.01			45
Dose > 10 g/d	359	−0.25 (−0.43, −0.06)	0.01			0
Subgrouped based on duration				0.33		
Duration ≤ 8 wk	403	−0.29 (−0.59, −0.0003)	0.05			36
Duration > 8 wk	582	−0.47 (−0.72, −0.21)	*<*0.01			28
Total fat mass (kg)						
Overall effect size	397	−0.37 (−0.61, −0.13)			0.006	0
Subgrouped based on health status				0.54		
Apparently healthy	345	−0.39 (−0.71, −0.06)	0.03			0
Disease	52	−0.29 (−0.64, 0.06)	0.07			0
Subgrouped based on ITF type				0.57		
Inulin	272	−0.44 (−0.98, 0.1)	0.09			0
Oligofructose	108	−0.23 (−0.5, 0.03)	0.07			0
Oligofructose-enriched inulin	17	−0.7 (−2.49, 1.09)	NA (only 1 study)			NA (only 1 study)
Subgrouped based on dose				0.4		
Dose ≤ 10 g/d	245	−0.3 (−0.6, −0.007)	0.05			0
Dose > 10 g/d	152	−0.51 (−1.46, 0.43)	0.14			0
Subgrouped based on duration				0.91		
Duration ≤ 8 wk	102	−0.4 (−1.5, −0.71)	0.26			0
Duration > 8 wk	295	−0.36 (−0.66, −0.07)	0.02			0
Body fat percentage (%)						
Overall effect size	488	−0.35 (−0.8, 0.10)			0.11	75
Subgrouped based on health status				0.03		
Apparently healthy	368	−0.23 (−0.8, 0.33)	0.38			82
Disease	120	−0.9 (−1.47, −0.33)	0.02			0
Subgrouped based on ITF type				0.06		
Inulin	150	0.11 (−0.97, 1.19)	0.77			37
Oligofructose	103	−0.83 (−1.38, −0.28)	0.01			30
Oligofructose-enriched inulin	235	−0.27 (−2.05, 1.52)	0.67			71
Subgrouped based on dose				0.71		
Dose ≤ 10 g/d	272	−0.39 (−0.82, 0.05)	0.08			0
Dose > 10 g/d	216	−0.21 (−1.3, 0.88)	0.64			89
Subgrouped based on duration				0.04		
Duration ≤ 8 wk	180	0.11 (−0.91, 1.13)	0.79			55
Duration > 8 wk	308	−0.78 (−1.17, −0.39)	*<*0.01			49
Waist circumference (cm)						
Overall effect size	604	−1.03 (−1.69, −0.37)			0.004	51
Subgrouped based on health status				0.002		
Apparently healthy	427	−0.64 (−1.16, −0.11)	0.02			4
Disease	177	−2.47 (−3.78, −1.15)	*<*0.01			20
Subgrouped based on ITF type				0.03		
Inulin	419	−1.41 (−2.55, −0.27)	0.02			73
Oligofructose	129	−0.89 (−1.82, 0.05)	0.06			0
Oligofructose-enriched inulin	56	0.00 (−4.03, 4.03)	>0.05			0
Subgrouped based on dose				0.5		
Dose ≤ 10 g/d	368	−1.16 (−2.03, −0.29)	0.01			57
Dose > 10 g/d	236	−0.74 (−1.97, 0.5)	0.19			40
Subgrouped based on duration				0.99		
Duration ≤ 8 wk	310	−1.02 (−2.07, 0.04)	0.06			57
Duration > 8 wk	294	−1.02 (−2.01, −0.03)	0.05			42

Abbreviations: ITF, chicory inulin-type fructan; MD, mean difference; NA, not applicable.

### Secondary outcomes

#### BMI

**FIGURE 3:**
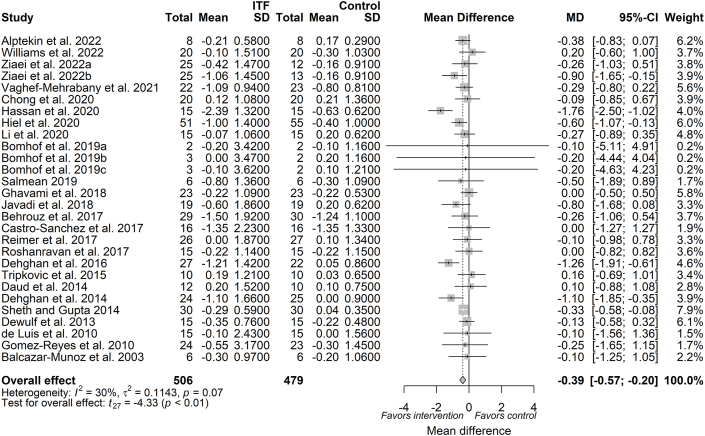
Forest plot of effects of inulin-type fructan (ITF) supplementation on BMI (kg/m^2^). Values were calculated as baseline-corrected mean difference (MD) and its corresponding 95% CI using random-effects model/inverse-variance method. To account for within-study and between-study variances, the Sidik–Jonkman estimator and Hartung–Knapp adjustment were applied into the model. Interstudy heterogeneity was quantified as *I*^2^. Significance level was set at *P* < 0.05.

As depicted in Figure 3, the pooled estimate from the random-effects model showed that a reduction in BMI (MD: −0.39 kg/m^2^; 95% CI: −0.57, −0.20; *P* = 0.0002) following chicory ITF supplementation was evident from 25 trials (available as 28 reports, *n* = 985 subjects). Between-study heterogeneity is considered as low to moderate and was not significant (*I*^2^: 30.2%; 95% CI: 0.00%, 56.2%; *P* = 0.07). A priori subgroup analysis showed that a significant reduction in BMI was observed at both doses of ≤10 g/d (MD: −0.48; 95% CI: −0.75, −0.22; *P* < 0.01) and >10 g/d (MD: −0.25; 95% CI: −0.43, −0.06; *P* < 0.01) (Supplemental Figure 7). Additionally, a priori subgroup analysis for duration is presented in Supplemental Figure 8. Similar to the body weight lowering effect, meta-regression showed that the magnitude of BMI reduction was not independently influenced by the dose (β_dose_: 0.017; *P* = 0.44) (Supplemental Figure 9) or duration of the intervention (β_duration_: 0.037; *P* = 0.22) (Supplemental Figure 10). A priori subgroup analyses for covariate ITF type and health status are presented in Supplemental Figures 11 and 12, respectively.

#### Total fat mass

**FIGURE 4:**
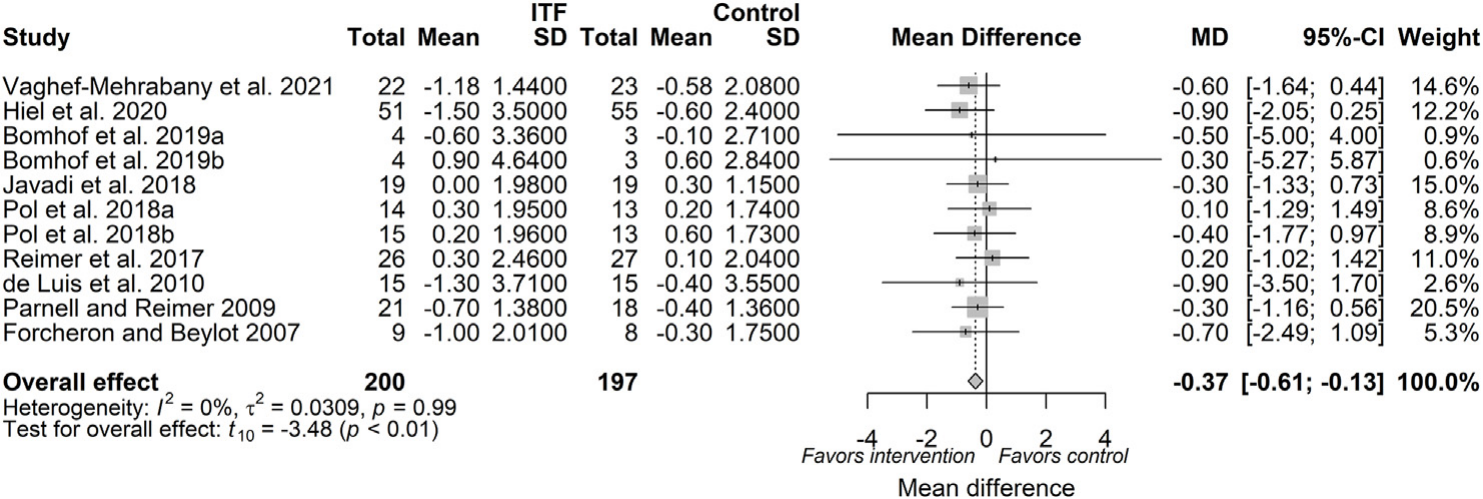
Forest plot of effects of inulin-type fructan (ITF) supplementation on total fat mass (kg). Values were calculated as baseline-corrected mean difference (MD) and its corresponding 95% CI using random-effects model/inverse-variance method. To account for within-study and between-study variances, the Sidik–Jonkman estimator and Hartung–Knapp adjustment were applied into the model. Interstudy heterogeneity was quantified as *I*^2^. Significance level was set at *P* < 0.05.

Total fat mass following ITF supplementation was measured in 9 trials (available as 11 reports, *n* = 397 subjects) with negligible heterogeneity (*I*^2^: 0%). The pooled estimate from the random-effects model showed that ITF supplementation significantly reduced total fat mass (MD: −0.37 kg; 95% CI: −0.61, −0.13; *P* = 0.01; *I*^2^: 0%), compared with placebo (Figure 4). A priori subgroup analyses for dose and duration are depicted in Supplemental Figures 13 and 14. Meta-regression indicated that both dose (β_dose_: −0.01; *P* = 0.07) (Supplemental Figure 15) and duration of intervention (β_duration_: −0.007; *P* = 0.09) (Supplemental Figure 16) showed a tendency to influence the magnitude of total fat mass reduction. A priori subgroup analysis based on ITF-type did not show that a certain type of ITF overrides the magnitude of effect on total fat mass reduction (Table 3; Supplemental Figure 17). With respect to health status, the magnitude of total fat mass reduction was observed both in apparently healthy participants and those with a disease condition (Table 3; Supplemental Figure 18).

#### Body fat percentage

**FIGURE 5:**
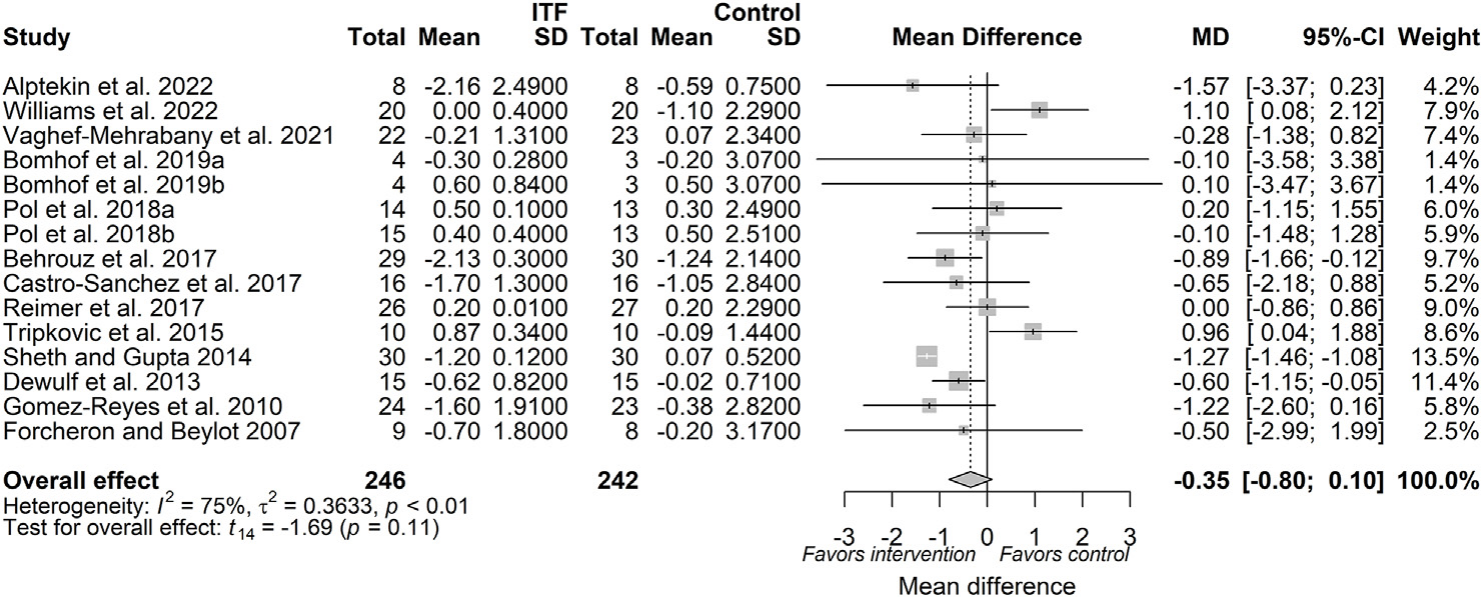
Forest plot of effects of inulin-type fructan (ITF) supplementation on body fat percentage (%). Values were calculated as baseline-corrected mean difference (MD) and its corresponding 95% CI using random-effects model/inverse-variance method. To account for within-study and between-study variances, the Sidik–Jonkman estimator and Hartung–Knapp adjustment were applied into the model. Interstudy heterogeneity was quantified as *I*^2^. Significance level was set at *P* < 0.05.

The analysis of effects of ITF supplementation on body fat percentage was performed on 13 trials (available as 15 reports, *n* = 488 subjects) with considerable heterogeneity (*I*^2^: 75%). The random-effects model showed that the pooled estimate after ITF supplementation did not significantly reduce body fat percentage (MD: −0.35%; 95% CI: −0.8, 0.1; *P* = 0.11), compared with control (Figure 5). No subgroup difference based on a priori analysis was found for dose (*P* = 0.71) (Supplemental Figure 19). However, a priori analysis based on intervention duration indicated that ITF supplementation longer than 8 wk exhibited a significant reduction in body fat percentage (MD: −0.78%; 95% CI: −1.17, −0.39; *P* < 0.01; *I*^2^: 49%) (Supplemental Figure 20). Meta-regression showed that neither dose (β_dose_: 0.03; *P* = 0.67) (Supplemental Figure 21) nor duration of the intervention (β_duration_: −0.06; *P* = 0.22) (Supplemental Figure 22) influenced body fat percentage. Furthermore, a priori subgroup analysis showed a significant body fat percentage reduction after ITF supplementation in subjects with a disease condition (MD: −0.83%; 95% CI: −1.38, −0.28; *P* = 0.01; *I*^2^: 30%) (Supplemental Figure 23) and with oligofructose as an ITF type (MD: −0.9%; 95% CI: −1.47, −0.33; *P* = 0.02; *I*^2^: 0%) (Supplemental Figure 24) with negligible heterogeneity (Table 3).

#### Waist circumference

**FIGURE 6:**
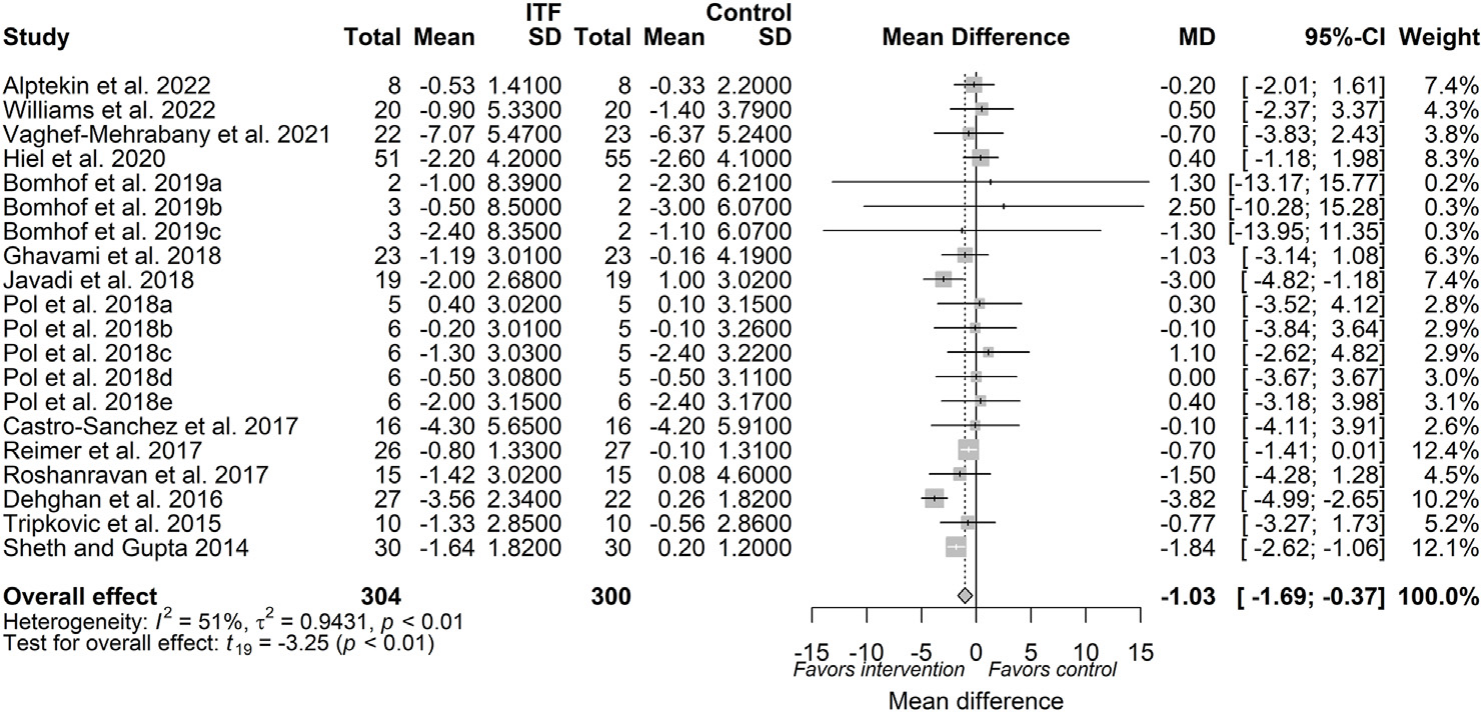
Forest plot of effects of inulin-type fructan (ITF) supplementation on waist circumference (cm). Values were calculated as baseline-corrected mean difference (MD) and its corresponding 95% CI using random-effects model/inverse-variance method. To account for within-study and between-study variances, the Sidik–Jonkman estimator and Hartung–Knapp adjustment were applied into the model. Interstudy heterogeneity was quantified as *I*^2^. Significance level was set at *P* < 0.05.

Waist circumference was measured in 14 trials (available as 20 reports, *n* = 604 subjects). The overall pooled estimate of waist circumference from the random-effects model showed a significant reduction (MD: −1.03 cm; 95% CI: −1.69, −0.37; *P* = 0.004, *I*^2^: 51%) following ITF supplementation, compared with control (Figure 6). A priori subgroup analysis based on dose and duration are depicted in Supplemental Figures 25 and 26, respectively. The continuous meta-regression analysis showed no evidence that dose (β_dose_: 0.001; *P* = 0.99) (Supplemental Figure 27) or duration (β_duration_: −0.06; *P* = 0.55) (Supplemental Figure 28) influenced the magnitude of waist circumference reduction. Supplementation with inulin showed greater effects on waist circumference reduction than other ITF types (oligofructose or oligofructose-enriched inulin) (Table 3; Supplemental Figure 29). Irrespective of participants health status, a significant reduction in waist circumference was observed in apparently healthy participants (MD: −0.64 cm; 95% CI: −1.16, −0.11; *P* = 0.02; *I*^2^: 4%) and in those with a disease condition (MD: −2.47 cm; 95% CI: −3.78, −1.15; *P* < 0.01; *I*^2^: 20%) (Table 3; Supplemental Figure 30).

### RoB assessment

**FIGURE 7:**
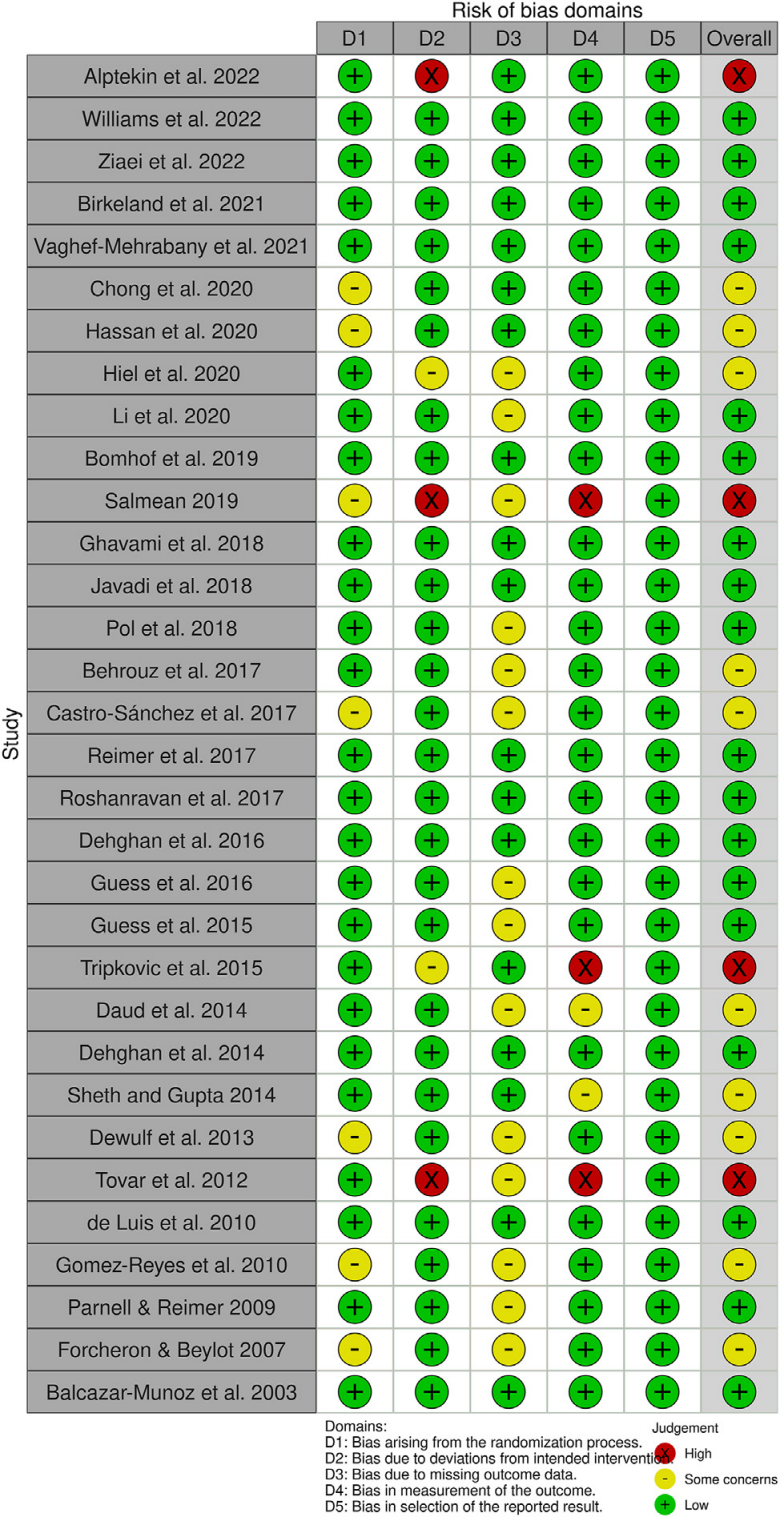
Distribution of risk of bias assessment across 5 domains and overall judgments in included studies.

**FIGURE 8:**
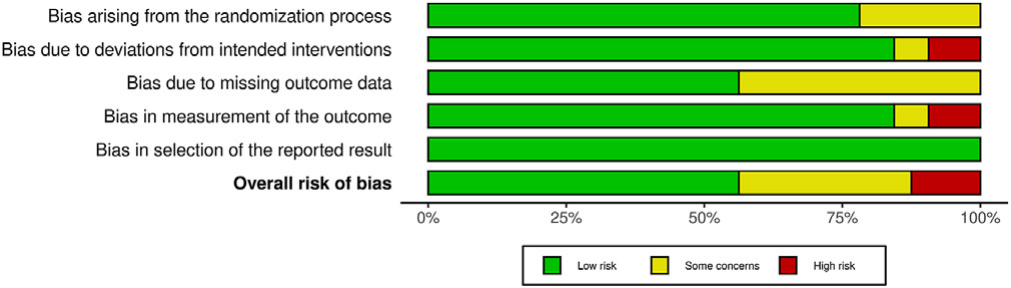
Summary assessment on risk of bias based on domain-level judgments.

Of the 32 included trials, 18 trials (56.25%) were assessed as having an overall low RoB, 10 trials (31.25%) had an unclear RoB, and 4 trials had a high RoB (12.5%). The RoB assessment across the 5 domains in each of the included studies is depicted in Figure 7, whereas the overall summary assessment of the RoB is presented in Figure 8.

### Reporting biases

Egger test indicated no publication bias for body weight (*P* = 0.12), BMI (*P* = 0.99), total fat mass (*P* = 0.93), and waist circumference (*P* = 0.18) (Supplemental Table 4). Funnel plots for body weight, BMI, total fat mass, and waist circumference are provided in Supplemental Figures 31–34, respectively. Although there seemed to be no asymmetry observed from inspection of the body fat percentage funnel plot (Supplemental Figure 35), the Egger test indicated significant publication bias (*P* = 0.01) (Supplemental Table 4). Given a potential publication bias in the body fat percentage outcome, trim-and-fill analysis was performed and subsequently provided an estimate of the number of missing studies with imputation of an additional 7 studies to balance the symmetry of the funnel plot (Supplemental Figure 36). Following trim-and-fill analysis, the adjusted overall MD for body fat percentage showed a significant reduction (MD: −1.04; 95% CI: −1.63, −0.46; *P* = 0.0013; *I*^2^: 82%) following ITF supplementation, compared with control.

## Discussion

### Summary of key findings

Dietary interventions that beneficially shift the gut microbiota hold promise for promoting health and reducing risk of developing noncommunicable diseases. Our findings contribute to growing evidence for prebiotics, particularly chicory-derived ITF, to positively influence components of weight management. Our systematic review and meta-analysis of 32 studies showed that the consumption of ITF led to significant reductions in body weight, BMI, fat mass, waist circumference, and, to a certain extent, body fat percentage. Subgroup analysis indicated that for all outcomes assessed, there was a benefit for both apparently healthy populations and those with a chronic disease. The duration of supplementation was shown to be important for a reduction in body weight with studies of ≥8 wk showing significantly larger reduction effects on body weight. For the secondary outcomes (BMI, fat mass, and waist circumference), there was no evidence that the type of ITF influenced the effect. However, oligofructose appears to consistently reduce body weight across the studies examined.

ITF have been shown to impact many physiologic processes that influence body weight. These include decreasing hunger and increasing subjective ratings of fullness and satiety [[54], [55]], enhancing the production and secretion of gut satiety hormones including PYY and less consistently GLP-1 [[39], [56]], and suppressing orexigenic hormones such as ghrelin [7]. In addition, ITF can alter the gut microbiota and especially enrich the gut microbial community in members that are typically reduced in the obese state. For example, Chanda and De [57] analyzed gut microbial metagenomic data from 3329 samples (obese, *n* = 1494; control, *n* = 1835) across 17 different countries to identify compositional and functional signatures of obesity [57]. They identified a lesser abundance of SCFAs producers (e.g., several *Alistipes* species) and a reduction in microbes that enhance the intestinal barrier (*Akkermansia muciniphila* and *Bifidobacterium longum*) in samples from participants with obesity compared with control participants [57]. Given that ITF have long been known to be bifidogenic [9] and more recently to also enrich for *A. muciniphila* [58], it is plausible that shifts in the gut microbial community play a role in the beneficial effects of ITF on body weight and metabolic outcomes.

Our random-effects model indicated that chicory ITF supplementation significantly reduced body weight (MD: −0.97 kg; 95% CI: −1.34, −0.59), compared with placebo. The magnitude of reduction we observed is similar to another meta-analysis that demonstrated a MD of −0.90 kg (95% CI −1.77, −0.02 kg) from interventional trials utilizing prebiotics in adults with BMI of ≥25 [11]. Surprisingly, the magnitude of body weight reduction in the present meta-analysis is even greater than other meta-analyses evaluating the effect of different sources of dietary fiber (MD: −0.37 kg; 95% CI: −0.63, −0.11 kg) [59], fiber-fortified food (MD: −0.31 kg; 95% CI: −0.59, −0.03 kg) [60], and dietary viscous fiber (MD: −0.33 kg; 95% CI: −0.51, −0.14 kg) [61]. The mechanisms underlying the greater weight management-related outcomes induced by prebiotic frutans could include their role as a selective substrate for beneficial gut microbiota and the metabolites they produce including SCFAs [9]. SCFAs can activate receptors on enteroendocrine cells to promote indirect signaling to the brain via the systemic circulation or vagal pathways by stimulating secretion of PYY and GLP-1 that regulate appetite and energy metabolism [10]. The ability of prebiotic ITF to modulate the secretion of gut hormones like GLP-1 and PYY leading to appetite and energy metabolism modulation has been extensively studied in animal models [[62], [63]] and human clinical trials [[7], [39], [45], [54], [56]]. Whether differences in SCFA production explain the greater magnitude of weight loss seen in our meta-analysis with ITF compared with other sources of fiber should be considered in the context of evidence that dietary fibers affect the composition of the gut microbiota and their metabolites, but all fibers do not produce equivocal increases in SCFA in healthy adults as discussed further.

A recent systematic review of 44 studies assessing the impact of dietary fiber intervention on SCFAs concluded that the effects of fiber intervention on SCFA profiles are highly dependent on the dose and the type and structure of the fiber [64]. Similar differences in SCFA production between fibers have been shown in other studies as well. For example, *in vitro* fermentation of an oat bran fiber with 28% β-glucan resulted in higher concentrations of propionate at 12 h whereas inulin resulted in higher acetate and butyrate at 12 h alongside a significant increase in the growth of *Collinsella* species [65]. This increase is noteworthy as 1 clinical study showed that inulin-type fructans increase *Collinsella* species abundance alongside increases in urinary hippurate levels [48]. It is possible that in addition to the beneficial effects of SCFAs, increases in hippurate may also explain in part the greater reduction in body weight seen in our meta-analysis than other dietary fibers. Hippurate, a metabolite of fermentation processes in the gut, is found in lower concentrations in individuals with obesity compared with lean individuals and is also lower in people with diabetes than those without diabetes [[66], [67], [68]].

Other evidence for the nonuniformity of dietary fibers comes from a systematic review by Wanders et al. [69] showing that different dietary fibers affected subjective appetite, acute energy intake, long-term energy intake, and body weight differently. Indeed, in a mouse study, both inulin and β-glucan supplementation caused similar reductions in weight gain from a high-fat diet [70]. However, β-glucan reduced energy intake to a greater extent, whereas inulin had a significantly greater effect on limiting total adipose tissue content and adipocyte size [70]. Different levels and patterns of SCFA production in the mice could explain the differences. Although binding of SCFAs to free fatty acid receptor (FFAR) 2 reduces free fatty acid output in adipocytes [71], binding to FFAR3 in the sympathetic nervous system stimulates energy expenditure [72]. Therefore, it is possible that although inulin and other dietary fibers both produce SCFA, the magnitude of their effects on weight-related outcomes could be influenced by their specific SCFA production patterns causing differential activation of FFAR2 and FFAR3 in adipose tissue and appetite centers in the brain.

In our subgroup analysis based on duration, the effect size based on duration of >8 wk increased to an MD of −1.31 kg. This magnitude of reduction in body weight could be considered clinically meaningful. There is convincing evidence that 5% weight loss provides sufficient benefits to be considered clinically meaningful [73]. However, given the continuum of weight loss for which benefits may be seen, there is some evidence to suggest that lower percentages (2%–5%) of weight loss may be of benefit [74]. It is also important to consider the lower body weight and BMI demonstrated in this study in the context of weight regain and recidivism in obesity. In the weight-reduced state, individuals continue to experience increased hunger, reduced feelings of satiation, and persistent increases in ghrelin and decreases in PYY [3]. The ability of ITF to impact subjective appetite and gut hormones known to impact food intake could make them a promising co-therapy with other means of preventing weight regain (e.g., physical activity) [[7], [54], [56]].

Accumulation of fat in the abdominal region has been shown to pose a significant risk for several chronic diseases [75]. In our analysis, there was a significant reduction found in body fat mass and to a certain extent only in interventions longer than 8 wk for reductions in body fat percentage following ITF supplementation. Although our study is limited in that we do not have measures of abdominal fat from imaging modalities, we do report a significant reduction in waist circumference with ITF supplementation. Interestingly, the magnitude of waist circumference reduction was found to almost triple in participants with a diagnosed disease. It is possible that the greater metabolic benefit (i.e., reduced waist circumference) was linked to a more robust microbial response and/or greater dysbiosis and therefore greater capacity for improvement. This is interesting to consider in light of recent findings from Kennedy et al. [76] who compared different prebiotics using *in vitro* batch culture fermentation with samples from 3 patients with ulcerative colitis and 3 healthy controls. They showed that the patients with ulcerative colitis demonstrated a greater capacity for change in microbial counts with the same substrates than the healthy controls [76]. The bacteria that were particularly responsive in this regard included *Bifidobacterium*, *Faecalibacterium prausnitzii*, *Lactobacillus* spp, and the butyrate producing *Clostridium coccoides*—*Eubacterium rectale* group. The authors suggest that participants with greater microbial dysbiosis at baseline may have a greater potential for beneficial microbial growth when they consume prebiotics than healthy controls. This may translate into greater metabolic benefit.

The magnitude of waist circumference reduction in this meta-analysis is greater than other meta-analyses with viscous fiber (MD: −0.63 cm; 95% CI: −1.11, −0.16) [61] or fiber-fortified food (MD: −0.27 cm; 95% CI: −0.67, 0.13) [60]. Given that visceral adipose tissue is highly metabolically active and secretes proinflammatory cytokines [77], dietary strategies such as ITF supplementation could hold promise for cardiometabolic benefits. Indeed, a systematic review and meta-analysis of studies of prebiotics and substances with prebiotic properties showed an improvement in metabolic and inflammatory biomarkers associated with T2D [78]. In another systematic review, 14 of 29 prebiotic studies reported a significant decrease in ≥1 marker of systemic inflammation with meta-analysis indicating that prebiotics reduced the common inflammatory marker, C-reactive protein (standardized MD: −0.60; 95% CI: −0.98, −0.23) [79].

### Strengths and limitations

Our study has several strengths including the strict selection of studies that investigated products that meet the current definition of an accepted or proven prebiotic [6]. This is important as not all dietary fibers/ingredients that modify the gut microbiota are considered prebiotics. We also conducted subgroup analysis to identify if studies that enrolled participants with a chronic disease experienced similar effects to those who are healthy and/or otherwise healthy individuals with overweight or obesity. Finally, investigation of the dose, duration, and type of ITF supplementation add valuable insight for the practical and/or clinical use of chicory ITF supplements. This study also has some limitations. There was significant heterogeneity in the studies assessed for the outcomes of body weight and body fat percentage; however, all other secondary outcomes had negligible to low heterogeneity. To address potential bias, we performed both RoB assessment according to the revised Cochrane RoB tool and reporting bias by performing both Egger test and by examining the asymmetry of the funnel plot. We also address the results both without adjustment and with Duval and Tweedie trim-and-fill method adjustment for the body fat percentage outcome that had potential bias based on Egger test.

### Implications and clinical applicability of the findings

Findings from this review suggest that a median daily dose of 10 g for 12 wk can lead to a clinically meaningful 2% reduction in body weight. Additionally, using an algorithm developed by Wald et al. [80], we found that overall BMI reduction (MD: −0.39) following ITF supplementation in this meta-analysis could be translated into a 10% absolute reduction of T2D incidence and an even greater absolute risk reduction of T2D incidence (13%) with intervention longer than 8 wk. Waist circumference was also reduced by an average of −1.72 cm following ITF supplementation. It is important to note that a 1-cm increase in waist circumference has been associated with an 8% relative risk incidence of T2D in both males and females [81].

### Conclusion

In conclusion, this meta-analysis provides evidence for chicory ITF supplementation to support weight management. Given the high prevalence of overweight and obesity globally and the need to improve gut microbiota ecology within individuals, chicory ITF supplementation may be one strategy or co-therapy to assist with weight management.

## Author contributions

The authors’ responsibilities are as follows – YCZ, ST, RAR: conceived the study concept and design; YCZ, ST, RAR: performed literature search, data collection, data extraction, and quality assessment, independently; YCZ: performed statistical analysis of meta-analysis and meta-regression; YCZ, RAR: drafted the manuscript; and all authors: critically revised and approved the final version of the manuscript.

## Funding

This study was funded by the BENEO Institute, Obrigheim/Pfalz, Germany.

## Data availability

Data described in the manuscript will be made available upon reasonable request.

## Conflict of interest

YCZ and ST are employed by BENEO Institute, c/o BENEO GmbH, Obrigheim/Pfalz, Germany. BENEO is a company involved in production of food ingredients including prebiotic fructans and polyols. RAR has received consulting fees and speaker honoraria related to presentations on prebiotics.
