# Supplementary material for: The effects of chicory inulin-type fructans supplementation on weight management outcomes: systematic review, meta-analysis, and meta-regression of randomized controlled trials
Source: Am J Clin Nutr. 2024 Sep 21;120(5):1245–58. doi: 10.1016/j.ajcnut.2024.09.019 (PMC11600113; doi:10.1016/j.ajcnut.2024.09.019)
Supplement: multimedia component 1 [file mmc1.pdf]

# The effects of chicory inulin-type fructans supplementation on weight management outcomes: systematic review, meta-analysis and meta-regression of randomized controlled trials

Raylene A. Reimer[1,2,3,*], Stephan Theis[4], and Yoghatama Cindya Zanzer[4]

[1] Faculty of Kinesiology, University of Calgary, 2500 University Drive NW, Calgary, AB T2N 1N4, Canada

[2] Department of Biochemistry and Molecular Biology, Cumming School of Medicine, University of Calgary, 3300 Hospital Drive NW, Calgary, AB T2N 4N1, Canada

[3] Alberta Children's Hospital Research Institute, Heritage Medical Research Building, 3330 Hospital Drive NW, Calgary, AB T2N 4N1, Canada

[4] BENEO Institute c/o BENEO GmbH, Wormser Str. 11, 67283 Obrigheim/Pfalz, Germany

***** Correspondence: Dr. Raylene Reimer, e-mail: reimer@ucalgary.ca

***Online supporting material***

The effects of chicory inulin-type fructans supplementation on weight management aspects: systematic review, meta-analysis and meta-regression of randomized controlled trials.

Raylene A. Reimer, Stephan Theis, and Yoghatama Cindya Zanzer

# Supplemental Tables


| | |
|---|---|
| **Supplemental Table 1** | Search strategy. |
| **Supplemental Table 2** | Equations used for data transformation. |
| **Supplemental Table 3** | Excluded studies and reason(s) for exclusion. |
| **Supplemental Table 4** | Assessment of publication bias with the Egger's test. |

*Online supporting material*

The effects of chicory inulin-type fructans supplementation on weight management aspects: systematic review, meta-analysis and meta-regression of randomized controlled trials.

Raylene A. Reimer, Stephan Theis, and Yoghatama Cindya Zanzer

**Supplemental Table 1** Search strategy

| Database | Search terms |
|---|---|
| EMBASE | #1. 'body weight':ab,ti OR 'body mass index':ab,ti OR 'BMI':ab,ti OR 'fat mass':ab,ti OR 'body fat*':ab,ti OR 'body fat mass':ab,ti OR 'fat percent*':ab,ti OR 'waist circumference':ab,ti OR 'weight control':ab,ti OR 'weight loss':ab,ti OR 'weight reduc*':ab,ti OR 'healthy':ab,ti OR 'overweight':ab,ti OR 'obes*':ab,ti OR 'prediabet*':ab,ti OR 'metabolic syndrome':ab,ti OR 'nafld':ab,ti OR 'nash':ab,ti OR 'randomi*ed':ab,ti OR 'trial':ab,ti OR 'intervention':ab,ti OR 'randomi*ed controlled trial':ab,ti OR 'clinical trial':ab,ti OR 'controlled trial':ab,ti<br>#2. 'chicory'/exp OR 'inulin'/exp OR 'inulin-type fructan*':ab,ti OR 'itf':ab,ti OR 'fructan*':ab,ti OR 'fos':ab,ti OR 'fructooligosaccharide*':ab,ti OR 'fructo-oligosacch*':ab,ti OR 'fructose cont* oligosaccharide*':ab,ti OR 'oligofructose'/exp OR 'oligo-fructose':ab,ti OR 'prebiot*':ab,ti<br>#3. #1 AND #2<br>#4. #3 AND [humans]/lim AND [clinical study]/lim |
| Medline (PubMed) | #1. 'body weight'[tiab] OR 'BMI'[tiab] OR 'body mass index'[tiab] OR 'fat mass'[tiab] OR 'body fat*'[tiab] OR 'body fat mass'[tiab] OR 'fat percent*'[tiab] OR 'waist circumference'[tiab] OR 'weight control'[tiab] OR 'weight loss'[tiab] OR 'weight reduc*'[tiab] OR 'healthy'[tiab] OR 'overweight'[tiab] OR 'obes*'[tiab] OR 'prediabet*'[tiab] OR 'metabolic syndrome'[tiab] OR 'NAFLD'[tiab] OR 'NASH'[tiab] OR 'controlled trial'[tiab] OR 'clinical trial'[tiab] OR 'randomi*ed controlled trial'[tiab] OR 'randomi*ed'[tiab] OR 'trial'[tiab] OR 'intervention'[tiab]<br>#2. 'chicory'[mh] OR 'inulin'[mh] OR 'fructan*'[tiab] OR 'FOS'[tiab] OR 'fructooligosaccharide*'[tiab] OR 'fructo-oligosacch*'[tiab] OR 'fructose cont* oligosaccharide*'[tiab] OR 'inulin-type fructan*'[tiab] OR 'ITF*'[tiab] OR 'oligofructose'[tiab] OR 'oligo-fructose'[tiab] OR 'prebiot*'[tiab]<br>#3. #1 AND #2<br>#4. #3 AND [humans]/lim AND [clinical study]/lim |
| Cochrane Library | #1. "body weight" OR "BMI" OR "body mass index" OR "fat mass" OR "body fat*" OR "body fat mass" OR "fat percent*" OR "waist circumference" OR "weight control" OR "weight loss" OR "weight reduc*" OR "healthy" OR "overweight" OR "obes*" OR "prediabet*" OR "metabolic syndrome" OR "NAFLD" OR "NASH" OR "controlled trial" OR "clinical trial" OR "randomi*ed controlled trial" OR "randomi*ed" OR "trial" OR "intervention"<br>#2. "chicory" OR "inulin" OR "fructan*" OR "FOS" OR "fructooligosaccharide*" OR "fructo-oligosacch*" OR "fructose cont* oligosaccharide*" OR "inulin-type fructan*" OR "ITF*" OR "oligofructose" OR "oligo-fructose" OR "prebiot*"<br>#3. #1 AND #2 |

*Abbreviations:* BMI, body mass index; CI, confidence interval; FOS, fructooligosaccharides; ITF, inulin-type fructans; NAFLD, non-alcoholic fatty liver disease; NASH, non-alcoholic steatohepatitis.

***Online supporting material***

The effects of chicory inulin-type fructans supplementation on weight management aspects: systematic review, meta-analysis and meta-regression of randomized controlled trials.

Raylene A. Reimer, Stephan Theis, and Yoghatama Cindya Zanzer

**Supplemental Table 2** Equations used for data transformation

| Equations | | Description | Reference |
|---|---|---|---|
| $SD = SEM \times \sqrt{n}$ | (1) | Equation (1) is used to obtain standard deviation (*SD*) from mean and standard error of the mean (*SEM*). | Cochrane Handbook (1) |
| $SD = \sqrt{n} \times \frac{(upper\ limit - lower\ limit)}{3.92}$ | (2) | Equation (2) is used to estimate *SD* from upper limit and lower limit of 95% confidence interval (*CI*) where the sample size is large. For large sample size ($n > 100$), the formula used for most reported 95% *CI* is using divisor 3.92. However, in any case where 90% *CI* or 99% *CI* are reported, the divisor 3.92 should be replaced by 3.29 or 5.15, respectively. | Cochrane Handbook (1) |
| $SD = \sqrt{n} \times \frac{(upper\ limit - lower\ limit)}{adjusted\ t.value}$ | (3) | Equation (3) is used to estimate *SD* from upper limit and lower limit of 95% *CI* where the sample size is small. For small sample size ($n \leq 60$), the divisor (3.95) must be adjusted and calculated a value from a t-distribution. For example, the *t*-statistic for a 95% *CI* from a sample size of 25 is 2.0639, then the divisor (3.92) should be replaced into 2 × 2.0639 = 4.128. | Cochrane Handbook (1) |
| $SD \approx \frac{q_3 - q_1}{2\Phi^{-1}\left(\frac{0.75n - 0.125}{n + 0.25}\right)}$ | (4) | Estimated *SD* is calculated using equation (4) where first quartile ($q_1$), third quartile ($q_3$) and sample size (*n*) are known; and $2\Phi–1(z)$ is the inverse function of $\Phi(z)$. | Wan *et al.* (2)<br>Cochrane Handbook (1) |
| $SD \approx \frac{b - a}{2\Phi^{-1}\left(\frac{n - 0.375}{n + 0.25}\right)}$ | (5) | Estimated *SD* is calculated using equation (5) where minimum value of range (*a*), maximum value of range (*b*) and sample size (*n*) | Wan *et al.* (2)<br>Cochrane Handbook (1) |

| Equations | | Description | Reference |
|---|---|---|---|
| | | are known; and Φ–1(*z*) is the inverse function of Φ(*z*). | |
| $SD \approx \frac{b-a}{4\Phi^{-1}\left(\frac{n-0.375}{n+0.25}\right)} + \frac{q_3-q_1}{4\Phi^{-1}\left(\frac{0.75n-0.125}{n+0.25}\right)}$ | (6) | Estimated *SD* is calculated using equation (6) where minimum value of range (*a*), maximum value of range (*b*), first quartile ($q_1$), third quartile ($q_3$) and sample size (*n*) are known; and Φ–1(*z*) is the inverse function of Φ(*z*). | Wan *et al.* (2) Cochrane Handbook (1) |
| $\bar{x} \approx \frac{a+2m+b}{4}$ | (7) | Equation (7) is used to estimate mean where median (*m*), minimum value of range (*a*) and maximum value of range (*b*) are known; and where sample size is small (*n* ≤ 25). For sample sizes greater than 25, the sample's median considered as the best estimate of its mean. | Hozo *et al.* (3) Cochrane Handbook (1) |
| $\bar{x} \approx \frac{q_1+m+q_3}{3}$ | (8) | Equation (8) is used to estimate mean where median (*m*), first quartile ($q_1$), third quartile ($q_3$) are known. | Cochrane Handbook (1) |

*Abbreviations:* CI, confidence interval; q1, first quartile; q3, third quartile; SD, standard deviation; SEM, standard error of the mean

# References

1. Higgins JPT, Thomas J, Chandler J, Cumpston M, Li T, Page MJ, Welch VA, eds. Cochrane Handbook for Systematic Reviews of Interventions. 2nd Edition. John Wiley & Sons: Chichester (UK).
2. Wan X, Wang W, Liu J, Tong T. Estimating the sample mean and standard deviation from the sample size, median, range and/or interquartile range. BMC Med Res Methodol 2014; 14:135.
3. Hozo SP, Djulbegovic B, Hozo I. Estimating the mean and variance from the median, range, and the size of a sample. BMC Med Res Methodol 2005; 5:13.

Raylene A. Reimer, Stephan Theis, and Yoghatama Cindya Zanzer

**Supplemental Table 3** Excluded studies and reason(s) for exclusion

| Study | Reason(s) for exclusion |
|---|---|
| Rodriguez *et al.* 2022 (1) | Weight management outcome parameters (body weight, BMI, fat mass, waist, waist/hip ratio, visceral fat) from the study population were integrated as part of larger cohort (Food4Gut Project) and reported in another paper (Hiel *et al.* 2020). |
| Calikoglu *et al.* 2021 (2) | Subjects underwent Roux-en-Y gastric bypass prior to oligofructose-enriched inulin. The RYGB procedure might have confounded the weight management outcome parameters. |
| Leyrolle *et al.* 2021 (3) | Weight management outcome parameters (body weight, BMI, fat mass, waist, waist/hip ratio, visceral fat) from the study population were integrated as part of larger cohort (Food4Gut Project) and reported in another paper (Hiel *et al.* 2020). |
| Nachit *et al.* 2021 (4) | Weight management outcome parameters (body weight, BMI, fat mass, waist, waist/hip ratio, visceral fat) from the study population were integrated as part of larger cohort (Food4Gut Project) and reported in another paper (Hiel *et al.* 2020). |
| Neyrinck *et al.* 2021 (5) | Weight management outcome parameters (body weight, BMI, fat mass, waist, waist/hip ratio, visceral fat) from the study population were integrated as part of larger cohort (Food4Gut Project) and reported in another paper (Hiel *et al.* 2020). |
| Zhuravlyova *et al.* 2021 (6) | The study results were presented/published in abstract form. Reduction in body weight outcome was only reported in percentage without mentioning the baseline value when participant first enrolled. |
| Behrouz *et al.* 2020 (7) | Weight management outcome parameters (body weight, BMI, body fat percentage, waist circumference, waist/hip ratio) from the study population were already reported in other paper (Behrouz *et al.* 2017) (8). |
| Hess *et al.* 2020 (9) | A mixture of inulin and resistant starch was used as an intervention. |
| Becerril-Alarcón *et al.* 2019 (10) | The source of oligofructose/inulin was not chicory root. |
| Medina-Vera *et al.* 2019 (11) | A mixture of inulin and other food materials (dehydrated nopal, chia seeds, and soy protein) was used as an intervention, while |

| Study | Reason(s) for exclusion |
|---|---|
| | mixture of calcium caseinate and maltodextrin was used as a control. |
| Uebelhack *et al.* 2019 (12) | A mixture of inulin and dehydrated okra powder was used as an intervention. |
| Cai *et al.* 2018 (13) | A mixture of inulin and resistant dextrin was used as an intervention. |
| Aliasgharzadeh *et al.* 2015 (14) | Changes from baseline on weight management outcome parameters (body weight, BMI) were numerically reported in active intervention but not in control. |
| Ramnani *et al.* 2015 (15) | The source of oligofructose/inulin was not chicory root. |
| Kellow *et al.* 2014 (16) | The paper is a study protocol. |
| Kaminskas *et al.* 2013 (17) | The study had no control intervention. |
| Montano-Hernandez *et al.* 2013 (18) | The study results were presented/published as an abstract form. The study had no control intervention. |
| Bonsu and Johnson 2012 (19) | Weight management outcome parameters (body weight, BMI, waist circumference) were reported only at baseline. |
| de Luis *et al.* 2011 (20) | In addition to oligofructose-enriched inulin, alpha linoleic was also added in a significant amount to the intervention cookies. It might confound the outcome parameters as no/trace amount was found in control cookies. |
| Lyon *et al.* 2011 (21) | No appropriate placebo control was used in the study. Mixture of dietary fiber (α-D-glucurono-α-D-manno-β-D-manno-β-D-gluco), (α-L-gulurono-β-D-mannurono), β-D-gluco-β-D-mannan) was tested in comparison to inulin. |
| Cicek *et al.* 2009 (22) | A mixture of inulin and polydextrose was used as an intervention. |
| Genta *et al.* 2009 (23) | The source of oligofructose/inulin was not chicory root. |
| Giacco *et al.* 2004 (24) | The study used scFOS derived from sucrose as an intervention. |
| Luo *et al.* 2000 (25) | The study used scFOS derived from sucrose as an intervention. |
| Alles *et al.* 1999 (26) | Weight management outcome parameters (body weight, BMI) were reported only at the end of intervention, but at baseline. |
| Jackson *et al.* 1999 (27) | Weight management outcome parameters (BMI) were reported only at baseline. |

*Online supporting material*

The effects of chicory inulin-type fructans supplementation on weight management aspects: systematic review, meta-analysis and meta-regression of randomized controlled trials.

Raylene A. Reimer, Stephan Theis, and Yoghatama Cindya Zanzer

| Study | Reason(s) for exclusion |
|---|---|
| Davidson *et al.* 1998 (28) | Weight management outcome parameters (body weight) were reported only at baseline. |
| Hidaka *et al.* 1991 (29) | The study used scFOS derived from sucrose as an intervention. |
| Yamashita *et al.* 1984 (30) | The study used scFOS derived from sucrose as an intervention. |

*Abbreviations:* BMI, body mass index; scFOS, sucrose-derived short chain fructooligosaccharides

## References

1. Rodriguez J, Neyrinck AM, van Kerckhoven M, Gianfrancesco MA, Renguet E, Bertrand L, Cani PD, Lanthier N, Cnop M, Paquot N, et al. Physical activity enhances the improvement of body mass index and metabolism by inulin: a multicenter randomized placebo-controlled trial performed in obese individuals. BMC Med 2022; 20:110.
2. Calikoglu F, Barbaros U, Uzum AK, Tutuncu Y, Satman I. The metabolic effects of pre-probiotic supplementation after Roux-en-Y gastric bypass (RYGB) surgery: a prospective, randomized controlled study. Obes Surg 2021; 31:215–23.
3. Leyrolle Q, Cserjesi R, D G H Mulders M, Zamariola G, Hiel S, Gianfrancesco MA, Portheault D, Amadieu C, Bindels LB, Leclercq S, et al. Prebiotic effect on mood in obese patients is determined by the initial gut microbiota composition: a randomized, controlled trial. Brain Behav Immun 2021; 94:289–98.
4. Nachit M, Lanthier N, Rodriguez J, Neyrinck AM, Cani PD, Bindels LB, Hiel S, Pachikian BD, Trefois P, Thissen J-P, et al. A dynamic association between myosteatosis and liver stiffness: results from a prospective interventional study in obese patients. JHEP Rep 2021; 3:100323.
5. Neyrinck AM, Rodriguez J, Zhang Z, Seethaler B, Sánchez CR, Roumain M, Hiel S, Bindels LB, Cani PD, Paquot N, et al. Prebiotic dietary fibre intervention improves fecal markers related to inflammation in obese patients: results from the Food4Gut randomized placebo-controlled trial. Eur J Nutr 2021; 60:3159–70.
6. Zhuravlyova L, Shekhovtsova Y. The influence of inulin on weight maintenance, appetite and body fat in patients with chronic pancreatitis and prediabetes. Pancreatology : official journal of the International Association of Pancreatology (IAP) ... [et al.] 2021; 21:S23-S23.
7. Behrouz V, Aryaeian N, Zahedi MJ, Jazayeri S. Effects of probiotic and prebiotic supplementation on metabolic parameters, liver aminotransferases, and systemic inflammation in nonalcoholic fatty liver disease: a randomized clinical trial. J Food Sci 2020; 85:3611–7.
8. Behrouz V, Jazayeri S, Aryaeian N, Zahedi MJ, Hosseini F. Effects of probiotic and prebiotic supplementation on leptin, adiponectin, and glycemic parameters in non-alcoholic fatty liver disease: a randomized clinical trial. Middle East J Dig Dis 2017; 9:150–7.
9. Hess AL, Benítez-Páez A, Blædel T, Larsen LH, Iglesias JR, Madera C, Sanz Y, Larsen TM. The effect of inulin and resistant maltodextrin on weight loss during energy restriction: a randomised, placebo-controlled, double-blinded intervention. Eur J Nutr 2020; 59:2507–24.

10. Becerril-Alarcón Y, Campos-Gómez S, Valdez-Andrade JJ, Campos-Gómez KA, Reyes-Barretero DY, Benítez-Arciniega AD, Valdés-Ramos R, Soto-Piña AE. Inulin supplementation reduces systolic blood pressure in women with breast cancer undergoing neoadjuvant chemotherapy. Cardiovasc Ther 2019; 2019:5707150.
11. Medina-Vera I, Sanchez-Tapia M, Noriega-López L, Granados-Portillo O, Guevara-Cruz M, Flores-López A, Avila-Nava A, Fernández ML, Tovar AR, Torres N. A dietary intervention with functional foods reduces metabolic endotoxaemia and attenuates biochemical abnormalities by modifying faecal microbiota in people with type 2 diabetes. Diabetes Metab 2019; 45:122–31.
12. Uebelhack R, Bongartz U, Seibt S, Bothe G, Chong PW, Costa P de, Wszelaki N. Double-blind, randomized, three-armed, placebo-controlled, clinical investigation to evaluate the benefit and tolerability of two dosages of IQP-AE-103 in reducing body weight in overweight and moderately obese subjects. J Obes 2019; 2019:3412952.
13. Cai X, Yu H, Liu L, Lu T, Li J, Ji Y, Le Z, Bao L, Ma W, Xiao R, et al. Milk powder co-supplemented with inulin and resistant dextrin improves glycemic control and insulin resistance in elderly type 2 diabetes mellitus: a 12-week randomized, double-blind, placebo-controlled trial. Mol Nutr Food Res 2018; 62:e1800865.
14. Aliasgharzadeh A, Khalili M, Mirtaheri E, Pourghassem Gargari B, Tavakoli F, Abbasalizad Farhangi M, Babaei H, Dehghan P. A combination of prebiotic inulin and oligofructose improve some of cardiovascular disease risk factors in women with type 2 diabetes: a randomized controlled clinical trial. Adv Pharm Bull 2015; 5:507–14.
15. Ramnani P, Costabile A, Bustillo AGR, Gibson GR. A randomised, double- blind, cross-over study investigating the prebiotic effect of agave fructans in healthy human subjects. J Nutr Sci 2015; 4:e10.
16. Kellow NJ, Coughlan MT, Savige GS, Reid CM. Effect of dietary prebiotic supplementation on advanced glycation, insulin resistance and inflammatory biomarkers in adults with pre-diabetes: a study protocol for a double-blind placebo-controlled randomised crossover clinical trial. BMC Endocr Disord 2014; 14:55.
17. Kaminskas A, Abaravi\vcius JA, Jablonskienė V, Valiūnienė J, Bagdonaite L, Andrikonytė J, Hendrixson V, Sekmokienė D. Quality of yoghurt enriched by inulinand its influence on human metabolic syndrome. VETERINARIJA IR ZOOTECHNIKA (Vet Med Zoot); 64:23–8.
18. Montano-Hernandez P, Lilia Castillo Martinez L, Orea-Tejeda A, Macias-Barata T, Vazquez-Duran M, Delgado-Perez DA, Marquez-Zepeda B, Tellez-Olvera LG, Lopez-Rodriguez Y. P478 Effects of consumption a bread contains agave or chicory inulin on lipids and blood pressure in patients with cardiovascular risk factors. European Journal of Preventive Cardiology 2013; 20:S84.
19. Bonsu NK, Johnson S. Effects of inulin fibre supplementation on serum glucose and lipid concentration in patients with type 2 diabetes. International Journal of Diabetes and Metabolism 2012; 20:80–6.
20. Luis DA de, La Fuente B de, Izaola O, Conde R, Gutiérrez S, Morillo M, Teba Torres C. Double blind randomized clinical trial controlled by placebo with an alpha linoleic acid and prebiotic enriched cookie on risk cardiovascular factor in obese patients. Nutr Hosp 2011; 26:827–33.
21. Lyon M, Wood S, Pelletier X, Donazzolo Y, Gahler R, Bellisle F. Effects of a 3-month supplementation with a novel soluble highly viscous polysaccharide on anthropometry and blood lipids in nondieting overweight or obese adults. J Hum Nutr Diet 2011; 24:351–9.

22. Cicek B, Arslan P, Kelestimur F. The effects of oligofructose and polydextrose on metabolic control parameters in type-2 diabetes. Pakistan Journal of Medical Sciences 2009; 25:573–8.
23. Genta S, Cabrera W, Habib N, Pons J, Carillo IM, Grau A, Sánchez S. Yacon syrup: beneficial effects on obesity and insulin resistance in humans. Clin Nutr 2009; 28:182–7.
24. Giacco R, Clemente G, Luongo D, Lasorella G, Fiume I, Brouns F, Bornet F, Patti L, Cipriano P, Rivellese AA, et al. Effects of short-chain fructo-oligosaccharides on glucose and lipid metabolism in mild hypercholesterolaemic individuals. Clin Nutr 2004; 23:331–40.
25. Luo J, van Yperselle M, Rizkalla SW, Rossi F, Bornet FR, Slama G. Chronic consumption of short-chain fructooligosaccharides does not affect basal hepatic glucose production or insulin resistance in type 2 diabetics. J Nutr 2000; 130:1572–7.
26. Alles MS, Roos NM de, Bakx JC, van de Lisdonk E, Zock PL, Hautvast GA. Consumption of fructooligosaccharides does not favorably affect blood glucose and serum lipid concentrations in patients with type 2 diabetes. Am J Clin Nutr 1999; 69:64–9.
27. Jackson KG, Taylor GR, Clohessy AM, Williams CM. The effect of the daily intake of inulin on fasting lipid, insulin and glucose concentrations in middle-aged men and women. Br J Nutr 1999; 82:23–30.
28. Davidson MH, Maki KC, Synecki C, Torri SA, Drennan KB. Effects of dietary inulin on serum lipids in men and women with hypercholesterolemia. Nutrition Research 1998; 18:503–17.
29. HIDAKA H, TASHIRO Y, EIDA T. Proliferation of Bifidobacteria by oligosaccharides and their useful effect on human health. Bifidobacteria Microflora 1991; 10:65–79.
30. Yamashita K, Kawai K, Itakura M. Effects of fructo-oligosaccharides on blood glucose and serum lipids in diabetic subjects. Nutrition Research 1984; 4:961–6.

The effects of chicory inulin-type fructans supplementation on weight management aspects: systematic review, meta-analysis and meta-regression of randomized controlled trials.

Raylene A. Reimer, Stephan Theis, and Yoghatama Cindya Zanzer

**Supplemental Table 4** Assessment of publication bias with the Egger's test

| Outcome parameters | Intercept | 95% confidence interval | t | *P* |
|---|---|---|---|---|
| Body weight (kg) | 0.91 | –0.2 to 2.03 | 1.61 | 0.12 |
| BMI (kg/m$^2$) | 0.003 | –0.9 to 0.91 | 0.007 | 0.99 |
| Total fat mass (kg) | –0.04 | –0.84 to 0.76 | –0.096 | 0.93 |
| Body fat percentage (%) | 1.72 | 0.6 to 2.84 | 2.99 | 0.01 |
| Waist circumference (cm) | 0.71 | –0.28 to 1.69 | 1.4 | 0.18 |

*Abbreviations:* BMI, body mass index; CI, confidence interval.

*Online supporting material*

The effects of chicory inulin-type fructans supplementation on weight management aspects: systematic review, meta-analysis and meta-regression of randomized controlled trials.

Raylene A. Reimer, Stephan Theis, and Yoghatama Cindya Zanzer

## Supplemental Figures

| | |
|---|---|
| **Supplemental Figure 1** | Forest plot baseline-corrected mean difference (MD) on body weight (kg) with subgroup analysis based on dose (dose > 10 g/d and ≤ 10 g/d) of inulin-type fructans supplementation. |
| **Supplemental Figure 2** | Meta-regression bubble-plot baseline-corrected mean difference (MD) on body weight (kg) by covariate dose (g/d) of inulin-type fructans supplementation. |
| **Supplemental Figure 3** | Forest plot baseline-corrected mean difference (MD) on body weight (kg) with subgroup analysis based on duration (duration > 8 wk and ≤ 8 wk) of inulin-type fructans supplementation. |
| **Supplemental Figure 4** | Meta-regression bubble-plot baseline-corrected mean difference (MD) on body weight (kg) by covariate duration (wk) of inulin-type fructans supplementation. |
| **Supplemental Figure 5** | Forest plot baseline-corrected mean difference (MD) on body weight (kg) with subgroup analysis based on type of inulin-type fructans (oligofructose, oligofructose-enriched inulin, and inulin) supplementation. |
| **Supplemental Figure 6** | Forest plot baseline-corrected mean difference (MD) on body weight (kg) with subgroup analysis based on subject health status (apparently healthy and diseased). |
| **Supplemental Figure 7** | Forest plot baseline-corrected mean difference (MD) on BMI ($kg/m^2$) with subgroup analysis based on dose (dose > 10 g/d and ≤ 10 g/d) of inulin-type fructans supplementation. |
| **Supplemental Figure 8** | Forest plot baseline-corrected mean difference (MD) on BMI ($kg/m^2$) with subgroup analysis based on duration (duration > 8 wk and ≤ 8 wk) of inulin-type fructans supplementation. |
| **Supplemental Figure 9** | Meta-regression bubble-plot baseline-corrected mean difference (MD) on BMI ($kg/m^2$) by covariate dose (g/d) of inulin-type fructans supplementation. |
| **Supplemental Figure 10** | Meta-regression bubble-plot baseline-corrected mean difference (MD) on BMI ($kg/m^2$) by covariate duration (wk) of inulin-type fructans supplementation. |

*Online supporting material*

The effects of chicory inulin-type fructans supplementation on weight management aspects: systematic review, meta-analysis and meta-regression of randomized controlled trials.

Raylene A. Reimer, Stephan Theis, and Yoghatama Cindya Zanzer

**Supplemental Figure 11** Forest plot baseline-corrected mean difference (MD) on BMI (kg/m$^2$) with subgroup analysis based on type of inulin-type fructans (oligofructose, oligofructose-enriched inulin, and inulin) supplementation.

**Supplemental Figure 12** Forest plot baseline-corrected mean difference (MD) on BMI (kg/m$^2$) with subgroup analysis based on subject health status (apparently healthy and diseased).

**Supplemental Figure 13** Forest plot baseline-corrected mean difference (MD) on total fat mass (kg) with subgroup analysis based on dose (dose > 10 g/d and ≤ 10 g/d) of inulin-type fructans supplementation.

**Supplemental Figure 14** Forest plot baseline-corrected mean difference (MD) on total fat mass (kg) with subgroup analysis based on duration (duration > 8 wk and ≤ 8 wk) of inulin-type fructans supplementation.

**Supplemental Figure 15** Meta-regression bubble-plot baseline-corrected mean difference (MD) on total fat mass (kg) by covariate dose (g/d) of inulin-type fructans supplementation.

**Supplemental Figure 16** Meta-regression bubble-plot baseline-corrected mean difference (MD) on total fat mass (kg) by covariate duration (wk) of inulin-type fructans supplementation.

**Supplemental Figure 17** Forest plot baseline-corrected mean difference (MD) on total fat mass (kg) with subgroup analysis type of inulin-type fructans (oligofructose, oligofructose-enriched inulin, and inulin) supplementation.

**Supplemental Figure 18** Forest plot baseline-corrected mean difference (MD) on total fat mass (kg) with subgroup analysis health status (apparently healthy and diseased).

**Supplemental Figure 19** Forest plot baseline-corrected mean difference (MD) on body fat percentage (%) with subgroup analysis based on dose (dose > 10 g/d and ≤ 10 g/d) of inulin-type fructans supplementation.

**Supplemental Figure 20** Forest plot baseline-corrected mean difference (MD) on body fat percentage (%) with subgroup analysis based on duration (duration > 8 wk and ≤ 8 wk) of inulin-type fructans supplementation.

**Supplemental Figure 21** Meta-regression bubble-plot baseline-corrected mean difference (MD) on body fat percentage (%) by covariate dose (g/d) of inulin-type fructans supplementation.

| | |
|---|---|
| **Supplemental Figure 22** | Meta-regression bubble-plot baseline-corrected mean difference (MD) on body fat percentage (%) by covariate duration (wk) of inulin-type fructans supplementation. |
| **Supplemental Figure 23** | Forest plot baseline-corrected mean difference (MD) on body fat percentage (%) with subgroup analysis health status (apparently healthy and diseased). |
| **Supplemental Figure 24** | Forest plot baseline-corrected mean difference (MD) on body fat percentage (%) with subgroup analysis type of inulin-type fructans (oligofructose, oligofructose-enriched inulin, and inulin) supplementation. |
| **Supplemental Figure 25** | Forest plot baseline-corrected mean difference (MD) on waist circumference (cm) with subgroup analysis based on dose (dose > 10 g/d and ≤ 10 g/d) of inulin-type fructans supplementation. |
| **Supplemental Figure 26** | Forest plot baseline-corrected mean difference (MD) on waist circumference (cm) with subgroup analysis based on duration (duration > 8 wk and ≤ 8 wk) of inulin-type fructans supplementation. |
| **Supplemental Figure 27** | Meta-regression bubble-plot baseline-corrected mean difference (MD) on waist circumference (cm) by covariate dose (g/d) of inulin-type fructans supplementation. |
| **Supplemental Figure 28** | Meta-regression bubble-plot baseline-corrected mean difference (MD) on waist circumference (cm) by covariate duration (wk) of inulin-type fructans supplementation. |
| **Supplemental Figure 29** | Forest plot baseline-corrected mean difference (MD) on waist circumference (cm) with subgroup analysis type of inulin-type fructans (oligofructose, oligofructose-enriched inulin, and inulin) supplementation. |
| **Supplemental Figure 30** | Forest plot baseline-corrected mean difference (MD) on waist circumference (cm) with subgroup analysis health status (apparently healthy and diseased). |
| **Supplemental Figure 31** | Funnel plot assessing publication bias for body weight (kg). |
| **Supplemental Figure 32** | Funnel plot assessing publication bias for BMI (kg/m$^2$). |
| **Supplemental Figure 33** | Funnel plot assessing publication bias for total fat mass (kg). |

***Online supporting material***

The effects of chicory inulin-type fructans supplementation on weight management aspects: systematic review, meta-analysis and meta-regression of randomized controlled trials.

Raylene A. Reimer, Stephan Theis, and Yoghatama Cindya Zanzer

| | |
|---|---|
| **Supplemental Figure 34** | Funnel plot assessing publication bias for waist circumference (cm). |
| **Supplemental Figure 35** | Funnel plot assessing publication bias for body fat percentage (%). |
| **Supplemental Figure 36** | Funnel plot following Trim-and-Fill analysis for body fat percentage (%) outcome parameter. |

***Online supporting material***

The effects of chicory inulin-type fructans supplementation on weight management aspects: systematic review, meta-analysis and meta-regression of randomized controlled trials.

Raylene A. Reimer, Stephan Theis, and Yoghatama Cindya Zanzer

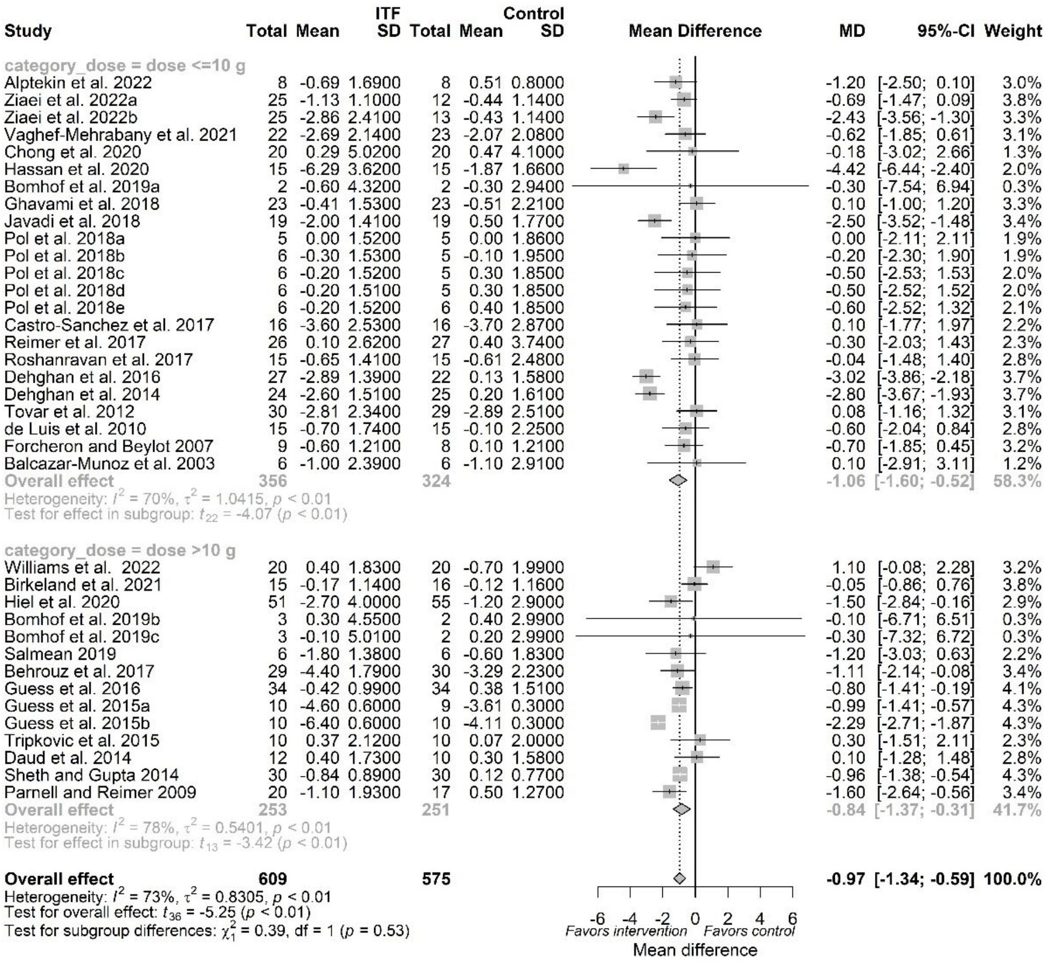


**Supplemental Figure 1** Forest plot baseline-corrected mean difference (MD) and corresponding 95% confidence interval (CI) on body weight (kg) with subgroup analysis based on dose (dose > 10 g/d and ≤ 10 g/d) of inulin-type fructans supplementation. The diamond represents the pooled effect estimate for the overall analysis. Effect size was calculated using random-effects model/inverse-variance method with Sidik-Jonkman estimator and Hartung-Knapp adjustment to account within and between studies variances. Interstudy heterogeneity was quantified as $I^2$. Significance level was set at $P < 0.05$. *Abbreviations:* CI, confidence interval; ITF, inulin-type fructans; MD, baseline-corrected mean difference; SD, standard deviation; total, total number of participants completed the study.

*Online supporting material*

The effects of chicory inulin-type fructans supplementation on weight management aspects: systematic review, meta-analysis and meta-regression of randomized controlled trials.

Raylene A. Reimer, Stephan Theis, and Yoghatama Cindya Zanzer

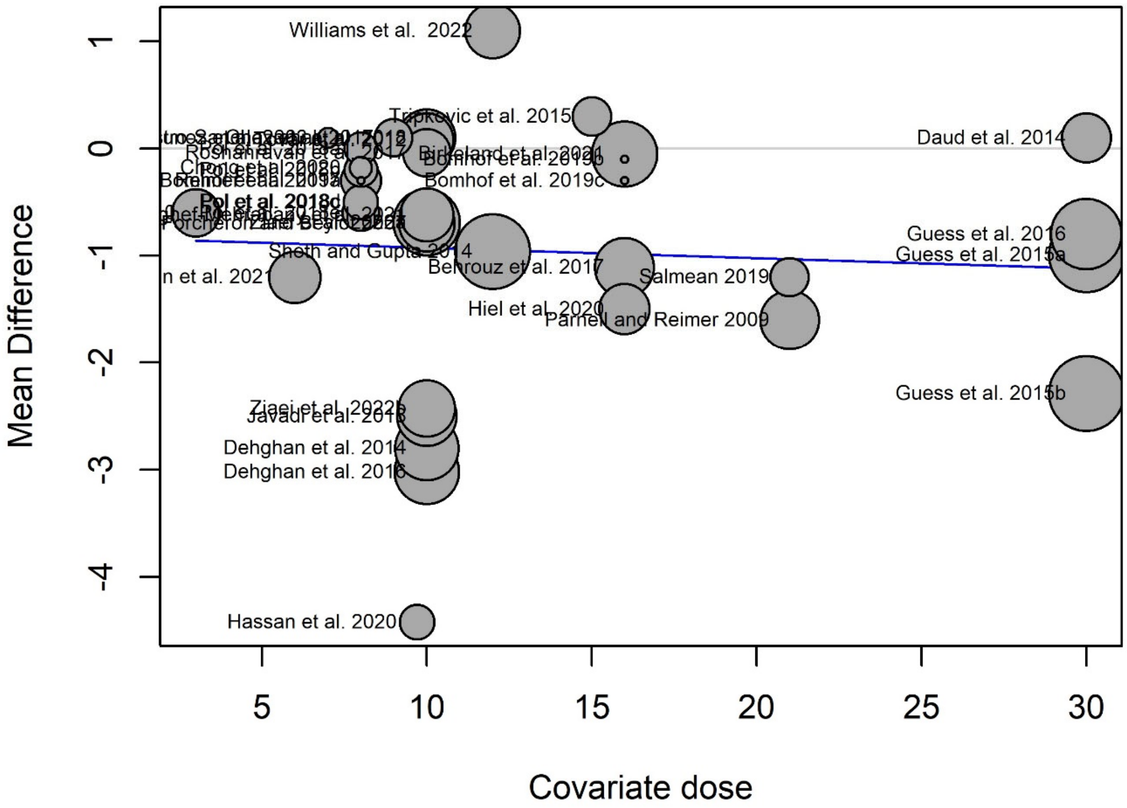


**Supplemental Figure 2** Meta-regression bubble-plot baseline-corrected mean difference (MD) on body weight (kg) by covariate dose (g/d) of inulin-type fructans supplementation. Circles represent each of the included studies in the meta-analysis, and the size of the circles corresponds to the inverse variance-weight of the body weight effect.

## *Online supporting material*

The effects of chicory inulin-type fructans supplementation on weight management aspects: systematic review, meta-analysis and meta-regression of randomized controlled trials.

Raylene A. Reimer, Stephan Theis, and Yoghatama Cindya Zanzer

| Study | ITF Total | ITF Mean | ITF SD | Control Total | Control Mean | Control SD | MD | 95%-CI | Weight |
|---|---|---|---|---|---|---|---|---|---|
| category_duration = duration <=8 wk | | | | | | | | | |
| Alptekin et al. 2022 | 8 | -0.69 | 1.6900 | 8 | 0.51 | 0.8000 | -1.20 | [-2.50; 0.10] | 3.0% |
| Williams et al. 2022 | 20 | 0.40 | 1.8300 | 20 | -0.70 | 1.9900 | 1.10 | [-0.08; 2.28] | 3.2% |
| Birkeland et al. 2021 | 15 | -0.17 | 1.1400 | 16 | -0.12 | 1.1600 | -0.05 | [-0.86; 0.76] | 3.8% |
| Vaghef-Mehrabany et al. 2021 | 22 | -2.69 | 2.1400 | 23 | -2.07 | 2.0800 | -0.62 | [-1.85; 0.61] | 3.1% |
| Salmean 2019 | 6 | -1.80 | 1.3800 | 6 | -0.60 | 1.8300 | -1.20 | [-3.03; 0.63] | 2.2% |
| Ghavami et al. 2018 | 23 | -0.41 | 1.5300 | 23 | -0.51 | 2.2100 | 0.10 | [-1.00; 1.20] | 3.3% |
| Pol et al. 2018a | 5 | 0.00 | 1.5200 | 5 | 0.00 | 1.8600 | 0.00 | [-2.11; 2.11] | 1.9% |
| Pol et al. 2018b | 6 | -0.30 | 1.5300 | 5 | -0.10 | 1.9500 | -0.20 | [-2.30; 1.90] | 1.9% |
| Pol et al. 2018c | 6 | -0.20 | 1.5200 | 5 | 0.30 | 1.8500 | -0.50 | [-2.53; 1.53] | 2.0% |
| Castro-Sanchez et al. 2017 | 16 | -3.60 | 2.5300 | 16 | -3.70 | 2.8700 | 0.10 | [-1.77; 1.97] | 2.2% |
| Roshanravan et al. 2017 | 15 | -0.65 | 1.4100 | 15 | -0.61 | 2.4800 | -0.04 | [-1.48; 1.40] | 2.8% |
| Dehghan et al. 2016 | 27 | -2.89 | 1.3900 | 22 | 0.13 | 1.5800 | -3.02 | [-3.86; -2.18] | 3.7% |
| Guess et al. 2016 | 34 | -0.42 | 0.9900 | 34 | 0.38 | 1.5100 | -0.80 | [-1.41; -0.19] | 4.1% |
| Tripkovic et al. 2015 | 10 | 0.37 | 2.1200 | 10 | 0.07 | 2.0000 | 0.30 | [-1.51; 2.11] | 2.3% |
| Daud et al. 2014 | 12 | 0.40 | 1.7300 | 10 | 0.30 | 1.5800 | 0.10 | [-1.28; 1.48] | 2.8% |
| Dehghan et al. 2014 | 24 | -2.60 | 1.5100 | 25 | 0.20 | 1.6100 | -2.80 | [-3.67; -1.93] | 3.7% |
| de Luis et al. 2010 | 15 | -0.70 | 1.7400 | 15 | -0.10 | 2.2500 | -0.60 | [-2.04; 0.84] | 2.8% |
| Balcazar-Munoz et al. 2003 | 6 | -1.00 | 2.3900 | 6 | -1.10 | 2.9100 | 0.10 | [-2.91; 3.11] | 1.2% |
| Overall effect | 270 | | | 264 | | | -0.63 | [-1.19; -0.06] | 49.8% |
| Heterogeneity: $I^2$ = 75%, $\tau^2$ = 0.8142, $p$ < 0.01 | | | | | | | | | |
| Test for effect in subgroup: $t_{17}$ = -2.35 ($p$ = 0.03) | | | | | | | | | |
| category_duration = duration >8 wk | | | | | | | | | |
| Ziaei et al. 2022a | 25 | -1.13 | 1.1000 | 12 | -0.44 | 1.1400 | -0.69 | [-1.47; 0.09] | 3.8% |
| Ziaei et al. 2022b | 25 | -2.86 | 2.4100 | 13 | -0.43 | 1.1400 | -2.43 | [-3.56; -1.30] | 3.3% |
| Chong et al. 2020 | 20 | 0.29 | 5.0200 | 20 | 0.47 | 4.1000 | -0.18 | [-3.02; 2.66] | 1.3% |
| Hassan et al. 2020 | 15 | -6.29 | 3.6200 | 15 | -1.87 | 1.6600 | -4.42 | [-6.44; -2.40] | 2.0% |
| Hiel et al. 2020 | 51 | -2.70 | 4.0000 | 55 | -1.20 | 2.9000 | -1.50 | [-2.84; -0.16] | 2.9% |
| Bomhof et al. 2019a | 2 | -0.60 | 4.3200 | 2 | -0.30 | 2.9400 | -0.30 | [-7.54; 6.94] | 0.3% |
| Bomhof et al. 2019b | 3 | 0.30 | 4.5500 | 2 | 0.40 | 2.9900 | -0.10 | [-6.71; 6.51] | 0.3% |
| Bomhof et al. 2019c | 3 | -0.10 | 5.0100 | 2 | 0.20 | 2.9900 | -0.30 | [-7.32; 6.72] | 0.3% |
| Javadi et al. 2018 | 19 | -2.00 | 1.4100 | 19 | 0.50 | 1.7700 | -2.50 | [-3.52; -1.48] | 3.4% |
| Pol et al. 2018d | 6 | -0.20 | 1.5100 | 5 | 0.30 | 1.8500 | -0.50 | [-2.52; 1.52] | 2.0% |
| Pol et al. 2018e | 6 | -0.20 | 1.5200 | 6 | 0.40 | 1.8500 | -0.60 | [-2.52; 1.32] | 2.1% |
| Behrouz et al. 2017 | 29 | -4.40 | 1.7900 | 30 | -3.29 | 2.2300 | -1.11 | [-2.14; -0.08] | 3.4% |
| Reimer et al. 2017 | 26 | 0.10 | 2.6200 | 27 | 0.40 | 3.7400 | -0.30 | [-2.03; 1.43] | 2.3% |
| Guess et al. 2015a | 10 | -4.60 | 0.6000 | 9 | -3.61 | 0.3000 | -0.99 | [-1.41; -0.57] | 4.3% |
| Guess et al. 2015b | 10 | -6.40 | 0.6000 | 10 | -4.11 | 0.3000 | -2.29 | [-2.71; -1.87] | 4.3% |
| Sheth and Gupta 2014 | 30 | -0.84 | 0.8900 | 30 | 0.12 | 0.7700 | -0.96 | [-1.38; -0.54] | 4.3% |
| Tovar et al. 2012 | 30 | -2.81 | 2.3400 | 29 | -2.89 | 2.5100 | 0.08 | [-1.16; 1.32] | 3.1% |
| Parnell and Reimer 2009 | 20 | -1.10 | 1.9300 | 17 | 0.50 | 1.2700 | -1.60 | [-2.64; -0.56] | 3.4% |
| Forcheron and Beylot 2007 | 9 | -0.60 | 1.2100 | 8 | 0.10 | 1.2100 | -0.70 | [-1.85; 0.45] | 3.2% |
| Overall effect | 339 | | | 311 | | | -1.31 | [-1.80; -0.82] | 50.2% |
| Heterogeneity: $I^2$ = 68%, $\tau^2$ = 0.7013, $p$ < 0.01 | | | | | | | | | |
| Test for effect in subgroup: $t_{18}$ = -5.62 ($p$ < 0.01) | | | | | | | | | |
| **Overall effect** | **609** | | | **575** | | | **-0.97** | **[-1.34; -0.59]** | **100.0%** |
| Heterogeneity: $I^2$ = 73%, $\tau^2$ = 0.8305, $p$ < 0.01 | | | | | | | | | |
| Test for overall effect: $t_{36}$ = -5.25 ($p$ < 0.01) | | | | | | | | | |
| Test for subgroup differences: $\chi^2_1$ = 3.68, df = 1 ($p$ = 0.06) | | | | | | | | | |

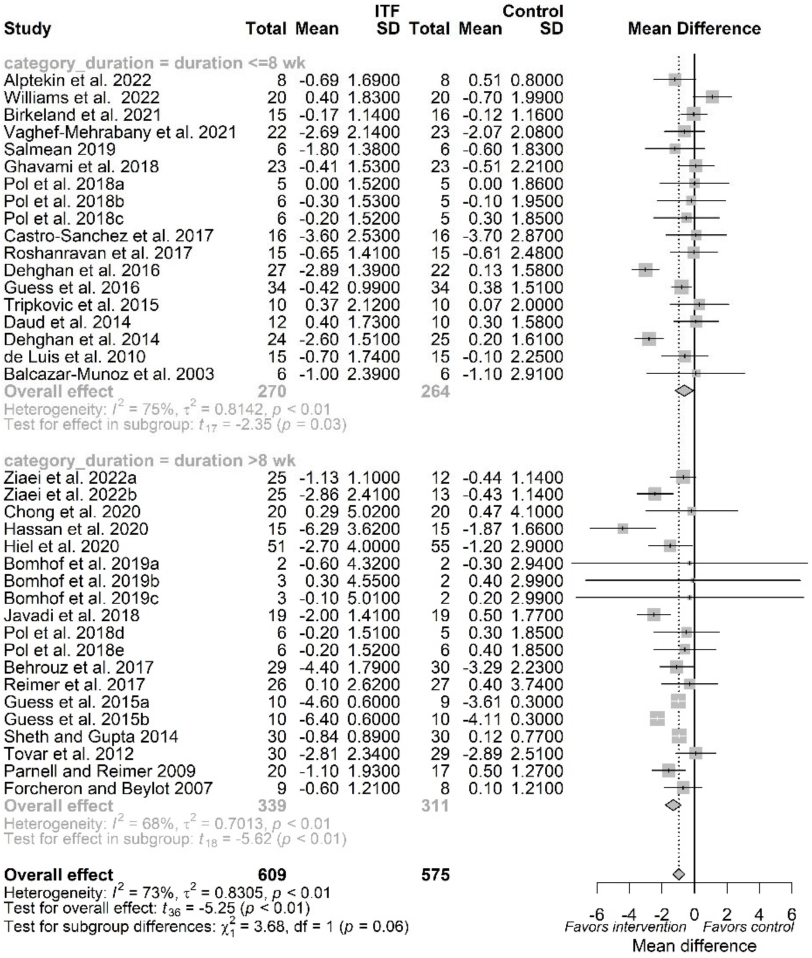


**Supplemental Figure 3** Forest plot baseline-corrected mean difference (MD) and corresponding 95% confidence interval (CI) on body weight (kg) with subgroup analysis based on duration (duration > 8 wk and ≤ 8 wk) of inulin-type fructans supplementation. The diamond represents the pooled effect estimate for the overall analysis. Effect size was calculated using random-effects model/inverse-variance method with Sidik-Jonkman estimator and Hartung-Knapp adjustment to account within and between studies variances. Interstudy heterogeneity was quantified as $I^2$. Significance level was set at $P < 0.05$. *Abbreviations:* CI, confidence interval; ITF, inulin-type fructans; MD, baseline-corrected mean difference; SD, standard deviation; total, total number of participants completed the study.

The effects of chicory inulin-type fructans supplementation on weight management aspects: systematic review, meta-analysis and meta-regression of randomized controlled trials.

Raylene A. Reimer, Stephan Theis, and Yoghatama Cindya Zanzer

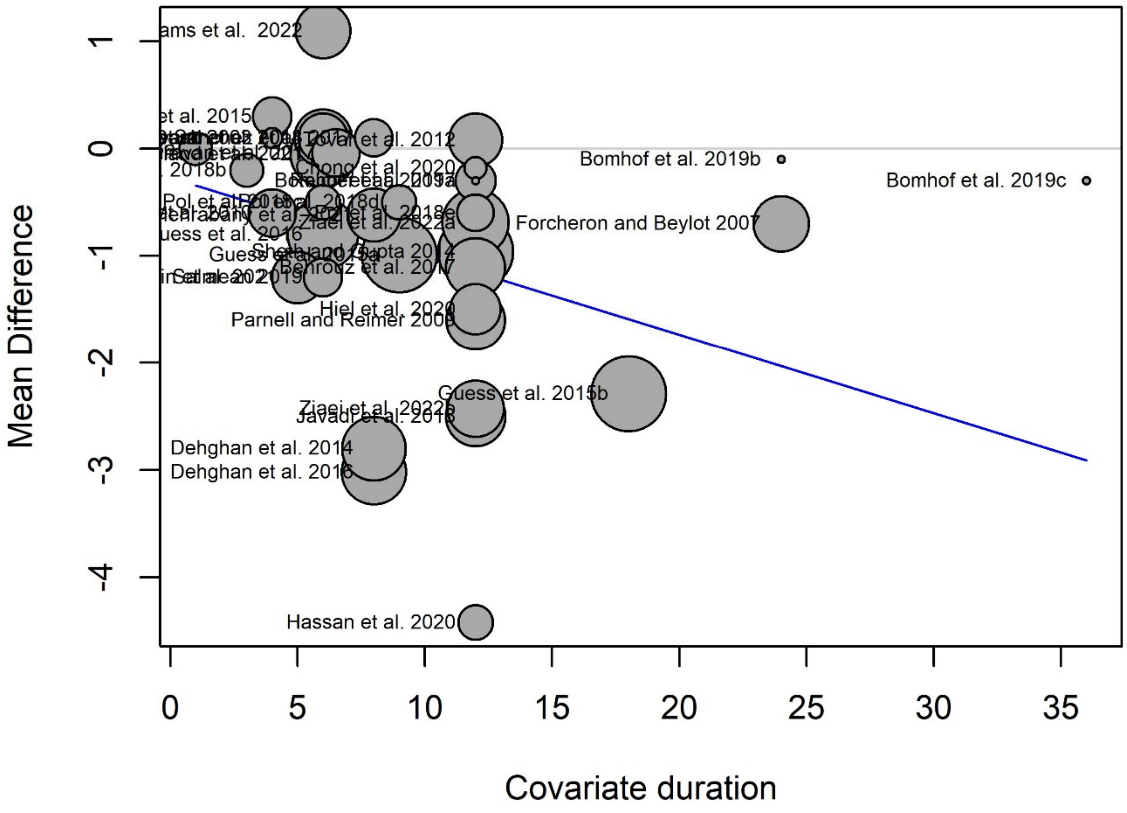


**Supplemental Figure 4** Meta-regression bubble-plot baseline-corrected mean difference (MD) on body weight (kg) by covariate duration (wk) of inulin-type fructans supplementation. Circles represent each of the included studies in the meta-analysis, and the size of the circles corresponds to the inverse variance-weight of the body weight effect.

## *Online supporting material*

The effects of chicory inulin-type fructans supplementation on weight management aspects: systematic review, meta-analysis and meta-regression of randomized controlled trials.

Raylene A. Reimer, Stephan Theis, and Yoghatama Cindya Zanzer

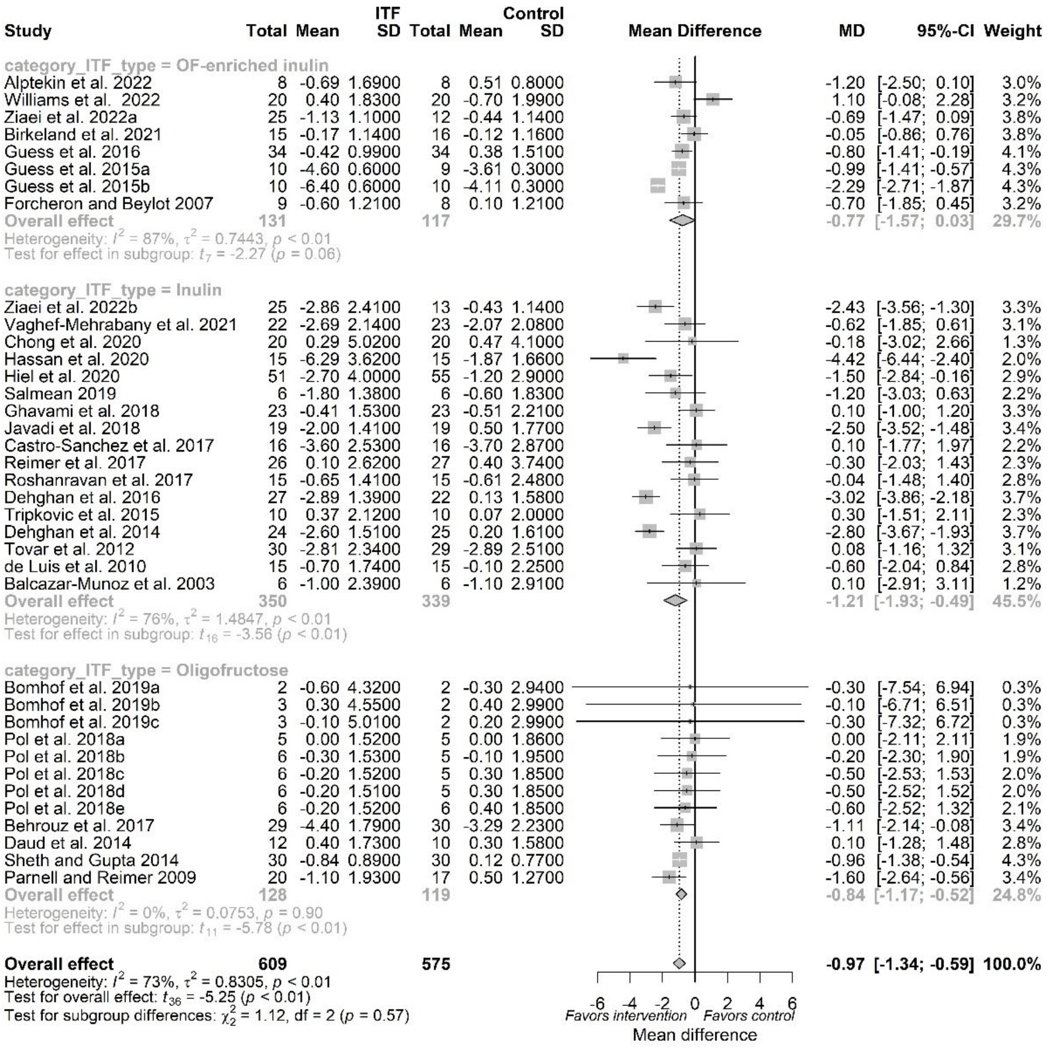


**Supplemental Figure 5** Forest plot baseline-corrected mean difference (MD) and corresponding 95% confidence interval (CI) on body weight (kg) with subgroup analysis based on type of inulin-type fructans (oligofructose, oligofructose-enriched inulin, and inulin) supplementation. The diamond represents the pooled effect estimate for the overall analysis. Effect size was calculated using random-effects model/inverse-variance method with Sidik-Jonkman estimator and Hartung-Knapp adjustment to account within and between studies variances. Interstudy heterogeneity was quantified as $I^2$. Significance level was set at $P < 0.05$. *Abbreviations:* CI, confidence interval; ITF, inulin-type fructans; MD, baseline-corrected mean difference; SD, standard deviation; total, total number of participants completed the study.

***Online supporting material***

The effects of chicory inulin-type fructans supplementation on weight management aspects: systematic review, meta-analysis and meta-regression of randomized controlled trials.

Raylene A. Reimer, Stephan Theis, and Yoghatama Cindya Zanzer

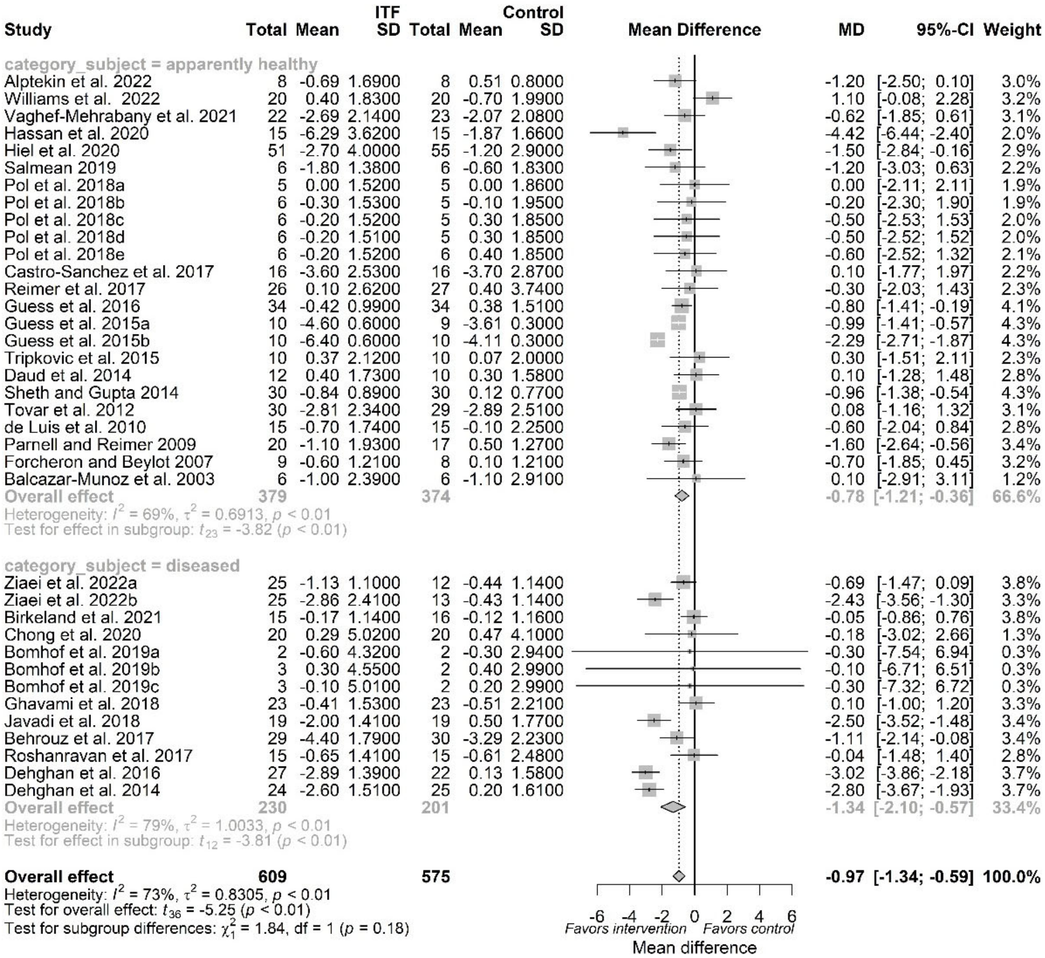


**Supplemental Figure 6** Forest plot baseline-corrected mean difference (MD) and corresponding 95% confidence interval (CI) on body weight (kg) with subgroup analysis based on subject health status (apparently healthy and diseased). The diamond represents the pooled effect estimate for the overall analysis. Effect size was calculated using random-effects model/inverse-variance method with Sidik-Jonkman estimator and Hartung-Knapp adjustment to account within and between studies variances. Interstudy heterogeneity was quantified as $I^2$. Significance level was set at $P < 0.05$. *Abbreviations:* CI, confidence interval; ITF, inulin-type fructans; MD, baseline-corrected mean difference; SD, standard deviation; total, total number of participants completed the study.

## *Online supporting material*

The effects of chicory inulin-type fructans supplementation on weight management aspects: systematic review, meta-analysis and meta-regression of randomized controlled trials.

Raylene A. Reimer, Stephan Theis, and Yoghatama Cindya Zanzer

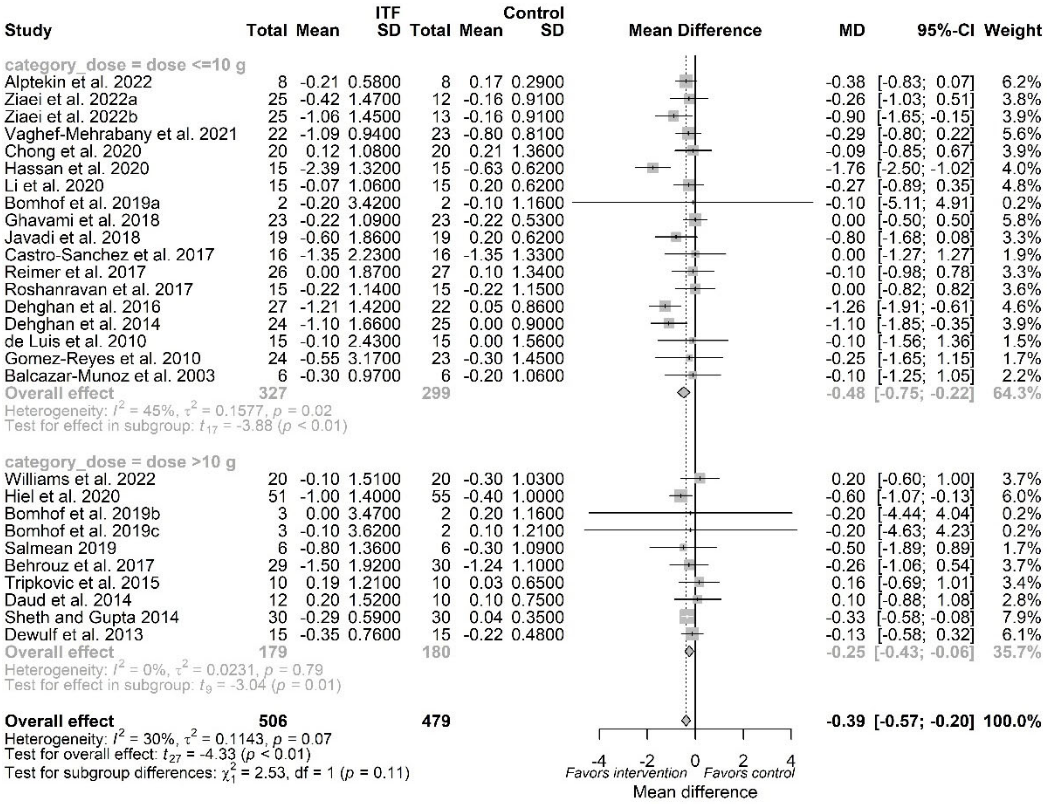


**Supplemental Figure 7** Forest plot baseline-corrected mean difference (MD) and corresponding 95% confidence interval (CI) on BMI (kg/m$^2$) with subgroup analysis based on dose (dose > 10 g/d and ≤ 10 g/d) of inulin-type fructans supplementation. The diamond represents the pooled effect estimate for the overall analysis. Effect size was calculated using random-effects model/inverse-variance method with Sidik-Jonkman estimator and Hartung-Knapp adjustment to account within and between studies variances. Interstudy heterogeneity was quantified as $I^2$. Significance level was set at $P$ < 0.05. *Abbreviations:* CI, confidence interval; ITF, inulin-type fructans; MD, baseline-corrected mean difference; SD, standard deviation; total, total number of participants completed the study.

## *Online supporting material*

The effects of chicory inulin-type fructans supplementation on weight management aspects: systematic review, meta-analysis and meta-regression of randomized controlled trials.

Raylene A. Reimer, Stephan Theis, and Yoghatama Cindya Zanzer

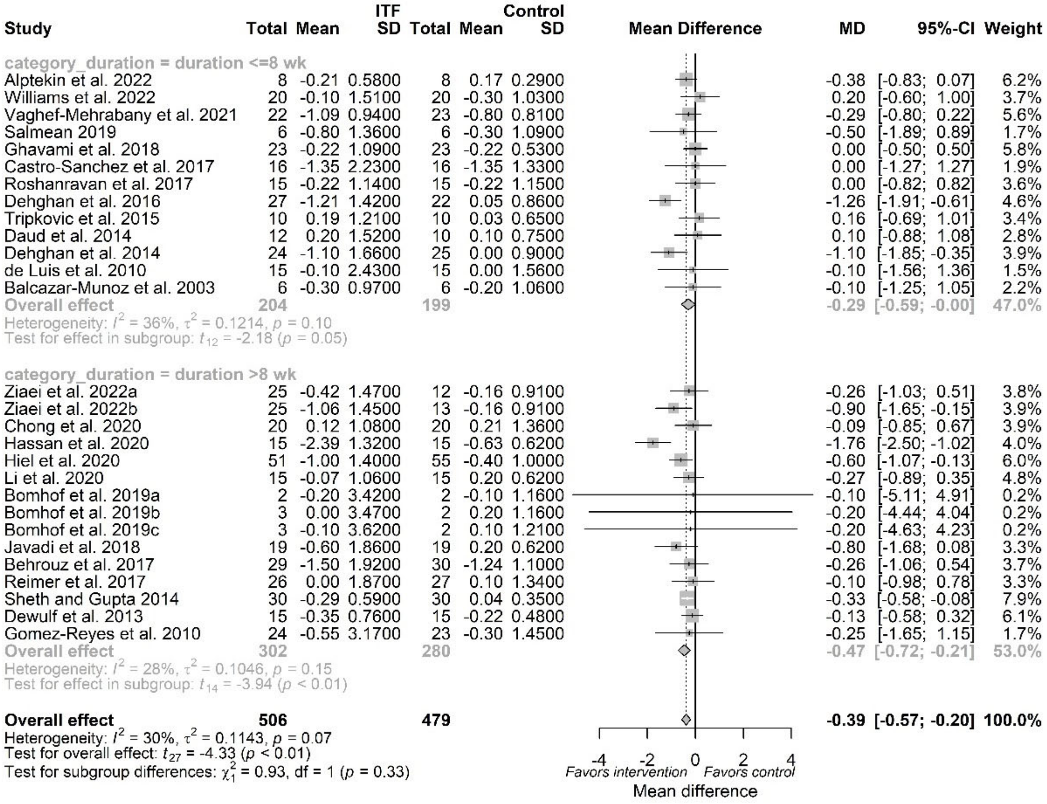


**Supplemental Figure 8** Forest plot baseline-corrected mean difference (MD) and corresponding 95% confidence interval (CI) on BMI (kg/m$^2$) with subgroup analysis based on duration (duration > 8 wk and ≤ 8 wk) of inulin-type fructans supplementation. The diamond represents the pooled effect estimate for the overall analysis. Effect size was calculated using random-effects model/inverse-variance method with Sidik-Jonkman estimator and Hartung-Knapp adjustment to account within and between studies variances. Interstudy heterogeneity was quantified as $I^2$. Significance level was set at $P < 0.05$.

*Online supporting material*

The effects of chicory inulin-type fructans supplementation on weight management aspects: systematic review, meta-analysis and meta-regression of randomized controlled trials.

Raylene A. Reimer, Stephan Theis, and Yoghatama Cindya Zanzer

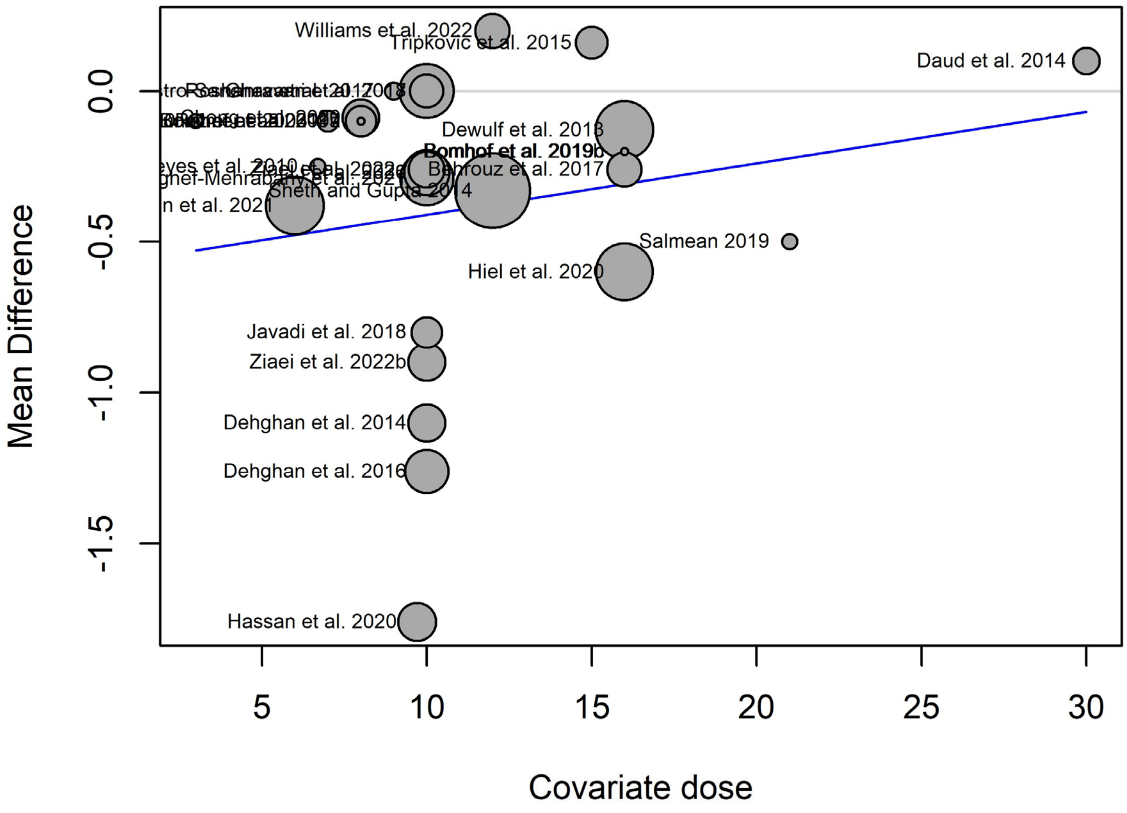


**Supplemental Figure 9** Meta-regression bubble-plot baseline-corrected mean difference (MD) on BMI (kg/m$^2$) by covariate dose (g/d) of inulin-type fructans supplementation. Circles represent each of the included studies in the meta-analysis, and the size of the circles corresponds to the inverse variance-weight of the BMI effect.

***Online supporting material***

The effects of chicory inulin-type fructans supplementation on weight management aspects: systematic review, meta-analysis and meta-regression of randomized controlled trials.

Raylene A. Reimer, Stephan Theis, and Yoghatama Cindya Zanzer

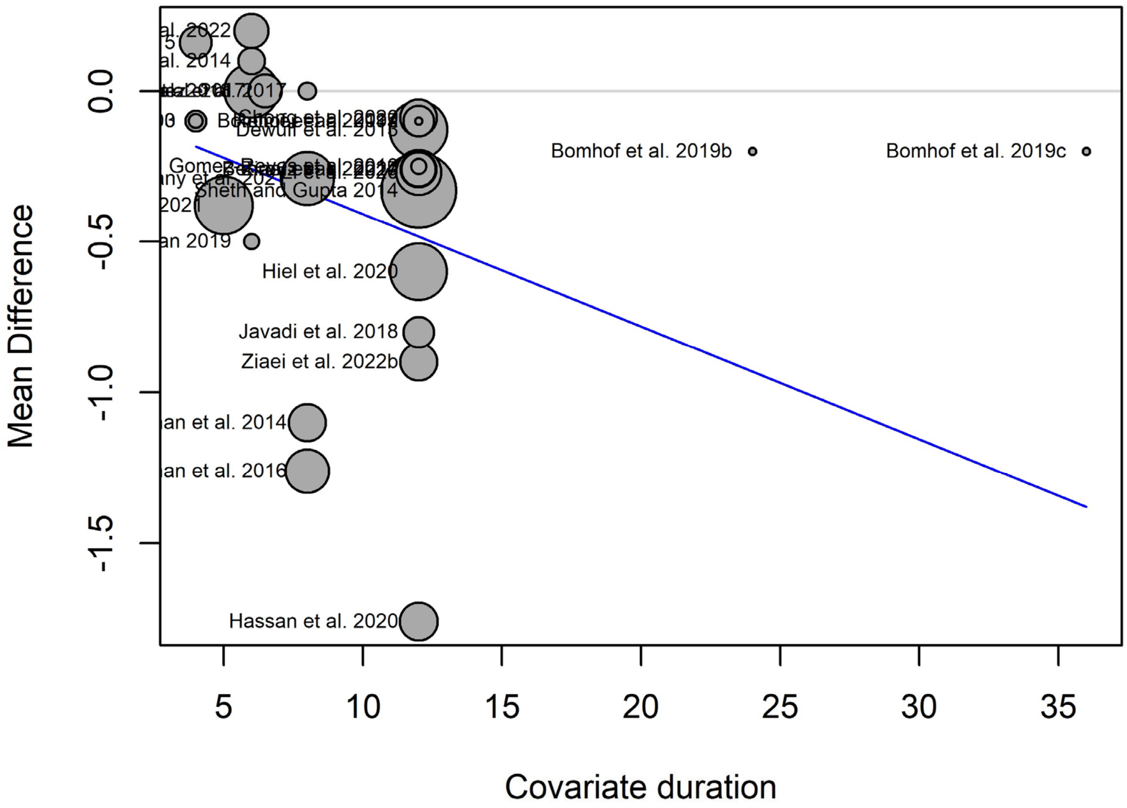


**Supplemental Figure 10** Meta-regression bubble-plot baseline-corrected mean difference (MD) on BMI ($kg/m^2$) by covariate duration (wk) of inulin-type fructans supplementation. Circles represent each of the included studies in the meta-analysis, and the size of the circles corresponds to the inverse variance-weight of the BMI effect.

The effects of chicory inulin-type fructans supplementation on weight management aspects: systematic review, meta-analysis and meta-regression of randomized controlled trials.

Raylene A. Reimer, Stephan Theis, and Yoghatama Cindya Zanzer

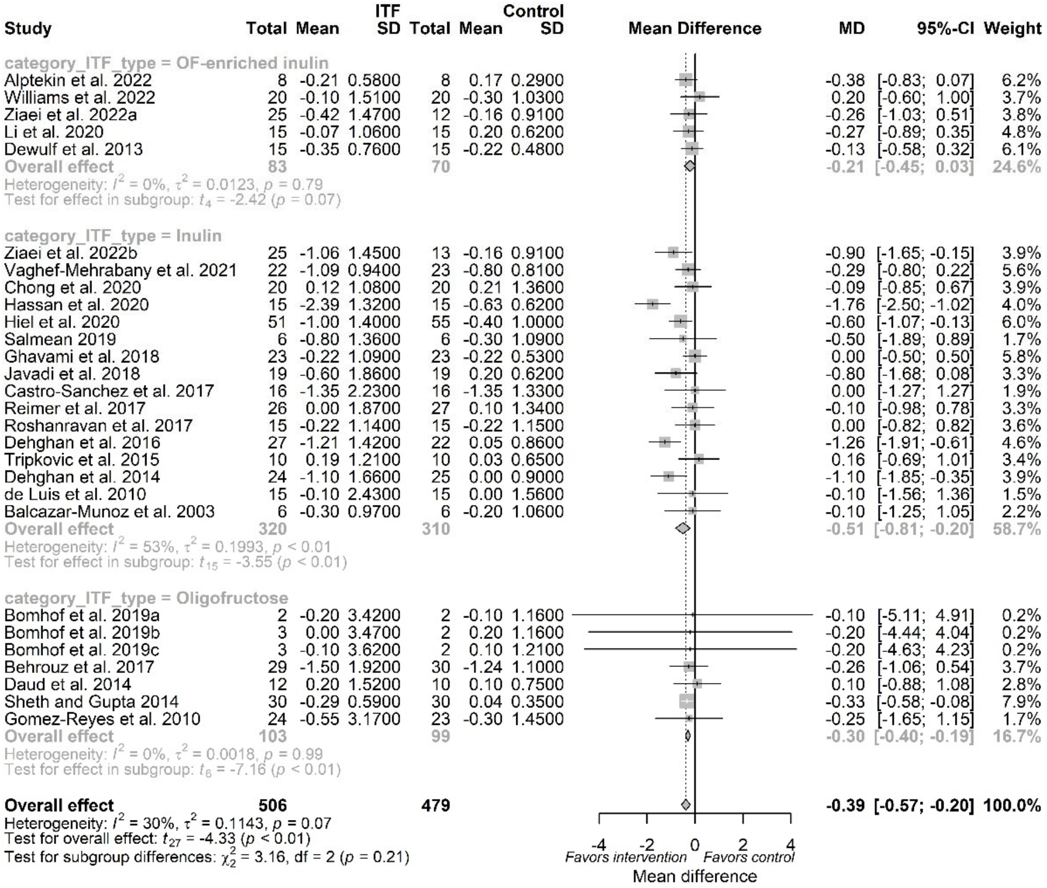


**Supplemental Figure 11** Forest plot baseline-corrected mean difference (MD) and corresponding 95% confidence interval (CI) on BMI (kg/m$^2$) with subgroup analysis based on type of inulin-type fructans (oligofructose, oligofructose-enriched inulin, and inulin) supplementation. The diamond represents the pooled effect estimate for the overall analysis. Effect size was calculated using random-effects model/inverse-variance method with Sidik-Jonkman estimator and Hartung-Knapp adjustment to account within and between studies variances. Interstudy heterogeneity was quantified as $I^2$. Significance level was set at $P < 0.05$. *Abbreviations:* CI, confidence interval; ITF, inulin-type fructans; MD, baseline-corrected mean difference; SD, standard deviation; total, total number of participants completed the study.

## *Online supporting material*

The effects of chicory inulin-type fructans supplementation on weight management aspects: systematic review, meta-analysis and meta-regression of randomized controlled trials.

Raylene A. Reimer, Stephan Theis, and Yoghatama Cindya Zanzer

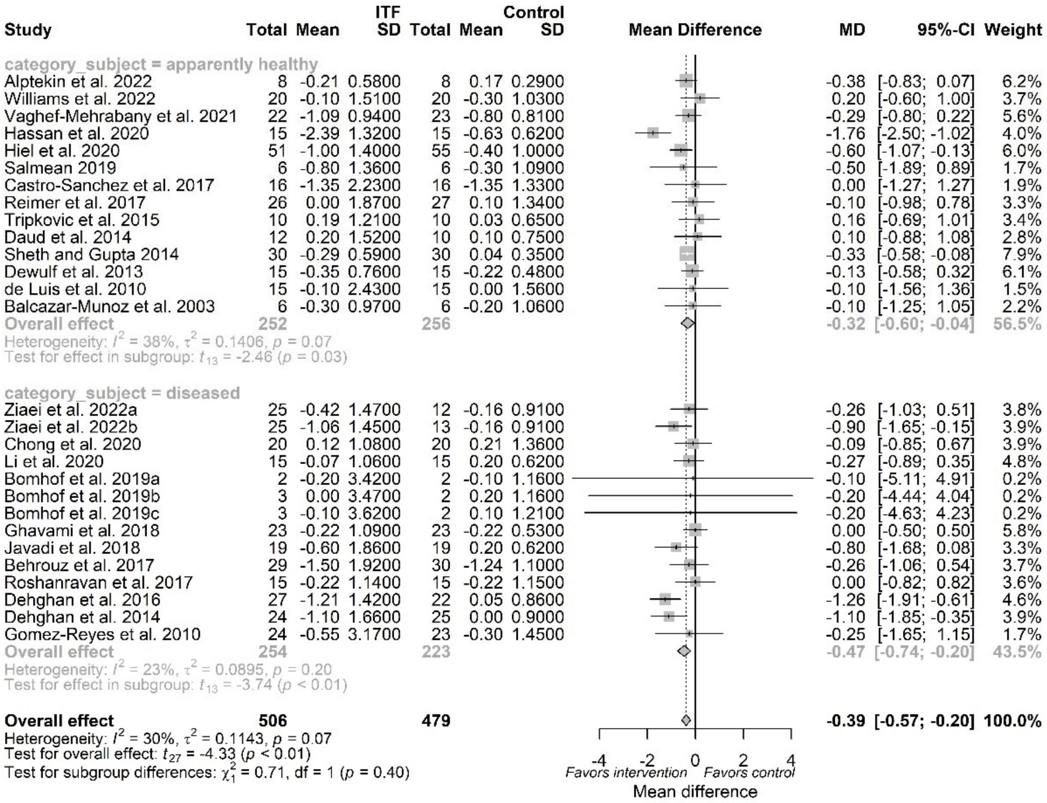


**Supplemental Figure 12** Forest plot baseline-corrected mean difference (MD) and corresponding 95% confidence interval (CI) on BMI (kg/m$^2$) with subgroup analysis based on subject health status (apparently healthy and diseased). The diamond represents the pooled effect estimate for the overall analysis. Effect size was calculated using random-effects model/inverse-variance method with Sidik-Jonkman estimator and Hartung-Knapp adjustment to account within and between studies variances. Interstudy heterogeneity was quantified as $I^2$. Significance level was set at $P < 0.05$. *Abbreviations:* CI, confidence interval; ITF, inulin-type fructans; MD, baseline-corrected mean difference; SD, standard deviation; total, total number of participants completed the study.

## *Online supporting material*

The effects of chicory inulin-type fructans supplementation on weight management aspects: systematic review, meta-analysis and meta-regression of randomized controlled trials.

Raylene A. Reimer, Stephan Theis, and Yoghatama Cindya Zanzer

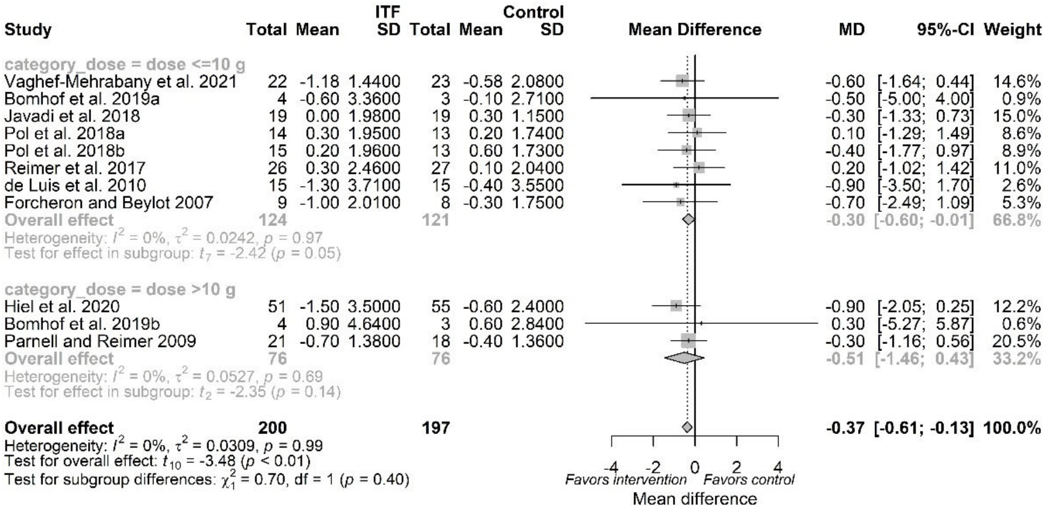


**Supplemental Figure 13** Forest plot baseline-corrected mean difference (MD) and corresponding 95% confidence interval (CI) on total fat mass (kg) with subgroup analysis based on dose (dose > 10 g/d and ≤ 10 g/d) of inulin-type fructans supplementation. The diamond represents the pooled effect estimate for the overall analysis. Effect size was calculated using random-effects model/inverse-variance method with Sidik-Jonkman estimator and Hartung-Knapp adjustment to account within and between studies variances. Interstudy heterogeneity was quantified as $I^2$. Significance level was set at $P < 0.05$. *Abbreviations:* CI, confidence interval; ITF, inulin-type fructans; MD, baseline-corrected mean difference; SD, standard deviation; total, total number of participants completed the study.

*Online supporting material*

The effects of chicory inulin-type fructans supplementation on weight management aspects: systematic review, meta-analysis and meta-regression of randomized controlled trials.

Raylene A. Reimer, Stephan Theis, and Yoghatama Cindya Zanzer

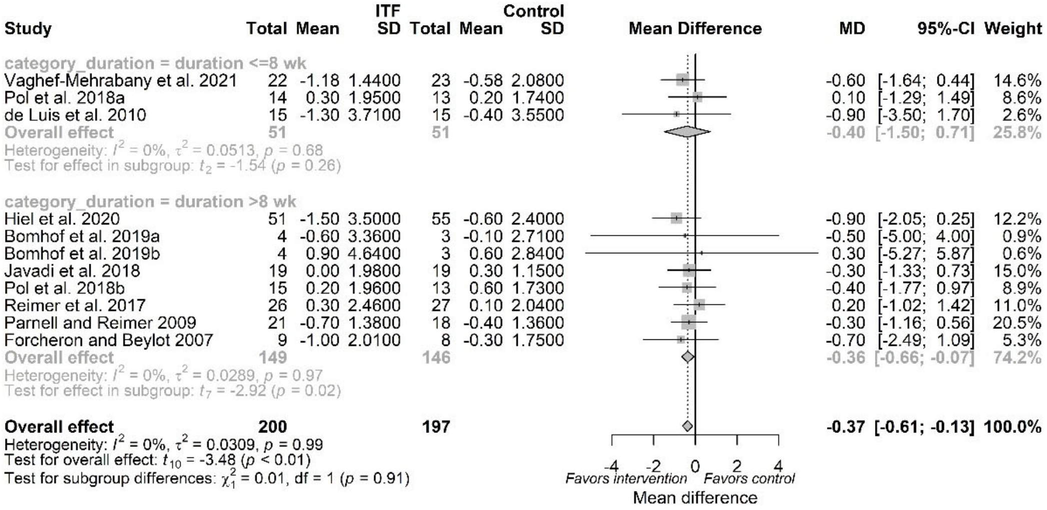


**Supplemental Figure 14** Forest plot baseline-corrected mean difference (MD) and corresponding 95% confidence interval (CI) on total fat mass (kg) with subgroup analysis based on duration (duration > 8 wk and ≤ 8 wk) of inulin-type fructans supplementation. The diamond represents the pooled effect estimate for the overall analysis. Effect size was calculated using random-effects model/inverse-variance method with Sidik-Jonkman estimator and Hartung-Knapp adjustment to account within and between studies variances. Interstudy heterogeneity was quantified as $I^2$. Significance level was set at $P < 0.05$. *Abbreviations:* CI, confidence interval; ITF, inulin-type fructans; MD, baseline-corrected mean difference; SD, standard deviation; total, total number of participants completed the study.

The effects of chicory inulin-type fructans supplementation on weight management aspects: systematic review, meta-analysis and meta-regression of randomized controlled trials.

Raylene A. Reimer, Stephan Theis, and Yoghatama Cindya Zanzer

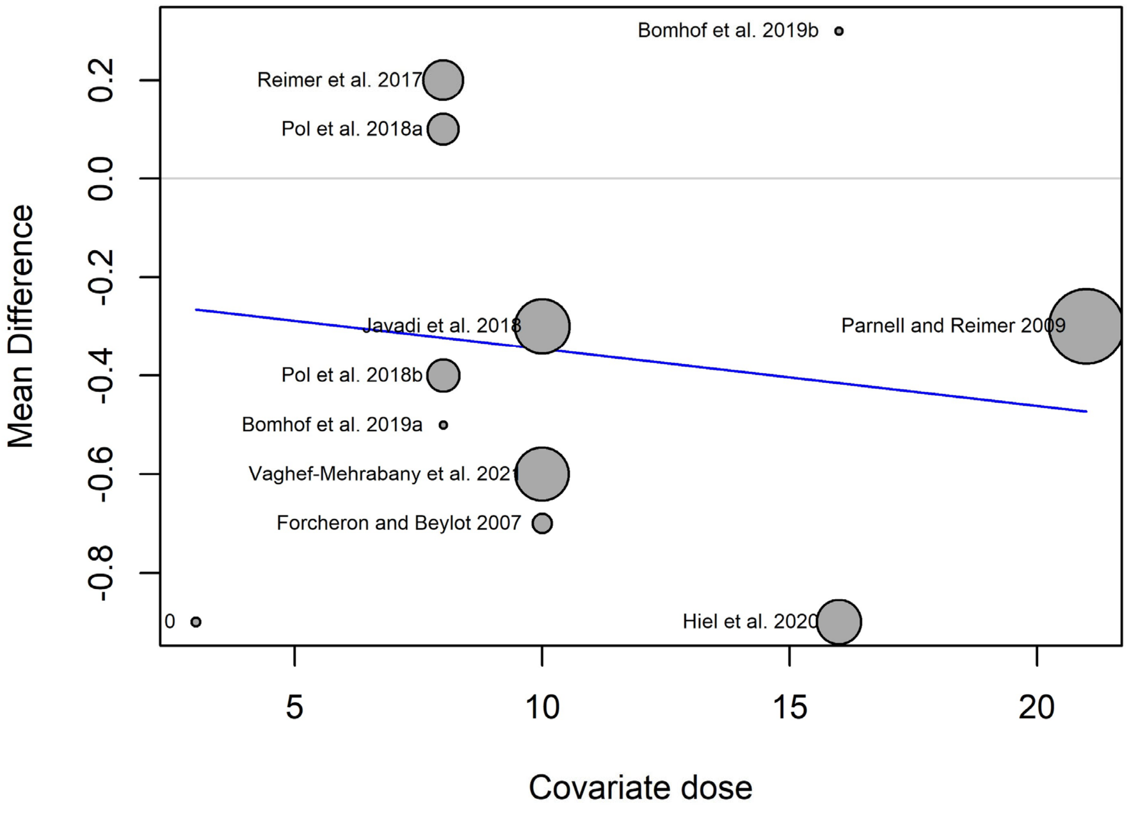


**Supplemental Figure 15** Meta-regression bubble-plot baseline-corrected mean difference (MD) on total fat mass (kg) by covariate dose (g/d) of inulin-type fructans supplementation. Circles represent each of the included studies in the meta-analysis, and the size of the circles corresponds to the inverse variance-weight of the total fat mass effect.

Raylene A. Reimer, Stephan Theis, and Yoghatama Cindya Zanzer

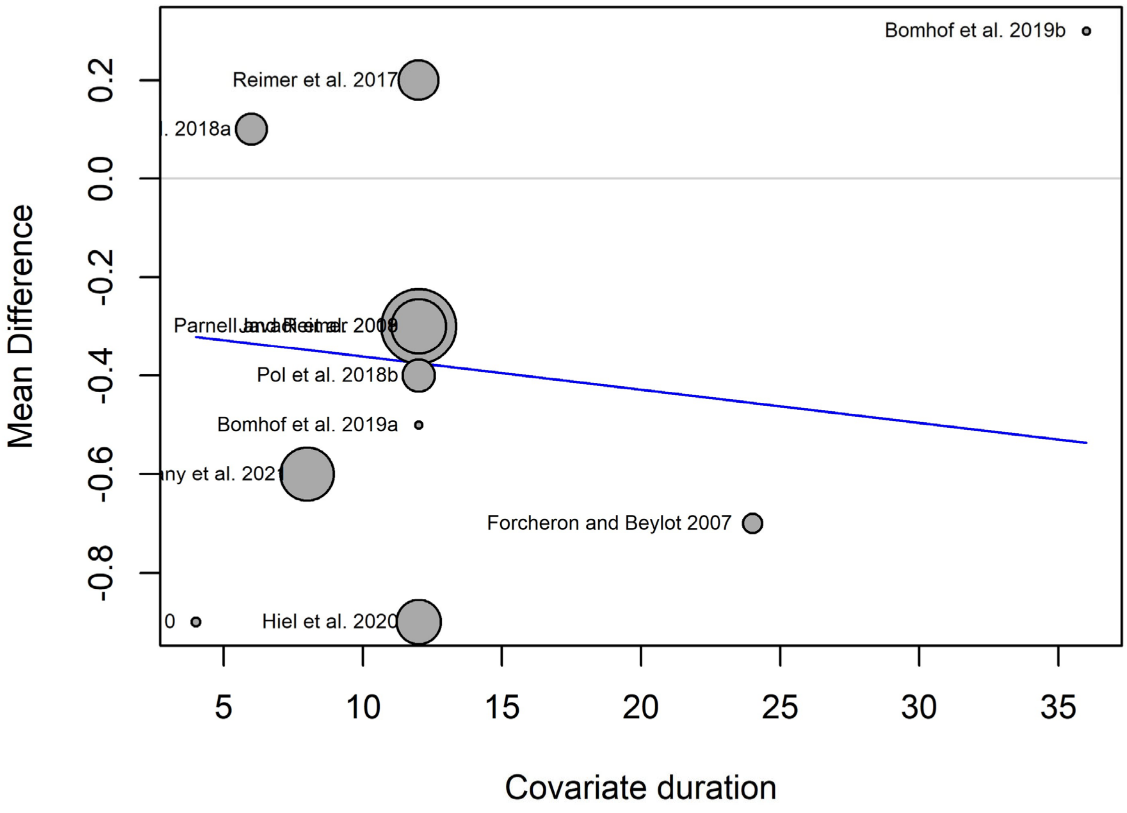


**Supplemental Figure 16** Meta-regression bubble-plot baseline-corrected mean difference (MD) on total fat mass (kg) by covariate duration (wk) of inulin-type fructans supplementation. Circles represent each of the included studies in the meta-analysis, and the size of the circles corresponds to the inverse variance-weight of the total fat mass effect.

# *Online supporting material*

The effects of chicory inulin-type fructans supplementation on weight management aspects: systematic review, meta-analysis and meta-regression of randomized controlled trials.

Raylene A. Reimer, Stephan Theis, and Yoghatama Cindya Zanzer

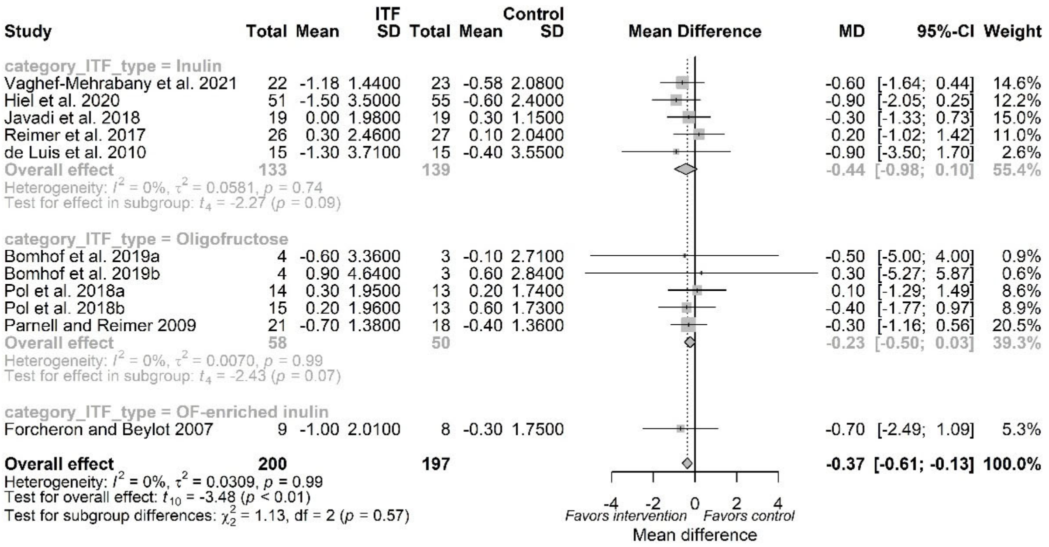


**Supplemental Figure 17** Forest plot baseline-corrected mean difference (MD) and corresponding 95% confidence interval (CI) on total fat mass (kg) with subgroup analysis based on type of inulin-type fructans (oligofructose, oligofructose-enriched inulin, and inulin) supplementation. The diamond represents the pooled effect estimate for the overall analysis. Effect size was calculated using random-effects model/inverse-variance method with Sidik-Jonkman estimator and Hartung-Knapp adjustment to account within and between studies variances. Interstudy heterogeneity was quantified as $I^2$. Significance level was set at $P < 0.05$. *Abbreviations:* CI, confidence interval; ITF, inulin-type fructans; MD, baseline-corrected mean difference; SD, standard deviation; total, total number of participants completed the study.

## *Online supporting material*

The effects of chicory inulin-type fructans supplementation on weight management aspects: systematic review, meta-analysis and meta-regression of randomized controlled trials.

Raylene A. Reimer, Stephan Theis, and Yoghatama Cindya Zanzer

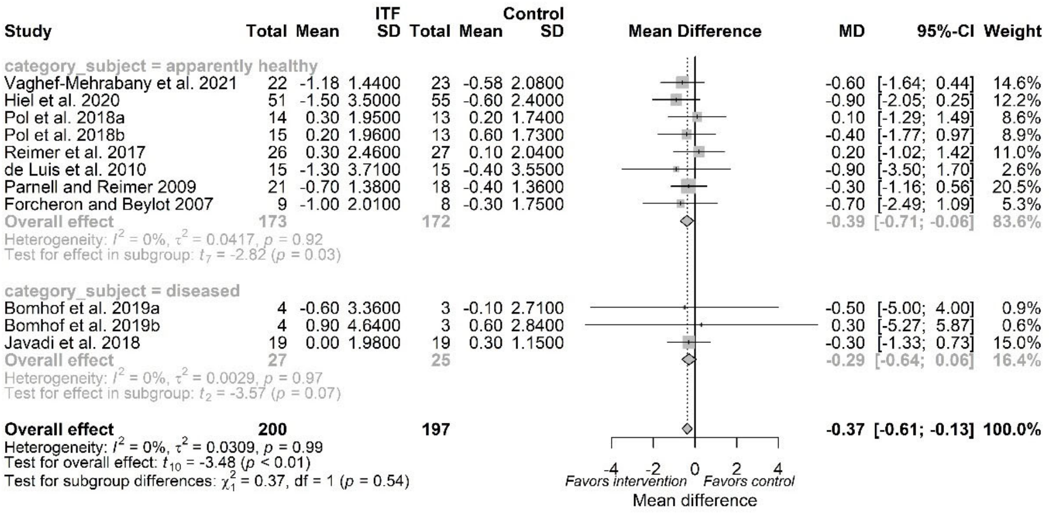


**Supplemental Figure 18** Forest plot baseline-corrected mean difference (MD) and corresponding 95% confidence interval (CI) on total fat mass (kg) with subgroup analysis based on subject health status (apparently healthy and diseased). The diamond represents the pooled effect estimate for the overall analysis. Effect size was calculated using random-effects model/inverse-variance method with Sidik-Jonkman estimator and Hartung-Knapp adjustment to account within and between studies variances. Interstudy heterogeneity was quantified as $I^2$. Significance level was set at $P < 0.05$. *Abbreviations:* CI, confidence interval; ITF, inulin-type fructans; MD, baseline-corrected mean difference; SD, standard deviation; total, total number of participants completed the study.

*Online supporting material*

The effects of chicory inulin-type fructans supplementation on weight management aspects: systematic review, meta-analysis and meta-regression of randomized controlled trials.

Raylene A. Reimer, Stephan Theis, and Yoghatama Cindya Zanzer

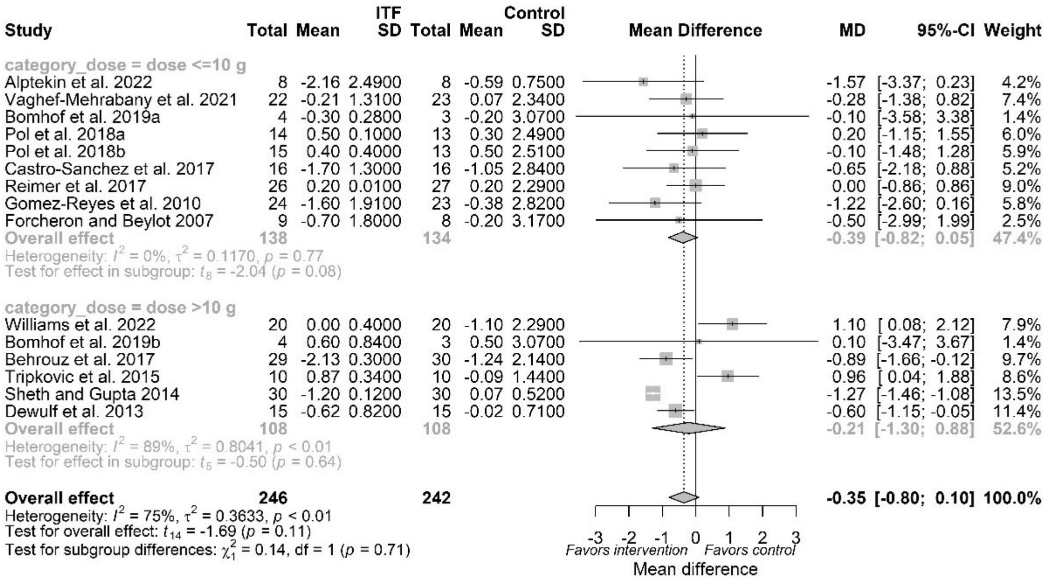


**Supplemental Figure 19** Forest plot baseline-corrected mean difference (MD) and corresponding 95% confidence interval (CI) on body fat percentage (%) with subgroup analysis based on dose (dose > 10 g/d and ≤ 10 g/d) of inulin-type fructans supplementation. The diamond represents the pooled effect estimate for the overall analysis. Effect size was calculated using random-effects model/inverse-variance method with Sidik-Jonkman estimator and Hartung-Knapp adjustment to account within and between studies variances. Interstudy heterogeneity was quantified as $I^2$. Significance level was set at $P < 0.05$. *Abbreviations:* CI, confidence interval; ITF, inulin-type fructans; MD, baseline-corrected mean difference; SD, standard deviation; total, total number of participants completed the study.

*Online supporting material*

The effects of chicory inulin-type fructans supplementation on weight management aspects: systematic review, meta-analysis and meta-regression of randomized controlled trials.

Raylene A. Reimer, Stephan Theis, and Yoghatama Cindya Zanzer

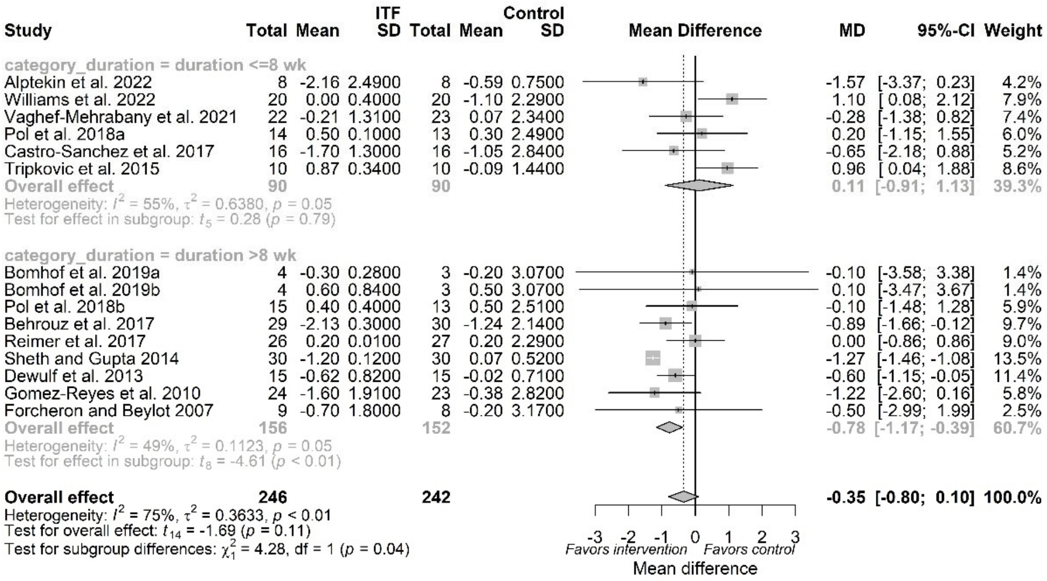


**Supplemental Figure 20** Forest plot baseline-corrected mean difference (MD) and corresponding 95% confidence interval (CI) on body fat percentage (%) with subgroup analysis based on duration (duration > 8 wk and ≤ 8 wk) of inulin-type fructans supplementation. The diamond represents the pooled effect estimate for the overall analysis. Effect size was calculated using random-effects model/inverse-variance method with Sidik-Jonkman estimator and Hartung-Knapp adjustment to account within and between studies variances. Interstudy heterogeneity was quantified as $I^2$. Significance level was set at $P < 0.05$. *Abbreviations:* CI, confidence interval; ITF, inulin-type fructans; MD, baseline-corrected mean difference; SD, standard deviation; total, total number of participants completed the study.

The effects of chicory inulin-type fructans supplementation on weight management aspects: systematic review, meta-analysis and meta-regression of randomized controlled trials.

Raylene A. Reimer, Stephan Theis, and Yoghatama Cindya Zanzer

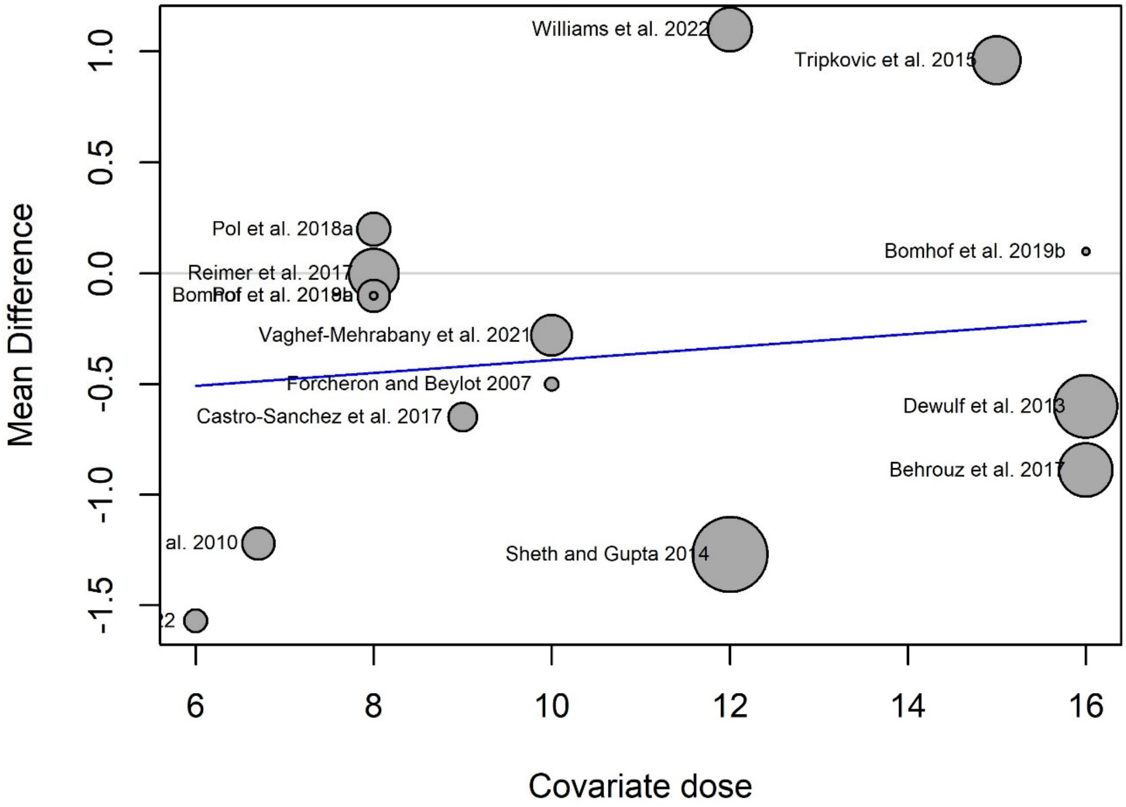


**Supplemental Figure 21** Meta-regression bubble-plot baseline-corrected mean difference (MD) on body fat percentage (%) by covariate dose (g/d) of inulin-type fructans supplementation. Circles represent each of the included studies in the meta-analysis, and the size of the circles corresponds to the inverse variance-weight of the body fat percentage effect.

The effects of chicory inulin-type fructans supplementation on weight management aspects: systematic review, meta-analysis and meta-regression of randomized controlled trials.

Raylene A. Reimer, Stephan Theis, and Yoghatama Cindya Zanzer

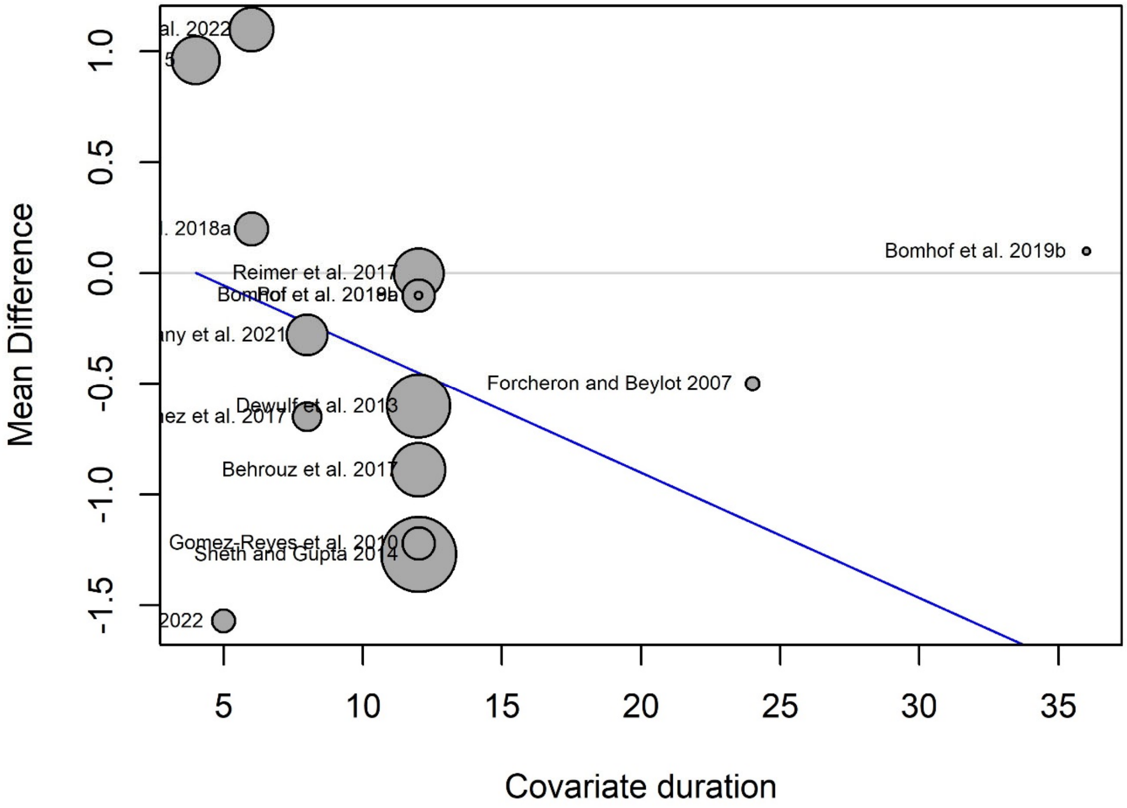


**Supplemental Figure 22** Meta-regression bubble-plot baseline-corrected mean difference (MD) on body fat percentage (%) by covariate duration (wk) of inulin-type fructans supplementation. Circles represent each of the included studies in the meta-analysis, and the size of the circles corresponds to the inverse variance-weight of the body fat percentage effect.

The effects of chicory inulin-type fructans supplementation on weight management aspects: systematic review, meta-analysis and meta-regression of randomized controlled trials.

Raylene A. Reimer, Stephan Theis, and Yoghatama Cindya Zanzer

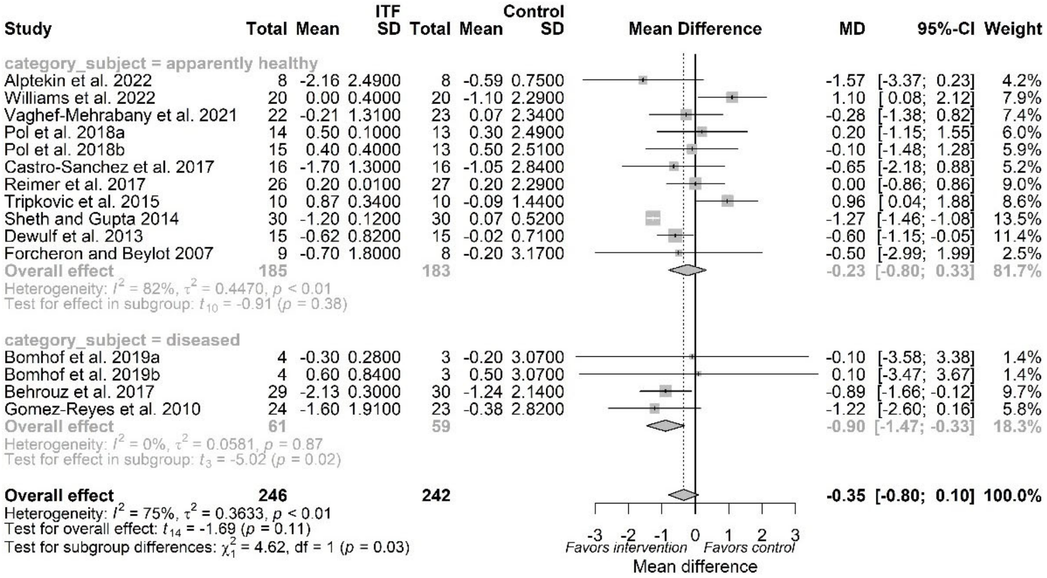


**Supplemental Figure 23** Forest plot baseline-corrected mean difference (MD) and corresponding 95% confidence interval (CI) on body fat percentage (%) with subgroup analysis based on subject health status (apparently healthy and diseased). The diamond represents the pooled effect estimate for the overall analysis. Effect size was calculated using random-effects model/inverse-variance method with Sidik-Jonkman estimator and Hartung-Knapp adjustment to account within and between studies variances. Interstudy heterogeneity was quantified as $I^2$. Significance level was set at $P < 0.05$. *Abbreviations:* CI, confidence interval; ITF, inulin-type fructans; MD, baseline-corrected mean difference; SD, standard deviation; total, total number of participants completed the study.

***Online supporting material***

The effects of chicory inulin-type fructans supplementation on weight management aspects: systematic review, meta-analysis and meta-regression of randomized controlled trials.

Raylene A. Reimer, Stephan Theis, and Yoghatama Cindya Zanzer

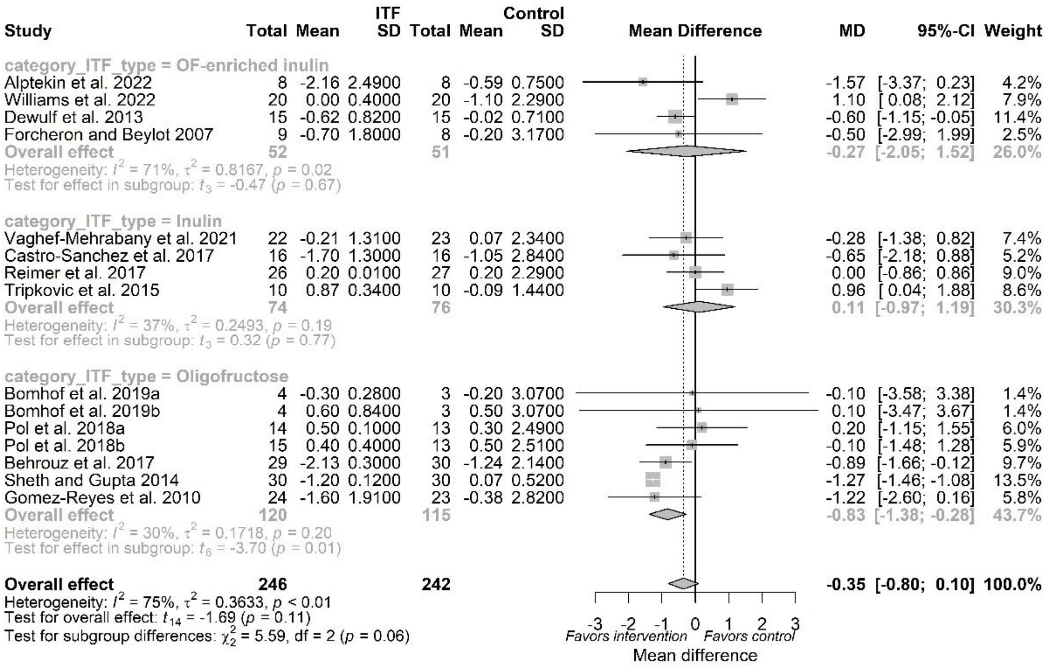


**Supplemental Figure 24** Forest plot baseline-corrected mean difference (MD) and corresponding 95% confidence interval (CI) on body fat percentage (%) with subgroup analysis based on type of inulin-type fructans (oligofructose, oligofructose-enriched inulin, and inulin) supplementation. The diamond represents the pooled effect estimate for the overall analysis. Effect size was calculated using random-effects model/inverse-variance method with Sidik-Jonkman estimator and Hartung-Knapp adjustment to account within and between studies variances. Interstudy heterogeneity was quantified as $I^2$. Significance level was set at $P$ < 0.05. *Abbreviations:* CI, confidence interval; ITF, inulin-type fructans; MD, baseline-corrected mean difference; SD, standard deviation; total, total number of participants completed the study.

## *Online supporting material*

The effects of chicory inulin-type fructans supplementation on weight management aspects: systematic review, meta-analysis and meta-regression of randomized controlled trials.

Raylene A. Reimer, Stephan Theis, and Yoghatama Cindya Zanzer

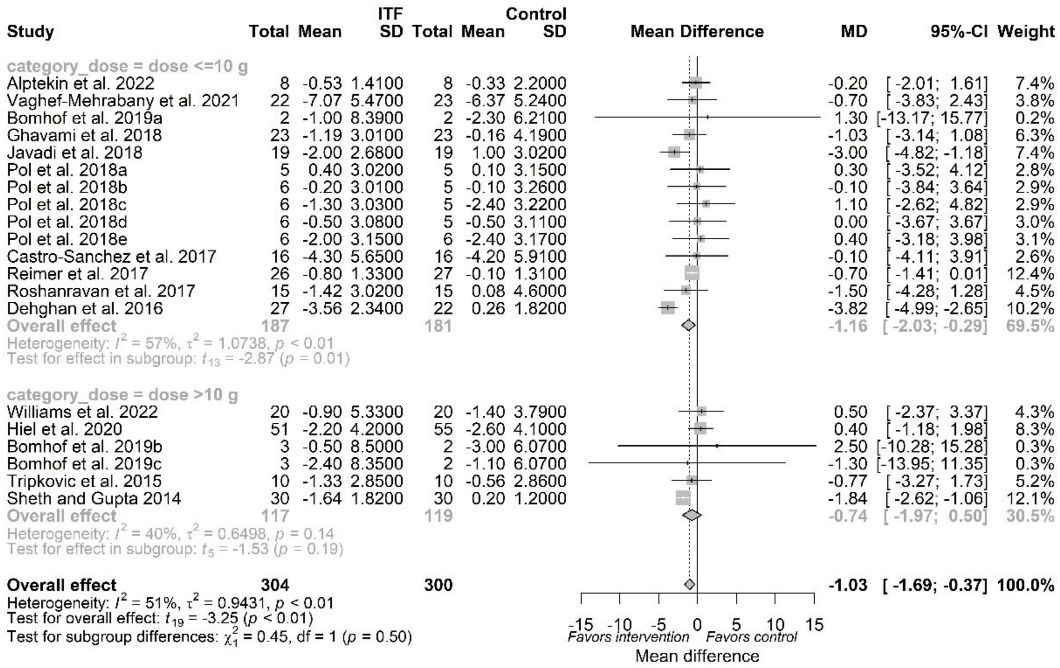


**Supplemental Figure 25** Forest plot baseline-corrected mean difference (MD) and corresponding 95% confidence interval (CI) on waist circumference (cm) with subgroup analysis based on dose (dose > 10 g/d and ≤ 10 g/d) of inulin-type fructans supplementation. The diamond represents the pooled effect estimate for the overall analysis. Effect size was calculated using random-effects model/inverse-variance method with Sidik-Jonkman estimator and Hartung-Knapp adjustment to account within and between studies variances. Interstudy heterogeneity was quantified as $I^2$. Significance level was set at $P < 0.05$. *Abbreviations:* CI, confidence interval; ITF, inulin-type fructans; MD, baseline-corrected mean difference; SD, standard deviation; total, total number of participants completed the study.

*Online supporting material*

The effects of chicory inulin-type fructans supplementation on weight management aspects: systematic review, meta-analysis and meta-regression of randomized controlled trials.

Raylene A. Reimer, Stephan Theis, and Yoghatama Cindya Zanzer

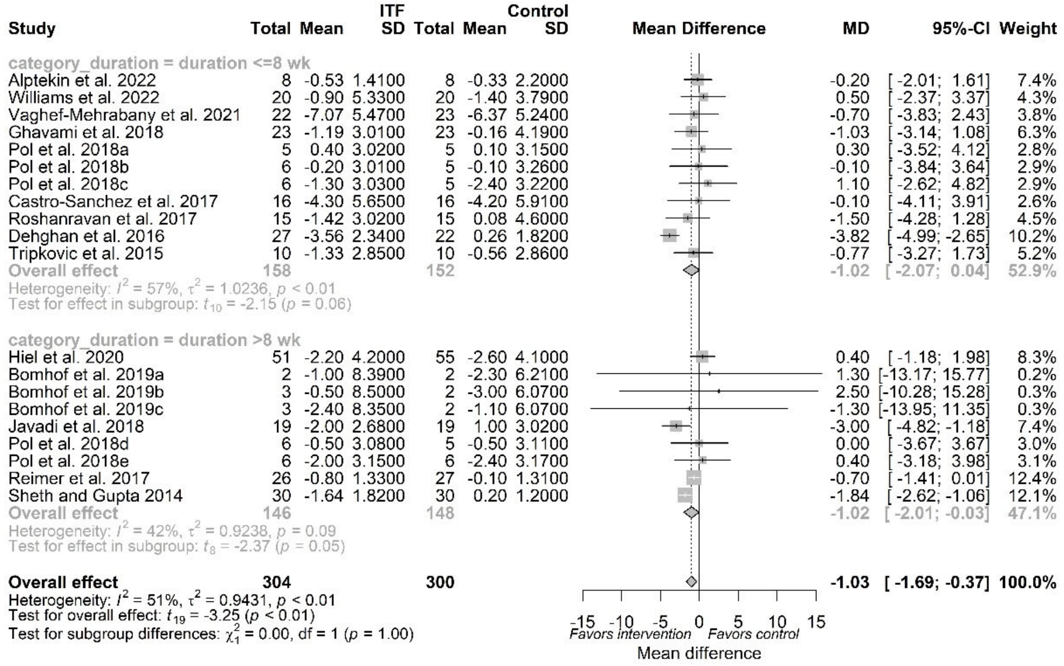


**Supplemental Figure 26** Forest plot baseline-corrected mean difference (MD) and corresponding 95% confidence interval (CI) on waist circumference (cm) with subgroup analysis based on duration (duration > 8 wk and ≤ 8 wk) of inulin-type fructans supplementation. The diamond represents the pooled effect estimate for the overall analysis. Effect size was calculated using random-effects model/inverse-variance method with Sidik-Jonkman estimator and Hartung-Knapp adjustment to account within and between studies variances. Interstudy heterogeneity was quantified as $I^2$. Significance level was set at $P < 0.05$. *Abbreviations:* CI, confidence interval; ITF, inulin-type fructans; MD, baseline-corrected mean difference; SD, standard deviation; total, total number of participants completed the study.

The effects of chicory inulin-type fructans supplementation on weight management aspects: systematic review, meta-analysis and meta-regression of randomized controlled trials.

Raylene A. Reimer, Stephan Theis, and Yoghatama Cindya Zanzer

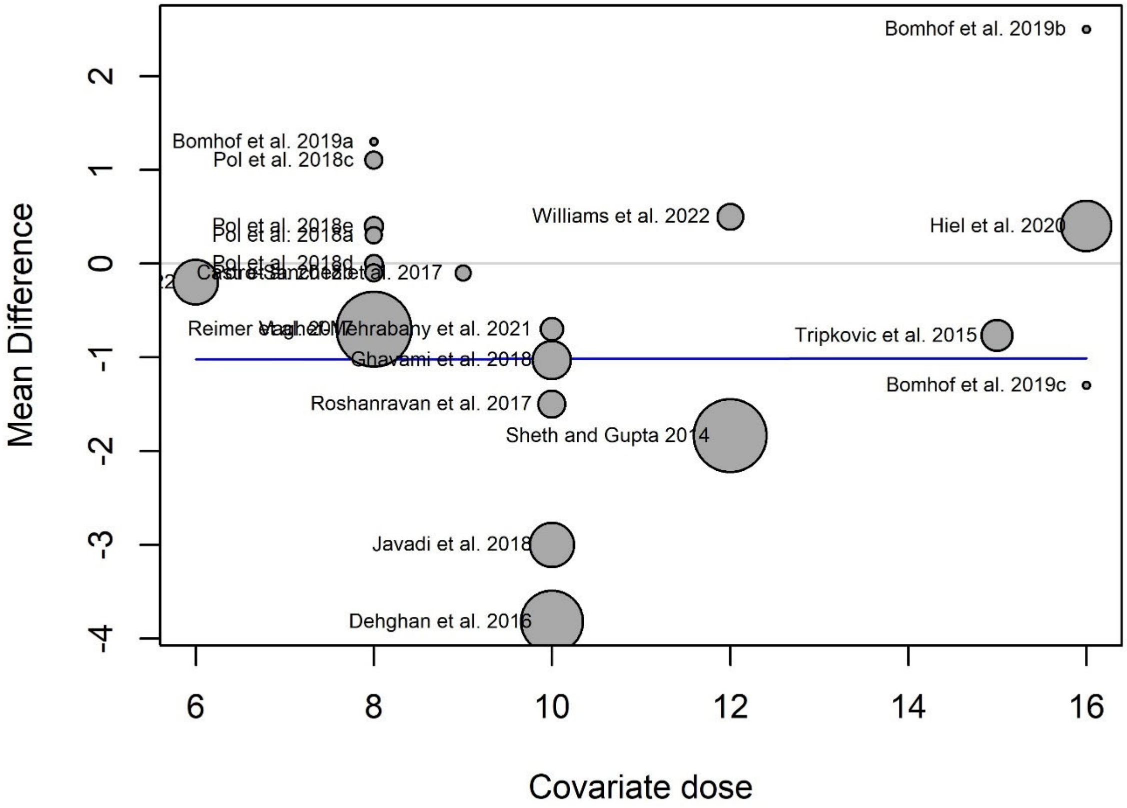


**Supplemental Figure 27** Meta-regression bubble-plot baseline-corrected mean difference (MD) on waist circumference (cm) by covariate dose (g/d) of inulin-type fructans supplementation. Circles represent each of the included studies in the meta-analysis, and the size of the circles corresponds to the inverse variance-weight of the waist circumference effect.

The effects of chicory inulin-type fructans supplementation on weight management aspects: systematic review, meta-analysis and meta-regression of randomized controlled trials.

Raylene A. Reimer, Stephan Theis, and Yoghatama Cindya Zanzer

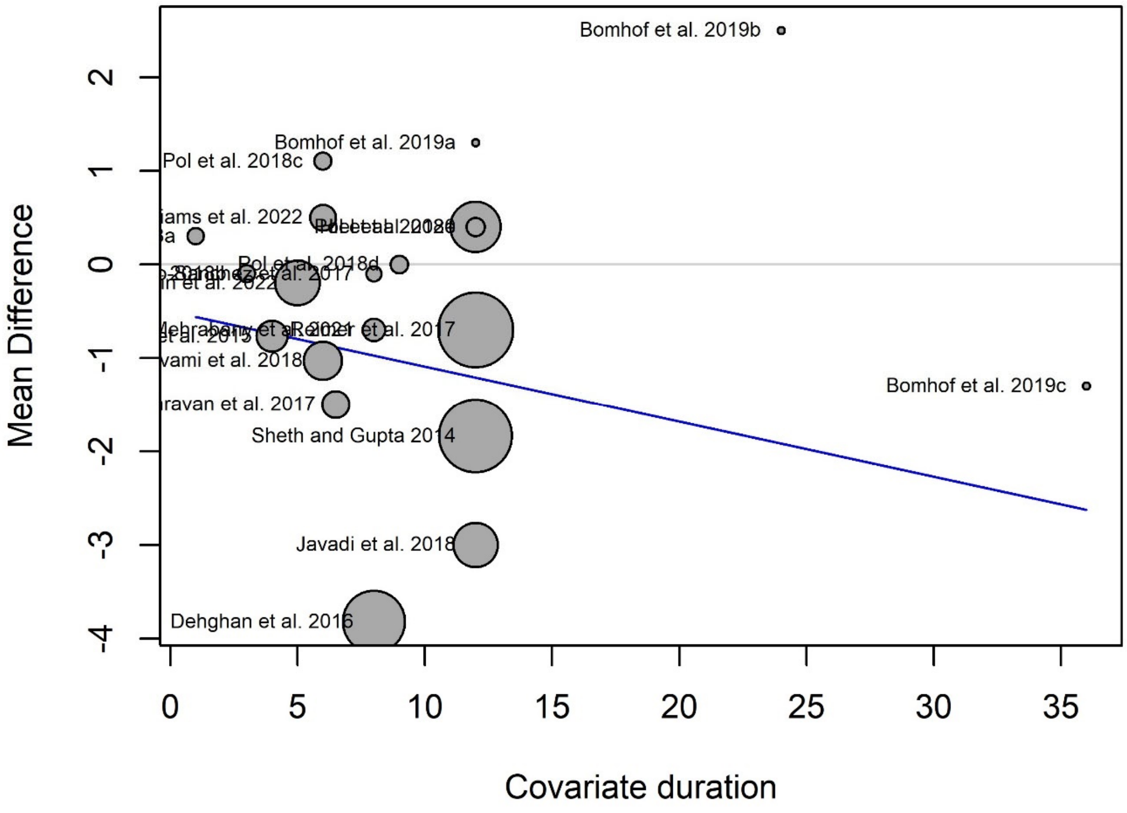


**Supplemental Figure 28** Meta-regression bubble-plot baseline-corrected mean difference (MD) on waist circumference (cm) by covariate duration (wk) of inulin-type fructans supplementation. Circles represent each of the included studies in the meta-analysis, and the size of the circles corresponds to the inverse variance-weight of the waist circumference effect.

## *Online supporting material*

The effects of chicory inulin-type fructans supplementation on weight management aspects: systematic review, meta-analysis and meta-regression of randomized controlled trials.

Raylene A. Reimer, Stephan Theis, and Yoghatama Cindya Zanzer

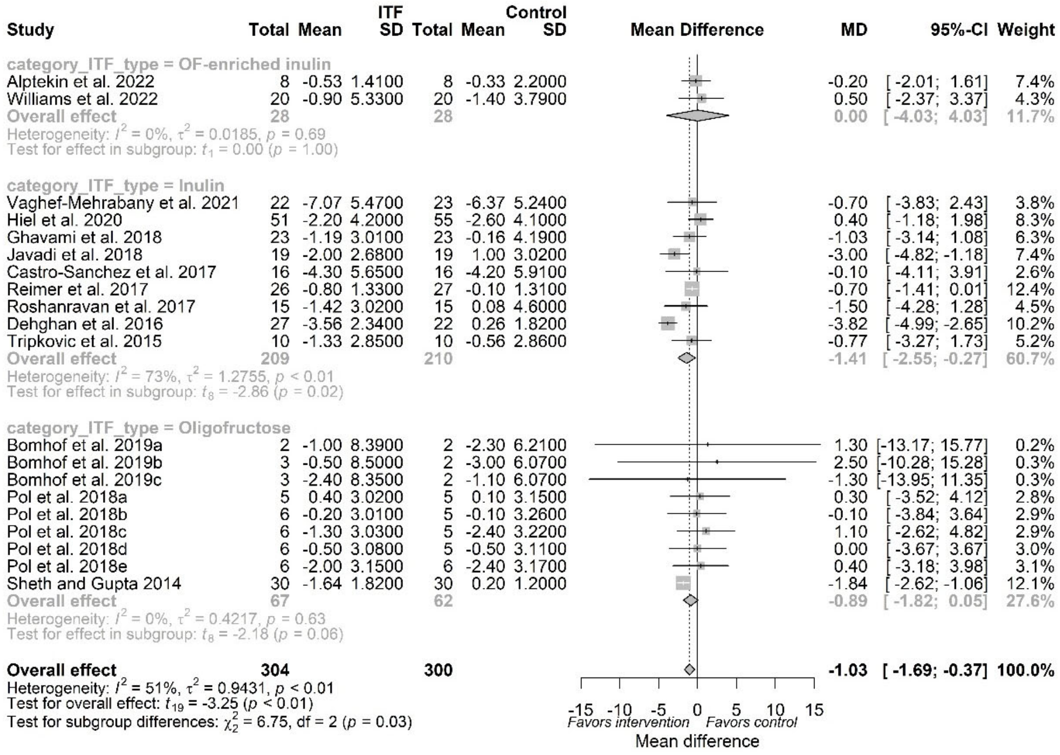


**Supplemental Figure 29** Forest plot baseline-corrected mean difference (MD) and corresponding 95% confidence interval (CI) on waist circumference (cm) with subgroup analysis based on type of inulin-type fructans (oligofructose, oligofructose-enriched inulin, and inulin) supplementation. The diamond represents the pooled effect estimate for the overall analysis. Effect size was calculated using random-effects model/inverse-variance method with Sidik-Jonkman estimator and Hartung-Knapp adjustment to account within and between studies variances. Interstudy heterogeneity was quantified as $I^2$. Significance level was set at $P < 0.05$. *Abbreviations:* CI, confidence interval; ITF, inulin-type fructans; MD, baseline-corrected mean difference; SD, standard deviation; total, total number of participants completed the study.

## *Online supporting material*

The effects of chicory inulin-type fructans supplementation on weight management aspects: systematic review, meta-analysis and meta-regression of randomized controlled trials.

Raylene A. Reimer, Stephan Theis, and Yoghatama Cindya Zanzer

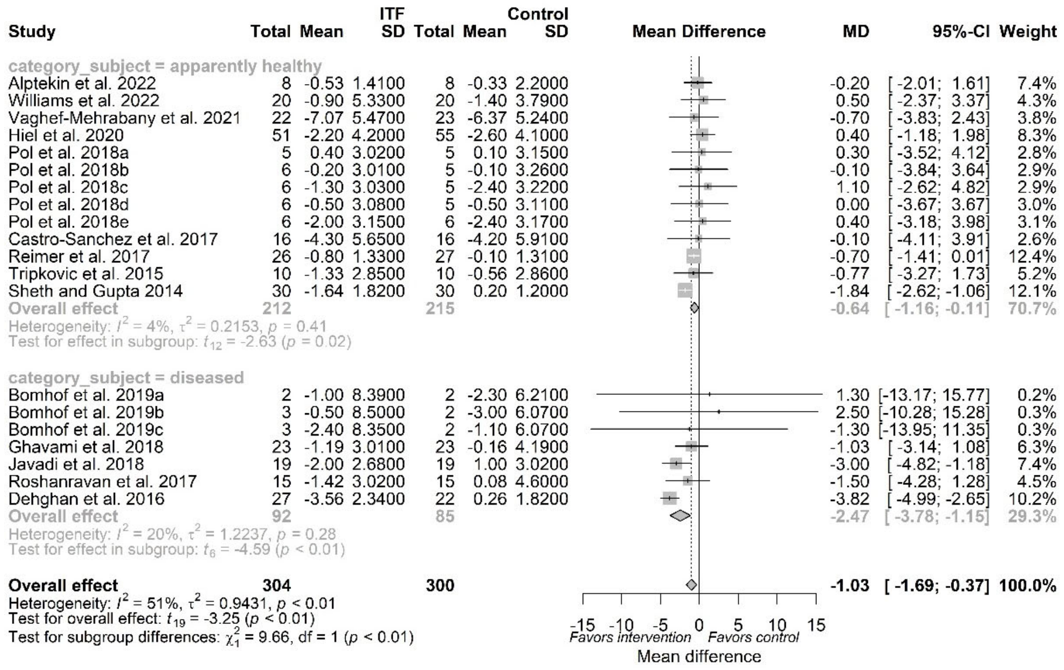


**Supplemental Figure 30** Forest plot baseline-corrected mean difference (MD) and corresponding 95% confidence interval (CI) on waist circumference (cm) with subgroup analysis based on subject health status (apparently healthy and diseased). The diamond represents the pooled effect estimate for the overall analysis. Effect size was calculated using random-effects model/inverse-variance method with Sidik-Jonkman estimator and Hartung-Knapp adjustment to account within and between studies variances. Interstudy heterogeneity was quantified as $I^2$. Significance level was set at $P < 0.05$. *Abbreviations:* CI, confidence interval; ITF, inulin-type fructans; MD, baseline-corrected mean difference; SD, standard deviation; total, total number of participants completed the study.

The effects of chicory inulin-type fructans supplementation on weight management aspects: systematic review, meta-analysis and meta-regression of randomized controlled trials.

Raylene A. Reimer, Stephan Theis, and Yoghatama Cindya Zanzer

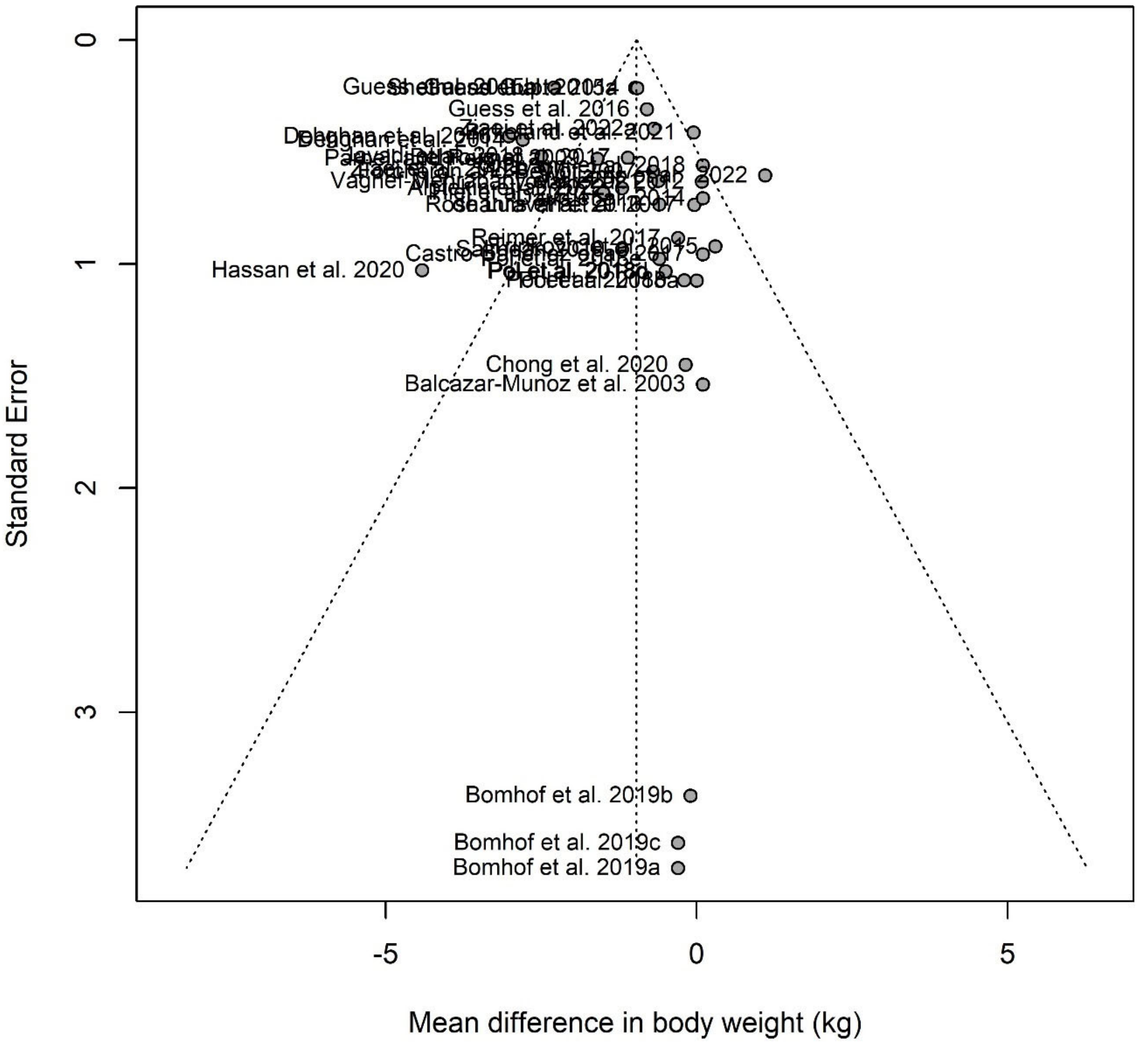


**Supplemental Figure 31** Funnel plot assessing publication bias for body weight (kg).

***Online supporting material***

The effects of chicory inulin-type fructans supplementation on weight management aspects: systematic review, meta-analysis and meta-regression of randomized controlled trials.

Raylene A. Reimer, Stephan Theis, and Yoghatama Cindya Zanzer

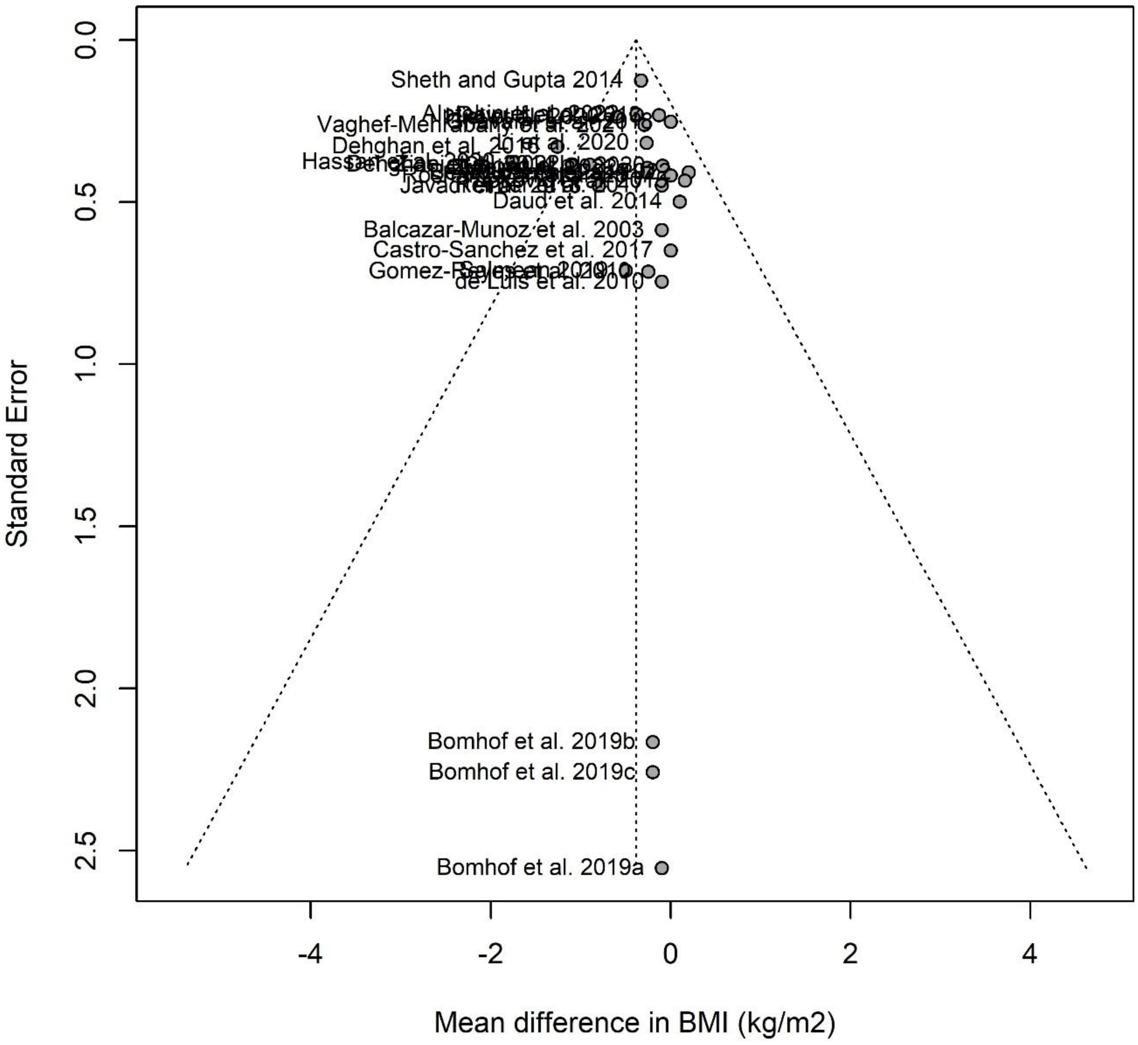


**Supplemental Figure 32** Funnel plot assessing publication bias for BMI (kg/m$^2$).

***Online supporting material***

The effects of chicory inulin-type fructans supplementation on weight management aspects: systematic review, meta-analysis and meta-regression of randomized controlled trials.

Raylene A. Reimer, Stephan Theis, and Yoghatama Cindya Zanzer

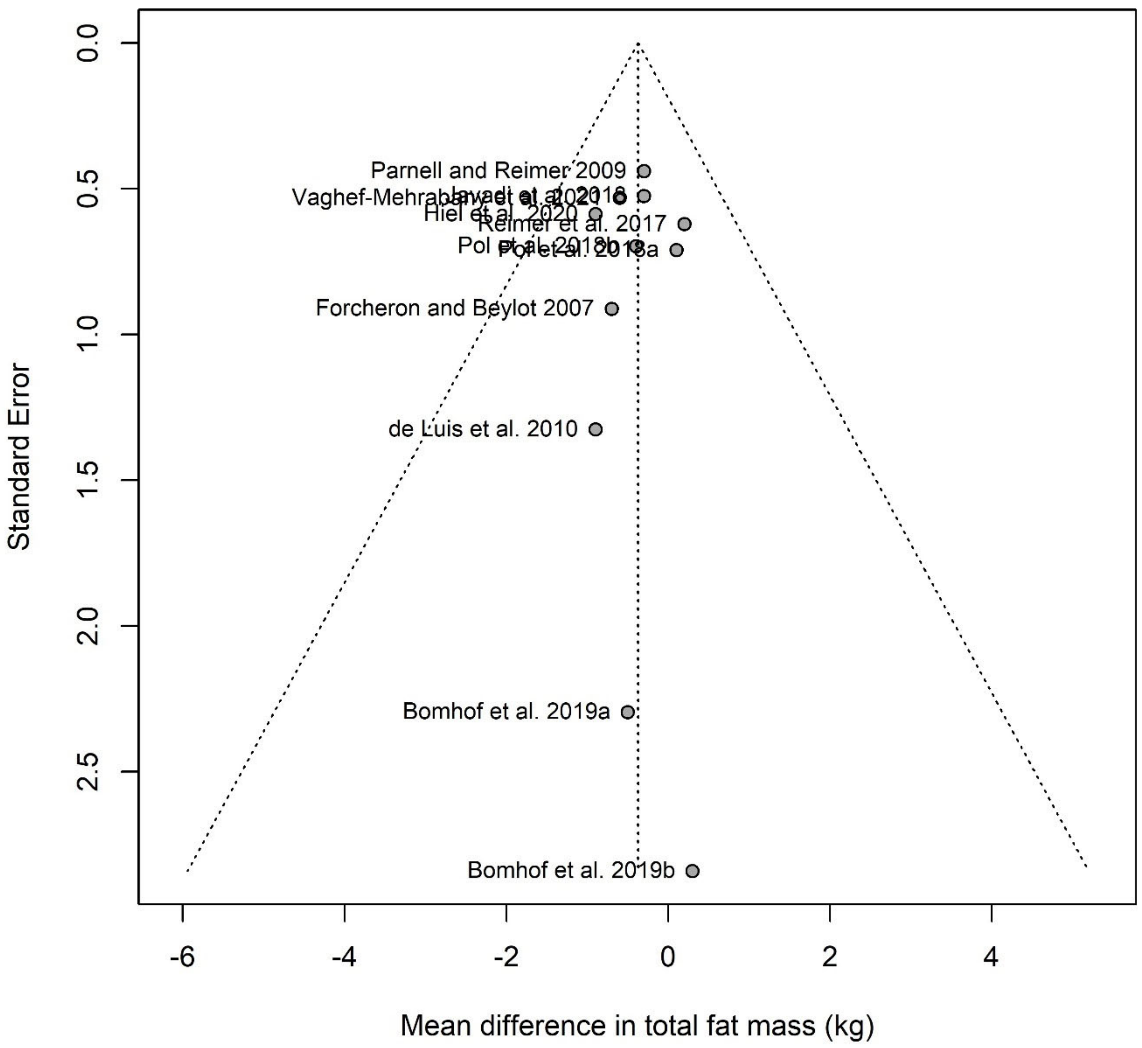


**Supplemental Figure 33** Funnel plot assessing publication bias for total fat mass (kg).

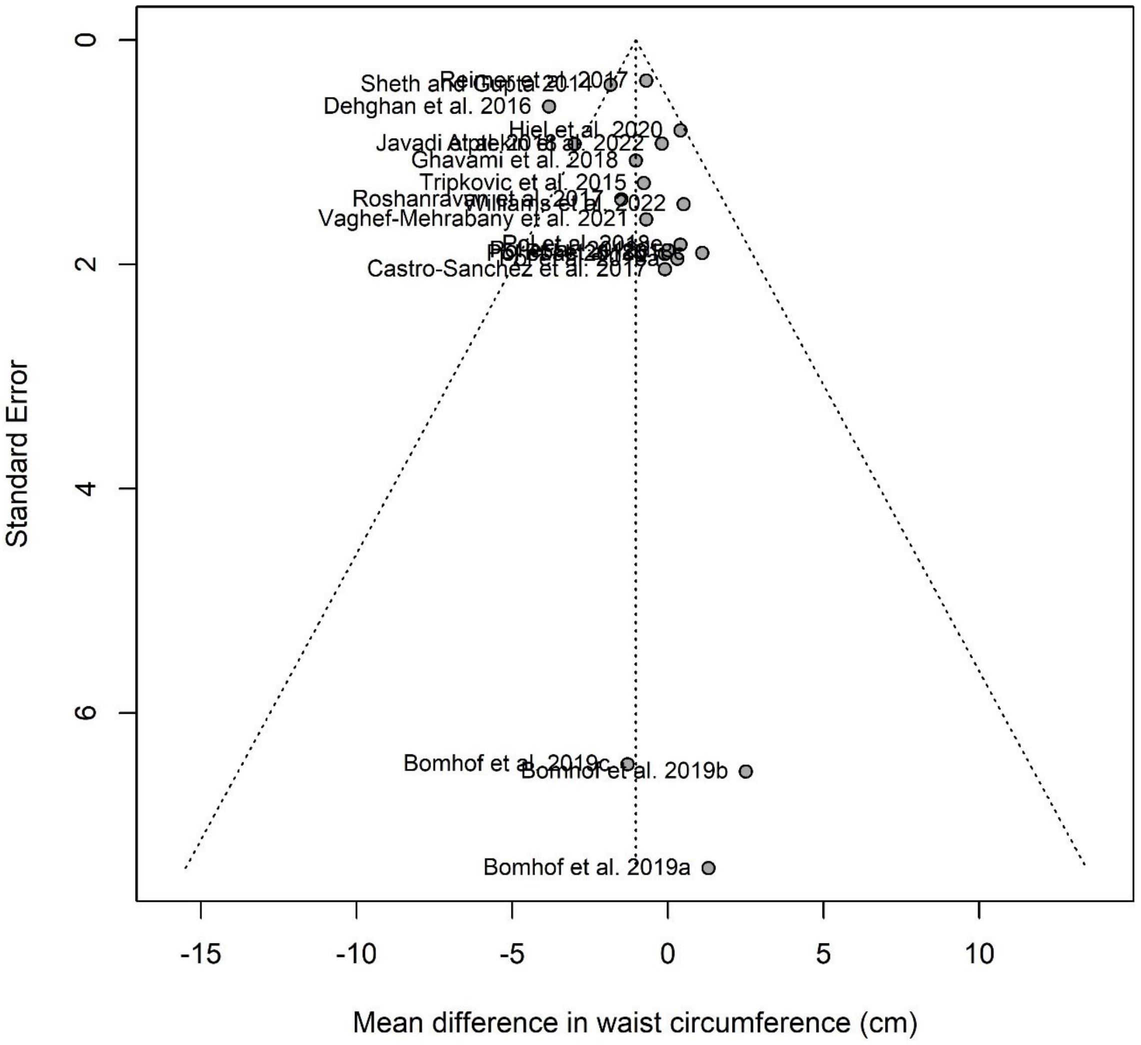


**Supplemental Figure 34** Funnel plot assessing publication bias for waist circumference (cm).

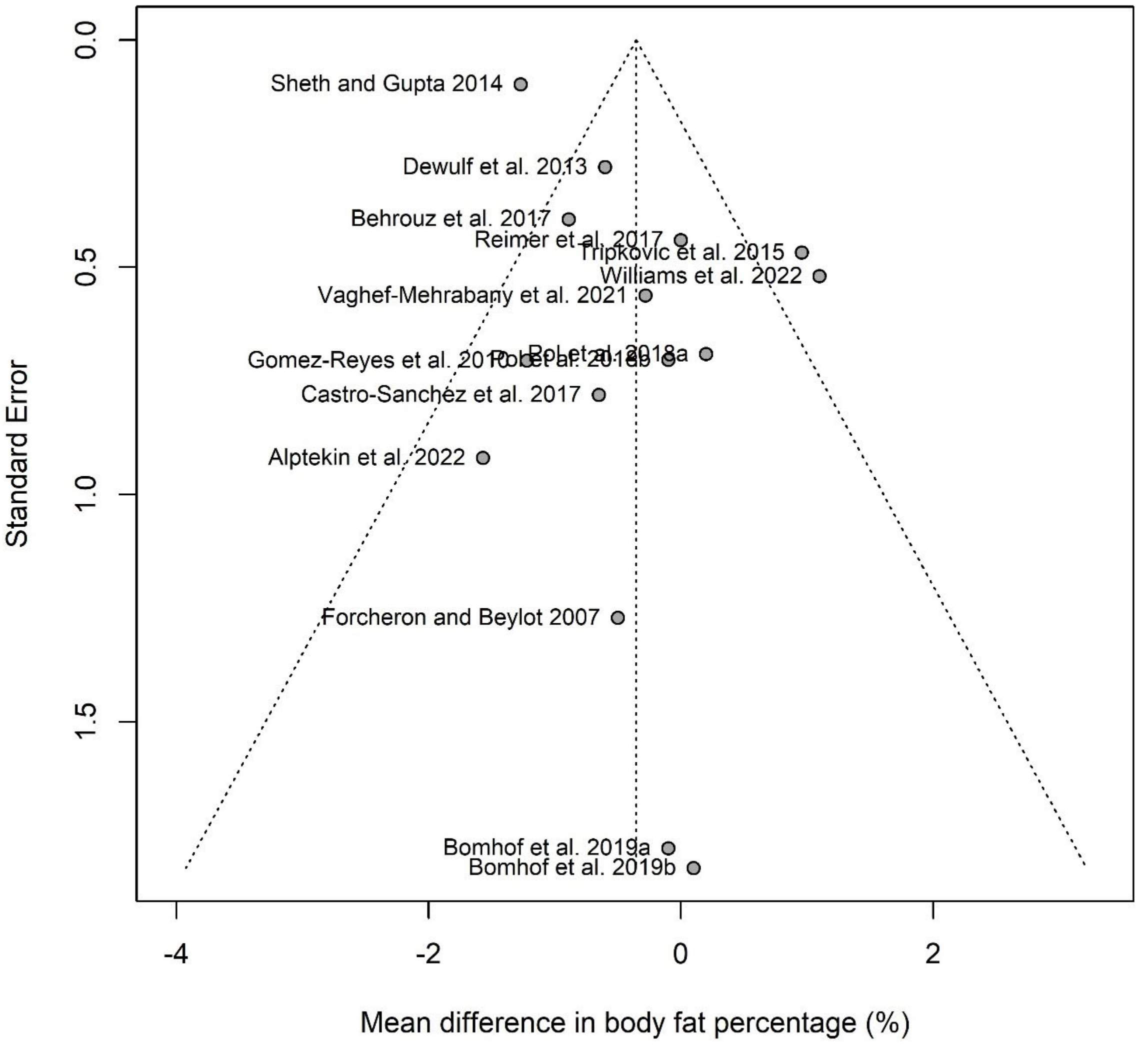


**Supplemental Figure 35** Funnel plot assessing publication bias for body fat percentage (%).

The effects of chicory inulin-type fructans supplementation on weight management aspects: systematic review, meta-analysis and meta-regression of randomized controlled trials.

Raylene A. Reimer, Stephan Theis, and Yoghatama Cindya Zanzer

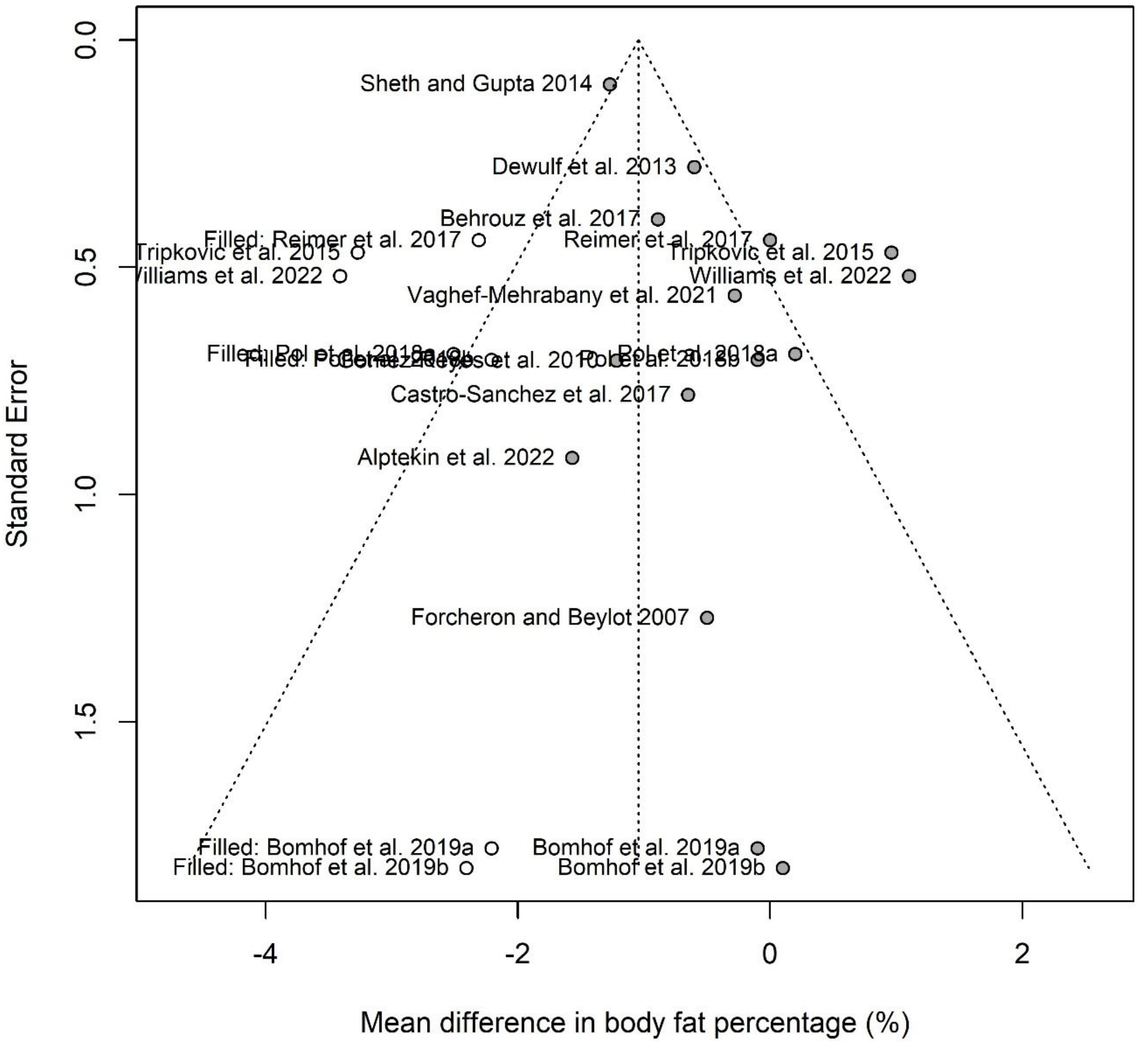


**Supplemental Figure 36** Funnel plot following Trim-and-Fill analysis for body fat percentage (%) outcome parameter.
